# Changes in the Urinary Proteome in a Patient-Derived Xenograft (PDX) Nude Mouse Model of Colorectal Tumor

**DOI:** 10.1038/s41598-019-41361-4

**Published:** 2019-03-21

**Authors:** Yongtao Liu, Youzhu Wang, Zhixiang Cao, Youhe Gao

**Affiliations:** 1 Gene Engineering Drug and Biotechnology Beijing Key Laboratory, Department of Biochemistry and Molecular Biology, College of Life Sciences, Beijing Normal University, Beijing, China; 2 Beijing Percans Oncology Co., Ltd, Beijing, China

## Abstract

In this report, the urinary proteome from a patient-derived xenograft (PDX) model was examined at the peptide level to study the origins of urinary proteins in tumor-bearing nude mice. Urine was collected from PDX mice before and after colorectal tumor implantation. A total of 4,318 unique peptides were identified, and 78 unambiguous human-origin peptides were discerned in the PDX model urine. Unlike the differential urinary protein composition of tumor-bearing immunocompetent rat models, the differential urinary proteins in the PDX model did not include host immune-response proteins. This study demonstrates that tumor-secreted proteins can be observed in the urine proteome of the PDX model. However, immune-response proteins, which are very early candidate tumor biomarkers, are not present in the urine of PDX model mice; this absence is due to immune deficiency. Therefore, immunodeficient animals may not be suitable models for searching for early immunity-associated tumor biomarkers in the urine.

## Introduction

Biomarkers are measurable changes associated with physiological or pathophysiological processes. Where as blood remains stable and balanced due to homeostatic mechanisms, urine, which is where most blood wastes are disposed, exhibits substantial changes^1^.

Many reports have found candidate biomarkers in the urine for the very early stages of disease. The identification of candidate biomarkers before the occurrence of amyloid plaque deposition has great importance for early intervention in Alzheimer’s disease (AD). Among the urinary proteins in 4-, 6-, and 8-month-old transgenic mouse models, 13 are associated with the mechanisms of AD, and 9 have been suggested as AD biomarkers^2^. In rats, pulmonary fibrosis-related proteins are detected in the urine before pulmonary fibrosis formation; if treatment is initiated at this time point, then prednisone, which is ineffective in the late stages, can effectively stop fibrosis development^3^. In a model of liver fibrosis, changes in urinary proteins occur earlier than those of aminotransferase and other indicators in the blood, and many of the changed proteins are associated with liver fibrosis, cirrhosis, and their formation mechanisms^4^. In a model of multiple sclerosis, changes in the urine proteome are observed before pathological changes occur. Among the 7 proteins with the most changes in this model, 6 have been reported to be associated with multiple sclerosis^5^. Changes caused by chronic pancreatitis can be reflected in early urinary proteins. New clues for the early diagnosis of chronic pancreatitis have been found in a small number of animal models^6^. Several urinary proteins have been shown to change in a rat model of chronic obstructive pulmonary disease (COPD), and some of the candidate markers are associated with COPD^7^. Differential urinary proteins have also been detected in the urine of a rat model of bacterial meningitis, many of which have been reported as biomarkers of bacterial meningitis in the cerebrospinal fluid and blood^8^.

The urinary proteome of rats subcutaneously injected with the Walker 256 tumor cell line has been shown to change significantly before a tumor mass is palpable. Some of the involved proteins have been reported as tumor markers or are associated with vaccine-related tumors^9^. Similarly, changes in the urinary proteome of rats injected with C6 glioma cells in the brain were observed before the tumors could be detected by magnetic resonance imaging (MRI). Many of these differential urinary proteins were previously reported to be associated with glioma^10^.

Since differential urinary proteins are present in the very early stages of tumor cell implantation, it is unlikely that the substantial changes in the urine result from a small number of tumor cells. Therefore, we investigated whether human proteins could be found in the urine of tumor-bearing nude mice. In the patient-derived xenograft (PDX) model^11^, human-origin tumors can grow due to the absence of normal T cell immunity. Human-origin peptides that are unambiguously identified in the urine of the model must originate from the human tumor. If mouse urinary proteins also change, then information about the pathophysiological changes in the host mouse may be reflected in these changes.

In this study, colorectal tumor tissues from patients were implanted subcutaneously into nude mice to establish a PDX model. Urine samples were collected from the nude mice before and after tumor transplantation. The samples were analyzed using bottom-up proteome analysis, and the urinary proteins were digested in gel and profiled by liquid chromatography tandem mass spectrometry (LC-MS/MS). The identified peptides were compared at the peptide level^12^.

## Materials and Methods

### Ethics approval

All experiments were performed using protocols approved by the Animal Care and Use Committee of the Beijing Normal University and Beijing Percans Oncology Co., Ltd., Review Board. All methods were carried out in accordance with relevant guidelines and regulations of the national health commission and the Ministry of Science and Technology. No human experiments were performed in this study, and no member of our research team named in the author list of this paper had access to identifying patient information when analyzing the data. Informed consent for the use of surgical waste tissue was obtained from all subjects.

### Tumor acquisition and PDX model establishment

The human tumor tissues used in this study were collected from surgical patients at Peking Union Medical College Hospital. All patients fully understood and signed informed consent forms. Pathogen-free, 8–10-week-old female NOD SCID mice and 10–12-week-old female BALB/c nude mice were purchased from Beijing Vital River Laboratory Animal Technology Co., Ltd. All animals were housed under temperature-controlled conditions with proper humidity, lighting (12 h light/12 h dark cycle), and free access to food and water. The animal experiments were reviewed and approved by the Institutional Animal Care and Use Committee (IACUC) and Animal Welfare Committee of Beijing Percans Oncology Co., Ltd. (Animal Welfare Assurance Number: IACUC2017004) and were performed in strict accordance with the guidelines of the IACUC of Beijing Normal University. All efforts were made to minimize animal suffering. Fresh tumor samples at least 6 × 6 × 6 mm (>200 mm^3^) in size were obtained *in situ* or from metastatic colorectal tumor tissue after surgery. The sampling sites showed highly malignant tissue activity. After collection, the samples were repeatedly rinsed with precooled sterile saline and immediately placed in precooled specialized preservative solution. The patient tumor tissues were placed individually into plates containing RPMI 1640 medium (Gibco, Paisley, UK) and transported to the laboratory at 4 °C. The tumor tissues were cleaned, and connective tissue, blood vessels, adipose tissue, and regions of calcification and necrosis were removed from the surface. Then, each tumor tissue was cut to obtain a 3–5 mm tumor mass. The tumor masses were implanted at 4 subcutaneous points in each of two female NOD/SCID mice. Tumor growth was observed daily. When the tumors reached a threshold volume, the tumor-bearing mice were sacrificed, and the tumors were dissected. Next, the tumors were cleaned, cut into small pieces of 3 × 3 × 3 mm and inoculated subcutaneously into twenty female BALB/c nude mice^13–15^. Each mouse was inoculated at 1 point. After 23 days, when the tumor volume had reached 300 mm^3^, urine was collected from the BALB/c nude mice.

### Urine collection and sample preparation

Animals were placed individually in metabolic cages overnight (for 12 h) to collect urine samples. During urine collection, the mice had free access to water but not food to avoid urine contamination. The collected urine was centrifuged at 4 °C and 3,000 × g for 10 min to remove cells and particulate matter and then stored at −80 °C. Centrifugation of the samples at 4 °C and 12,000 × g for 30 min was performed to remove cell debris before urinary protein extraction. The supernatants were precipitated with three volumes of acetone precooled at −20 °C for 2 h, followed by centrifugation at 4 °C and 12,000 × g for 30 min. Then, the precipitate was resuspended in lysis buffer (8 mol/L urea, 2 mol/L thiourea, 25 mmol/L DTT, and 50 mmol/L Tris (Sigma-Aldrich, Germany))^16^. The protein concentrations were measured using the Bradford assay at 595 nm. An albumin standard (Thermo Fisher, US) was used [bovine serum albumin (BSA) at 2 mg/mL in 0.9% saline and 0.05% sodium azide].

### Tryptic digestion in gels

Before each tumor-bearing nude mouse was inoculated with the tumor, urine was collected as a control. Urine samples from eight mice from each of the control and tumor-bearing groups were collected and analyzed by mass spectrometry with in-gel digestion. Eighty micrograms of total protein were mixed with 5 × loading buffer. The proteins were completely denatured at 95 °C for 10 min and loaded into 4–12% Bis-Tris preformed protein gels (Life, NuPAGE) in 1 × MES SDS running buffer (Life, NuPAGE). Then, the proteins were separated by electrophoresis at 200 V for 40 min. The gels were dyed with Coomassie blue for a few minutes to allow easier fading. Each gel was decolorized 2 to 3 times in a solution containing 30% methanol and 10% acetic acid until the gel background had lost as much of the blue stain as possible. The entire process was carried out in a container, and the same chemical reagents and processing time was used for all the gels to ensure the results of multiple gels could be reliably compared. Each lane was cut from the bottom up into 5 pieces based on the concentration in the gel, and each section was cut into 1 to 1.5 mm^3^ pieces. The pieces were washed with 25 mmol/L ammonium bicarbonate/acetonitrile solution (1:1 V/V) until they were thoroughly discolored. Dithiothreitol (DTT) was applied at 20 mmol/L and 37 °C for 1 h to denature the disulfide bonds in the protein structure, and 55 mmol/L iodoacetamide (IAM) was added for 30 min in the dark to alkylate the disulfide bound sites. Next, 5 μg/L of trypsin (Trypsin Gold, Promega, Fitchburg, WI, USA) was added to the dried gel pieces, which were then incubated at 37 °C overnight. Peptides were collected with 50% acetonitrile; then, the process was repeated, and the proteins were lyophilized and stored at −80 °C.

### Nano LC-MS/MS analysis

The reconstituted peptides were desalted with a C18 Zip-Tip (Millipore, Germany) and dissolved in 0.1% formic acid. LC-MS/MS analysis was performed using an EASY-nLC 1200 system coupled to an Orbitrap Fusion Lumos mass spectrometer (Thermo Scientific). The liquid chromatography parameters were as follows: precolumn: 75 μm × 2 cm, nanoViper C18, 2 μm, and 100 Å (Thermo Fisher Scientific, USA); analytical column: 50 μm × 15 cm, nanoViper C18, 2 μm, and 100 Å; and injection volume: 2 μL. The flow rate was 250 nL/min. Phase A was 0.1% formic acid/water (Fisher Scientific, Spain), and phase B was 80% acetonitrile (Fisher Chemical, USA)/0.1% formic acid/20% water. The ion origin was nano ESI, and MS_1_ data were collected by Orbitrap with a resolution of 120,000, an ion charge range of 2–7, and high-energy collisional dissociation (HCD) by applying a normalized collision energy of 32%. MS_2_ data were collected by Orbitrap at a resolution of 30,000. The HeLa protein digest standard (Thermo Scientific, 88329) was used to evaluate instrument performance.

### Data analysis

The raw MS data were processed using the Proteome Discoverer platform (Thermo Scientific, version 2.1) and the Sequest HT algorithm. Proteomic data were searched using the UniProt *Homo sapiens* and *Mus musculus* databases (updated Sep 2017). The search parameters were set as follows: the MS deviation of the peptide precursor and product ions was 0.05 Da; variable modifications included oxidation (M) and protein N-terminal acetylation and deamidation (N and Q); fixed modification included carbamidomethylation (C); and two missing trypsin cleavage sites were allowed. The peptide false discovery rate (FDR) was less than 1%.

### Bioinformatics analysis

Urine sample information for the nude mice was obtained from the online freeware DAVID Bioinformatics Resources 6.8 (https://david.ncifcrf.gov) for functional annotation assessment, including annotation of protein molecular functions, cell components, and biological processes.

## Results

### Nude mouse PDX model and tumor growth

**Figure 1:**
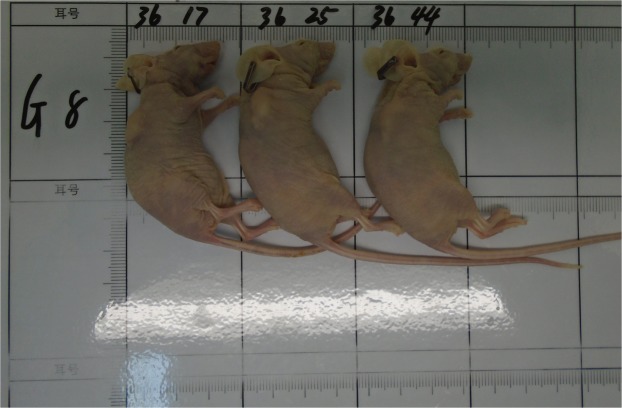
Image of mice of the patient-derived xenograft colorectal tumor model after sacrifice. Tumor tissue was implanted and growth subcutaneously in the right rear forelimb in BALB/c mice.

One of the patient colorectal tumor tissues was successfully established xenograft tumors in two NOD/SCID mice. These xenograft tumors were then dissected and inoculated subcutaneously into twenty female BALB/c nude mice,16(80%) were successfully established in BALB/c nude mice. Collect the urine of 13 in 16 mice for follow-up study. The body weights of the tumor-bearing BALB/c nude mice had little relationship with tumor growth. As tumor size increased, net mice weight declined, but total weight remained stable at an overall level of approximately 19–23 g. The tumor volumes were approximately 300 mm^3^ at 23 days after transplantation (Fig. 1).

### SDS-PAGE analysis of the model urinary proteins

**Figure 2:**
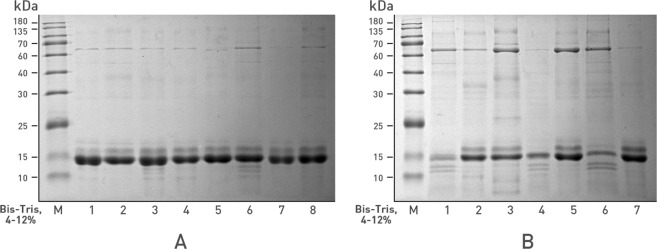
SDS-PAGE analysis of nude mouse urinary proteins. (**A**) Before-tumor-transplant group (n = 8); (**B**) tumor-bearing group (n = 7). Protein ladder is from 10 kDa to 180 kDa.

Sodium dodecyl sulfate polyacrylamide gel electrophoresis (SDS-PAGE) was used to compare differences in urinary proteins between the tumor-bearing and control mice (Fig. 2). The urinary proteins distributed from 10 kDa to 120 kDa in the nude mice, with a high-abundance protein(s) at 14 kDa. Based on this SDS-PAGE result, 8 samples were selected from the tumor-bearing (n = 4) and control groups (n = 4), and the bands were extracted from the gels. Each lane of the gel was cut into 5 pieces, yielding a total 40 samples. After gel-based digestion, the proteins were digested with trypsin and analyzed by LC-MS/MS.

### Urinary proteome profiling at the peptide level

After processing the data with Proteome Discoverer 2.1 and the Sequest HT algorithm, the MS data (.raw files) were searched against the *Homo sapiens* and *Mus musculus* databases to obtain peptide sequences and protein descriptions.

**Figure 3:**
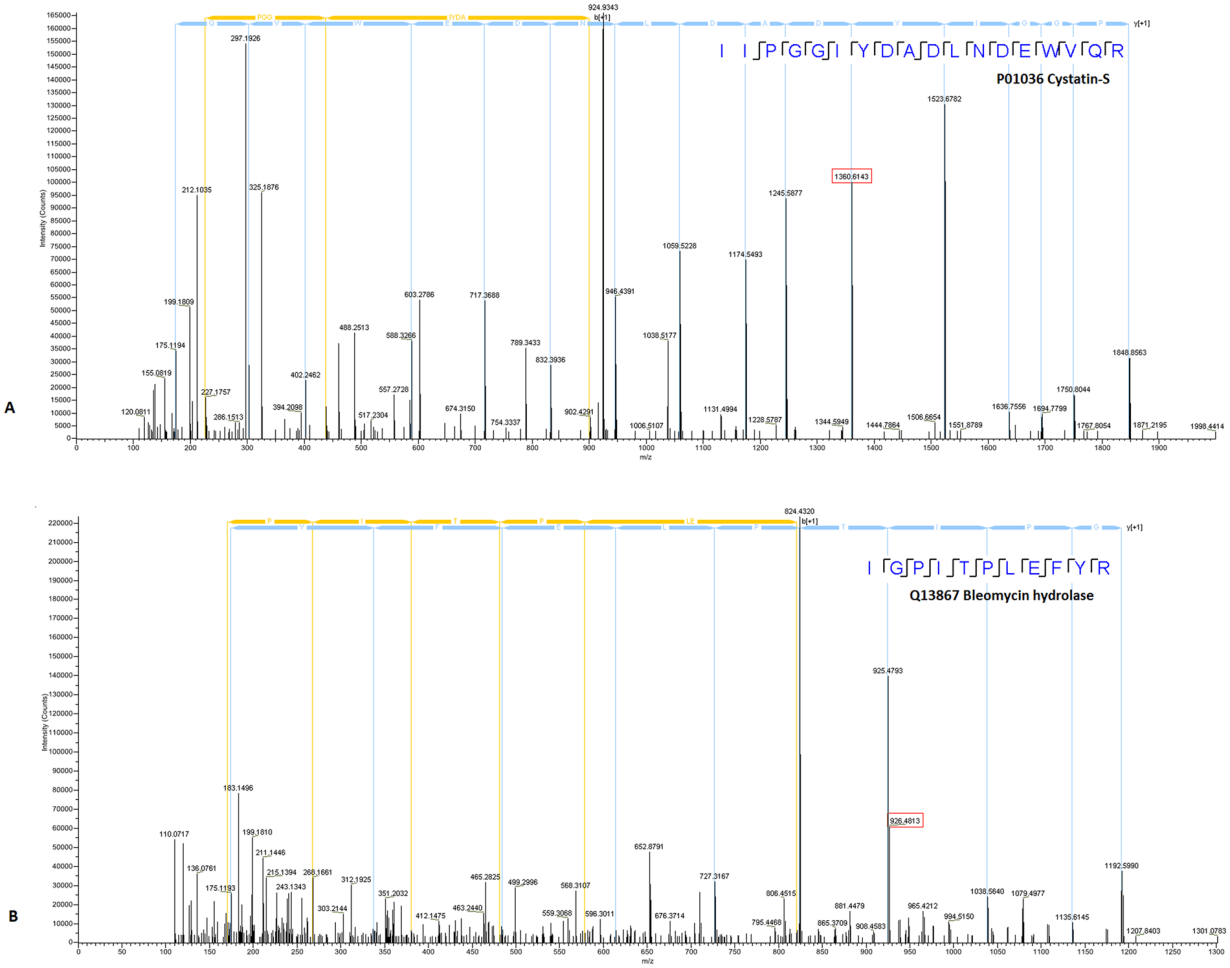
Partial displays of the MS_2_ of 78 human peptides. The tandem mass spectrometry fragmented the precursor ions of the peptides by HCD and produced different b^+^ and y^+^ product ion pairs. The peptide sequence information was obtained by calculating the mass-to-charge ratio (m/z) of adjacent fragment ions and matching with the database to obtain the species and protein descriptions of the peptides. Human peptides found by MS_2_ spectrum sequence associated with cystatin-s (**A**) and bleomycin hydrolase (**B**) are shown. The total spectrum is provided in Supplementary Information.

**Figure 4:**
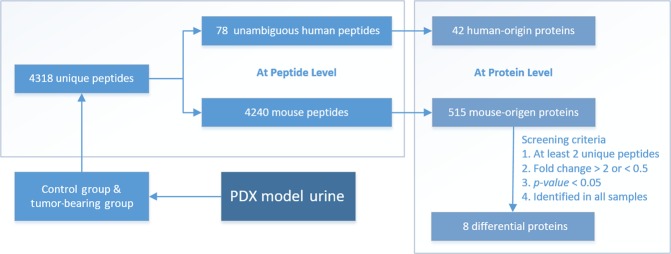
The main flow of data analysis. First, the species origin of the proteins was identified at the peptide level, and then the results were searched for human-origin unambiguous peptides. Subsequently, analysis was performed at the protein level. The peptide-level results were converted to protein-level results for the screening and comparison of differential proteins.

In total, 4,318 unique peptides were detected from the samples from the control and tumor-bearing groups. After manually comparing the homologous peptide sequences of mouse and human at the same protein locations (sequence data from UniProt, https://www.uniprot.org/), more than 3,800 peptides with same sequence for both human and mouse were removed. Seventy-eight unambiguous human peptides from 42 human proteins were identified with certainty among the 4,318 peptides, and each of these 78 peptides was confirmed on the MS^2^ spectrum (Fig. 3). The 78 peptides/42 human proteins were present only in the tumor-bearing mice; they were not detected in the control mice. The confidence of the peptide spectrum matches (PSMs) was greater than 99%, which was filtered by a 1% FDR. Figure 4 shows the main flow of the data analysis.

### Unambiguous human peptides and proteins

In the PDX model, 42 unambiguous human-origin proteins (corresponding to 78 human-specific peptides) were found in the urine on day 23 after tumor transplantation. These proteins must have originated from the human tumor.

Each human-origin protein was searched in the human urinary protein database (https://www.urimarker.com/urine/)^17^, which contains information on nearly 6,000 normal human urinary proteins. This database is available online and contains the most comprehensive information available on human urinary protein biomarkers. By searching this database, the abundance of each tumor-secreted protein in normal human urine was determined. The results are shown in Table 1.

**Table 1:** Human-origin tumor peptides and protein information from the tumor-bearing nude mice.

Accession no.	Protein description	Identification PSM counts	Unique peptide counts	Concentration in human urine (pg/mL)
B4DV14	highly similar to Napsin-A	69 (4/4)	1	not found
A1A508	PRSS3 protein (PRSS3)	44 (1/4)	1	not found
D9YZU5	Beta-globin (HBB)	33 (1/4)	6	73,156.39
P99999	Cytochrome c (CYCS)^*, †^	26 (3/4)	4	235.11
A8K7G6	highly similar to Homo sapiens Regenerating islet-derived 1 alpha	22 (1/4)	3	not found
A0A087WXI5	Cadherin-1 (CDH1)^*, †^	17 (3/4)	4	61,234.54
P48304	Lithostathine-1-beta (REG1B)^†^	14 (1/4)	1	not found
A0A0A6YYJ4	Trefoil factor 3 (TFF3)^†^	13 (2/4)	4	13,032.49
P04083	Annexin A1 (ANXA1)^*, †^	12 (1/4)	1	25,421.29
P01037	Cystatin-SN (CST1)	11 (1/4)	5	3,490.18
A0A024RAM2	Glutaredoxin (Thioltransferase) (GLRX)^*^	9 (2/4)	1	not found
P01036	Cystatin-S (CST4)	7 (1/4)	1	4,655.98
A0A1K0GXZ1	Globin C1 (GLNC1)	7 (1/4)	2	not found
S6B294	IgG L chain	7 (1/4)	1	not found
H9ZYJ2	Thioredoxin (TXN)^*, †^	7 (2/4)	2	not found
P36957	Dihydrolipoyllysine (DLST)^*, †^	6 (1/4)	1	4,061.93
V9HWA9	Epididymis secretory sperm binding protein Li 62p (HEL-S-62p)	6 (1/4)	2	not found
Q8TAX7	Mucin-7 (MUC7)	6 (1/4)	3	not found
Q6N092	DKFZp686K18196	6 (2/4)	3	not found
Q99988	Growth/differentiation factor 15 (GDF15)*^,†^	4 (2/4)	2	3,499.81
A0A1U9X8 × 6	CDSN	3 (1/4)	1	not found
P04406	Glyceraldehyde-3-phosphate dehydrogenase (GAPDH)*^,†^	3 (1/4)	1	20,395.34
P31151	Protein S100-A7 (S100A7)	3 (2/4)	2	1,600.63
Q96DA0	Zymogen granule protein 16 homolog B (ZG16B)	3 (1/4)	2	14,970.59
Q8N4F0	BPI fold-containing family B member 2 (BPIFB2)	2 (1/4)	2	752.72
A9UFC0	Caspase 14 (CASP14)	2 (1/4)	2	not found
Q76LA1	CSTB*^,†^	2 (1/4)	2	not found
P01040	Cystatin-A (CSTA)	2 (1/4)	2	1,309.68
Q05DB4	HEBP2	2 (1/4)	2	not found
A7Y9J9	Mucin 5 AC	2 (1/4)	2	not found
Q03403	Trefoil factor 2 (TFF2)^†^	2 (1/4)	1	59,910.81
Q13867	Bleomycin hydrolase (BLMH)^†^	1 (1/4)	1	262.38
Q8TCX0	Delta 2-isopentenyl pyrophosphate transferase-like protein	1 (1/4)	1	not found
V9HW80	Epididymis luminal protein 220 (HEL-S-70)	1 (1/4)	1	not found
B7Z3K9	Fructose-bisphosphate aldolase^*, †^	1 (1/4)	1	not found
Q6FH62	HSD17B3	1 (1/4)	1	not found
X6R7Y7	Intraflagellar transport protein 25 homolog (HSPB11)	1 (1/4)	1	not found
Q96P63-2	Serpin B12 (SERPINB12)	1 (1/4)	1	not found
P59665	Neutrophil defensin 1 (DEFA1)	1 (1/4)	1	20,375.74
P01833	Polymeric immunoglobulin receptor (PIGR)*^,†^	1 (1/4)	1	129,844.97
A0A158RFU6	RAB7^†^	1 (1/4)	1	not found
P29508	Serpin B3 (SERPINB3)	1 (1/4)	1	26,103.15

Note: * indicates that the protein was also identified among the Walker 256 rat model total proteins, and † indicates that the protein was also identified among the C6 glioma rat model total proteins. The contents in parentheses after “Identification PSM counts” are the number of animals exhibiting the protein/the total number of animals per group.

Of these 42 human proteins, 21 have been reported in normal human urine, including 16 high-abundance proteins (concentrations greater than 1,000 pg/mL), 3 moderate-abundance proteins (concentrations greater than 100 pg/mL), and 2 low-abundance proteins (concentrations less than 100 pg/mL); the remaining 21 proteins did not appear in normal human urine. Half of the 42 human tumor proteins have been detected in normal human urine. This finding indicated that these proteins, derived from tumor cells in this study, are found in normal human urine^18,19^. Table 1 provides information on the 42 human proteins, 78 specific peptides (See Supplementary Dataset 1), and their concentrations in normal human urine as determined by database search. Identification PSM counts are shown in the third column, and the ratio of the number of tumor-bearing mice in which the peptides were identified to the total mice number is presented in parentheses.

**Figure 5:**
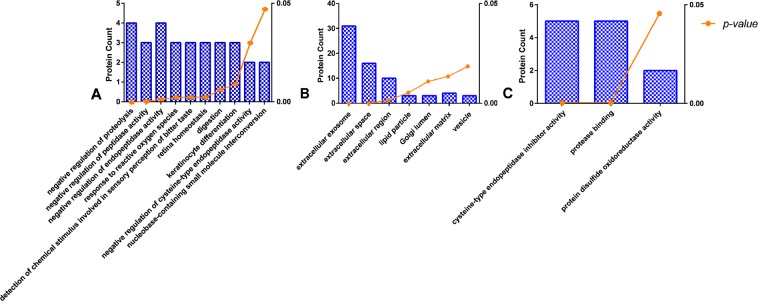
Functional analysis of human-origin tumor proteins in tumor-bearing nude mice: biological process (**A**), cellular component (**B**), and molecular function (**C**). The ordinate on the left is the protein count in the relevant pathway, and that on the right is the *p-value* of the corresponding pathway. A smaller *p-value* indicates a more significant relationship between the pathway and proteins.

The biological process analysis of the 42 human-origin proteins (Fig. 5A) identified numerous biological processes related to the digestion and hydrolysis of peptides or proteins (negative regulation of proteolysis, negative regulation of peptidase activity, negative regulation of endopeptidase activity, digestion, and negative regulation of cysteine-type endopeptidase activity) and responses to reactive oxygen species. In the cell composition analysis (Fig. 5B), most of the 42 human-origin proteins were derived from the secretion of extracellular exosomes or the extracellular space, region, or matrix rather than from the nucleus or cytoplasm. The analysis of molecular functions (Fig. 5C) showed that the common functions were related to protein structure and disulfide bonds. These are consistent with the secretion of proteases into surrounding tissues from a tumor during its growth, reproduction, and invasion. Proteases from a tumor can cause the hydrolysis of nearby proteins, which is beneficial for tumor growth and migration. The results of the bioinformatics analysis of the 42 human-origin proteins are shown in Fig. 5.

### Differential host nude mouse urinary proteins

The criteria used to screen differential murine proteins were as follows: (1) Each protein contains at least two unique peptides. (2) The *p*-value of each protein is less than 0.05, and the fold change is greater than 2 or less than 0.5. (3) The protein was identified in all samples from the group (4/4). A total of 515 host-associated proteins were identified, and 8 proteins met these identification criteria. Table 2 provides detailed information.

**Table 2:** The 8 differential urinary proteins in the nude mouse PDX model.

Accession	Protein description	Group(s) discovered in	Unique peptides (No.)	Fold change	*p*-value	Trend	Homologous protein
Q5FW60	Major urinary protein 20 (Mup20)	Control group & Tumor group	9	6.25	0.01051	Down	No
B0V388	Novel member of the major urinary protein (Mup) gene family	Control group & Tumor group	5	5.88	0.02104	Down	No
A2ARV4	Low-density lipoprotein receptor-related protein 2 (Lrp2)	Control group & Tumor group	77	2.73	0.04849	Up	Yes
P28843	Dipeptidyl peptidase 4 (Dpp4)	Control group & Tumor group	17	2.97	0.03824	Up	Yes
Q03265	ATP synthase subunit alpha, mitochondrial (Atp5a1)	Tumor group	3	—	—	—	Yes
G3XA48	Isopentenyl-diphosphate Delta-isomerase 1 (Idi1)	Tumor group	3	—	—	—	Yes
G3UYJ7	Predicted gene 20441 (Gm20441)	Tumor group	3	—	—	—	No
P52787	Gastric intrinsic factor (Gif)	Control group	5	—	—	—	Yes

The mouse differential protein information was compared with that from the Walker 256 tumor-bearing rat model^9^ and the glioma rat model^10^ (Table 3). This comparison revealed that the number of differential proteins in the PDX model was lower than the numbers in the other model tumor models under the same screening criteria.

**Table 3:** Differential urinary protein information from three tumor models.

		PDX model	Glioma model	Walker 256 model
Experimental animal		Nude mouse	Rat	Rat
Immune system		T cell immunodeficient	Immunocompetent	Immunocompetent
Days		23	13	14
No. of differential proteins		8	27	31
No. of total proteins		515	778	533
Screening criteria	No. of unique peptides	>2		
	Fold change	>2 or <0.5		
	*p*-value	<0.05		
	Number in group	4/4	3/3	4/4

### Differential urinary protein functional analysis of the hosts

Biological process analyses were performed with 31 differential proteins from the Walker 256 tumor-bearing rat model and 8 differential proteins from the PDX model. The 8 differential proteins from the PDX model were related only to the transport process (the protein count was 3, and the *p*-value was 0.083).

However, 32 biological processes were found in the Walker 256 tumor-bearing rat model, many of which were related to immunity, including the complement activation classical pathway, acute phase response, defense response to bacterium, positive regulation of B cell activation, phagocytosis recognition, innate immune response, phagocytosis engulfment, inflammatory response, negative regulation of tumor necrosis factor production, B cell receptor signaling pathway, Factor XII activation, apoptotic process, and immune system process (Table 4).

**Table 4:** Biological process analysis of the tumor-bearing rat and PDX nude mouse models.

Model	Biological process(es)	Count	*p*-value
Walker 256 (includes only those biological processes with *p*-values less than 0.05)	complement activation, classical pathway	5	8.40E-07*
	acute phase response	4	5.90E-05
	defense response to bacterium	5	1.40E-04
	negative regulation of endopeptidase activity	5	2.10E-04
	organ regeneration	4	7.80E-04
	positive regulation of B cell activation	3	8.70E-04
	phagocytosis, recognition	3	1.20E-03
	innate immune response	5	1.70E-03
	phagocytosis, engulfment	3	2.20E-03
	inflammatory response	5	2.80E-03
	negative regulation of tumor necrosis factor production	3	3.50E-03
	response to drug	6	3.70E-03
	B cell receptor signaling pathway	3	3.80E-03
	hemoglobin import	2	4.00E-03
	vitamin metabolic process	2	4.00E-03
	factor XII activation	2	6.00E-03
	cobalamin transport	2	8.00E-03
	proteolysis	5	1.40E-02
	positive regulation of dendritic cell chemotaxis	2	1.60E-02
	response to lipopolysaccharide	4	1.80E-02
	tissue remodeling	2	2.40E-02
	aging	4	2.50E-02
	carbohydrate metabolic process	3	2.60E-02
	lipoprotein transport	2	2.80E-02
	apoptotic process	4	3.60E-02
	immune system process	2	4.90E-02
PDX (all biological processes)	transport process	3	8.30E-02

*e.g., 8.40E-07 = 0.00000084.

In the urine from the tumor-bearing immunocompetent rat model, many differential proteins were associated with immune responses. In contrast to the rat model results, no host immune-response proteins were found in the PDX model urine. The presence of these immune-related proteins explains why the number of urinary differential proteins in the PDX model was less than that of tumor rat models. Perhaps these immune-related proteins can be used as early urine candidate biomarkers of tumors. Due to the magnification effect of the immune system, even a small number of exogenous tumor cells can cause a strong reaction of the host system, with changes in the urine magnifying the changes caused by tumor secretion. No immune-related proteins were found in the urine of the PDX model because nude mice are immunodeficient. Therefore, immunodeficient animals may not be suitable models for searches of immunity-associated early-tumor biomarkers in urine.

## Discussion

### Differentiation of protein origins from different species at the peptide level

The bottom-up proteomics method was used in this study, in which proteins are digested into peptides by trypsin. The MS/MS spectra can only identify peptides. Although humans and mice have very high genetic homology, many tryptic peptides are not the same for homologous proteins. Some peptides can be unambiguously identified as human peptides. However, biological function information is only available at the protein level. In functional studies, peptides are converted to their corresponding proteins. Therefore, the biological process analysis in this study was carried out at the protein level.

### Comparison of the 78 peptides/42 proteins with the human urinary protein database

Comparison of the identified peptides and proteins with the human urinary protein database revealed that 21 of the proteins have been reported in normal human urine, suggesting that these proteins may not be good biomarkers of this type of cancer in humans. Tumor cells are similar to normal cells, and it is rare that the proteins from tumors are unique to tumor cells. Most candidate biomarker proteins can also be observed in healthy cells. Thus, these proteins are not necessarily good biomarkers individually. Comparison of the experimental results with the data in the human urinary protein database and on urinary proteins in healthy individuals indicated that these 21 proteins can be derived from either tumor cells, as in the PDX model, or healthy human cells.

### Comparison of differential proteins between nude mice and rats

Few reports are available concerning biomarkers of very early tumors in the urine of animal models. Both the Walker 256 subcutaneous model and C6 glioma model were created in rats, whereas the PDX model is only available in mice. Rats and mice (nude mice) are different species, but their homologous proteins have similar biological functions, which makes cross-species comparisons at the protein function level meaningful. Since a panel of tumor-stimulated host immune proteins in the urine may be used as candidate early biomarkers, an immunodeficient animal model, such as nude mice, may not be suitable for discovery studies of very early-tumor biomarkers.

### Selection of the gel-based method for sample pretreatment

In this study, because of the very high abundance of some proteins in mouse urine (see Fig. 2), the gel-based method was used to eliminate the suppression of high-abundance proteins and improve the identification of low-abundance proteins. If the sample preparation procedures are carefully controlled, the gel-based analytical method is sufficiently reliable for quantitative analysis^20,21^. The disadvantage of this method is that contaminants can be easily introduced during the experiment. Common contaminant proteins were excluded in this study by comparing the results to the pollutant database^22,23^. Although gel slicing may not be the best quantitation method, the use of a different method would not have changed the conclusion of this study (i.e., that no immune-related host proteins were found in the PDX nude mouse model).

## Conclusion

The 78 human-origin peptides successfully identified in the urine from the PDX nude mouse model at the peptide level originated from a colorectal tumor. In contrast to those of tumor-bearing immunocompetent rat models, host immune-related proteins in the PDX model were absent from the differential urinary proteins. Therefore, an immunodeficient animal, such as the nude mouse, may not be a suitable model for screening early immunity-associated tumor biomarkers in urine.

## Supplementary information

Supplementary Information

Dataset 1
