# Supplementary material for: Changes in the Urinary Proteome in a Patient-Derived Xenograft (PDX) Nude Mouse Model of Colorectal Tumor
Source: Sci Rep. 2019 Mar 21;9:4975. doi: 10.1038/s41598-019-41361-4 (PMC6428931; doi:10.1038/s41598-019-41361-4)

**Supplementary Information**

Changes in the Urinary Proteome in a Patient-Derived Xenograft (PDX) Nude Mouse Model of Colorectal Tumor

Yongtao Liu^1^, Youzhu Wang^2^, Zhixiang Cao^2^, Youhe Gao^1*^

P04083 Annexin A1 (1/1)


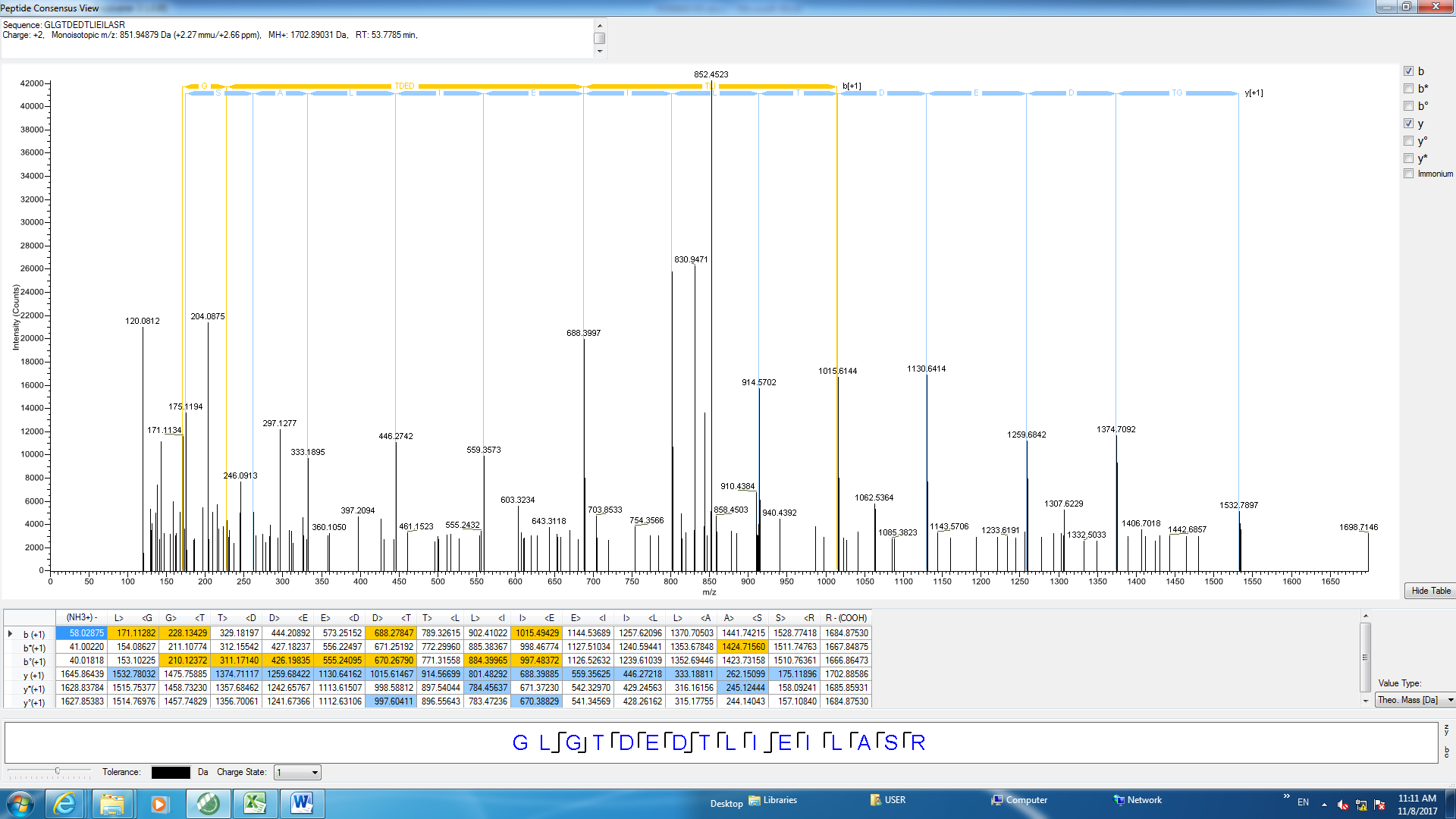


D9YZU5 Beta-globin (1/6)


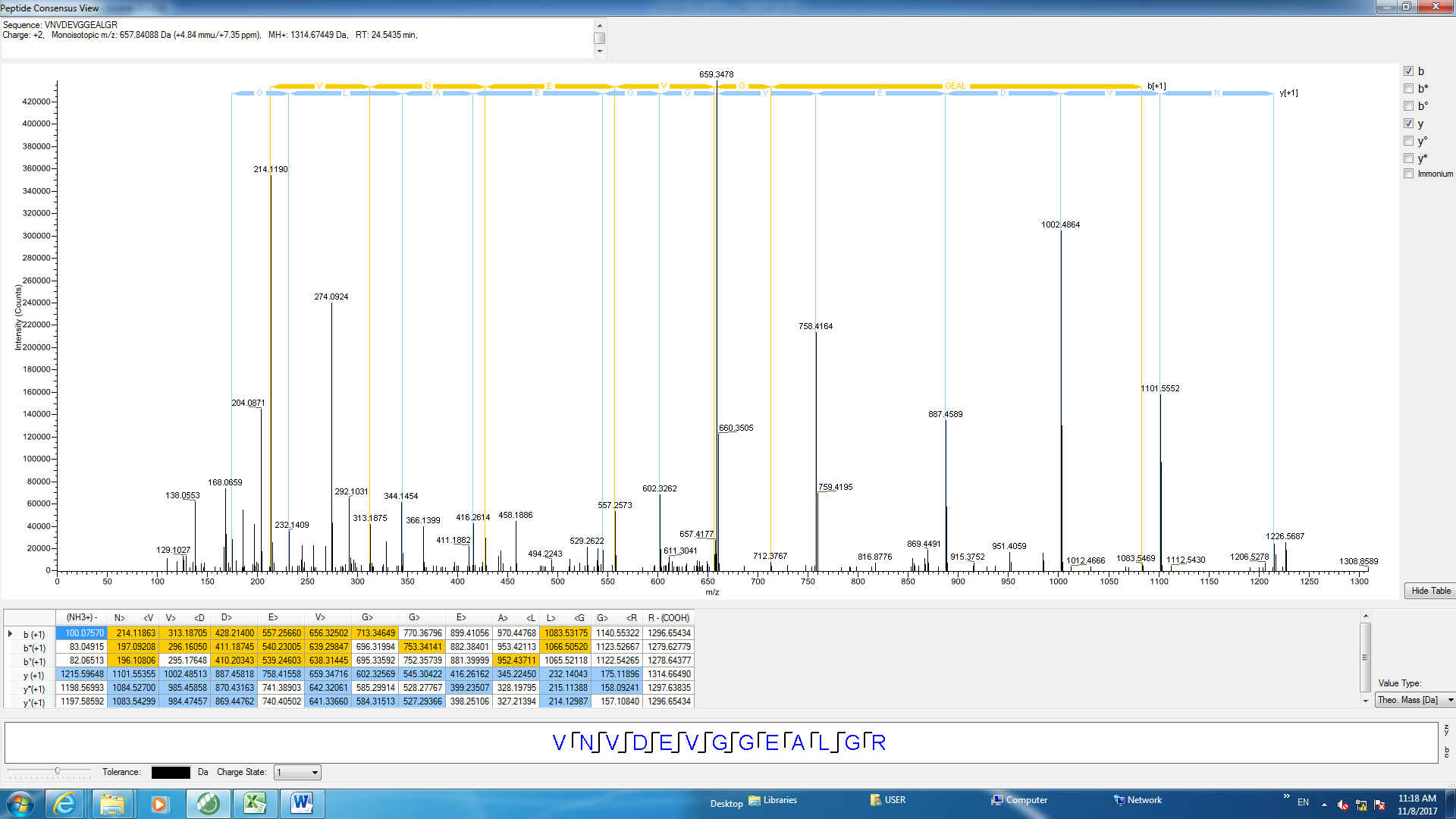


D9YZU5 Beta-globin (2/6)


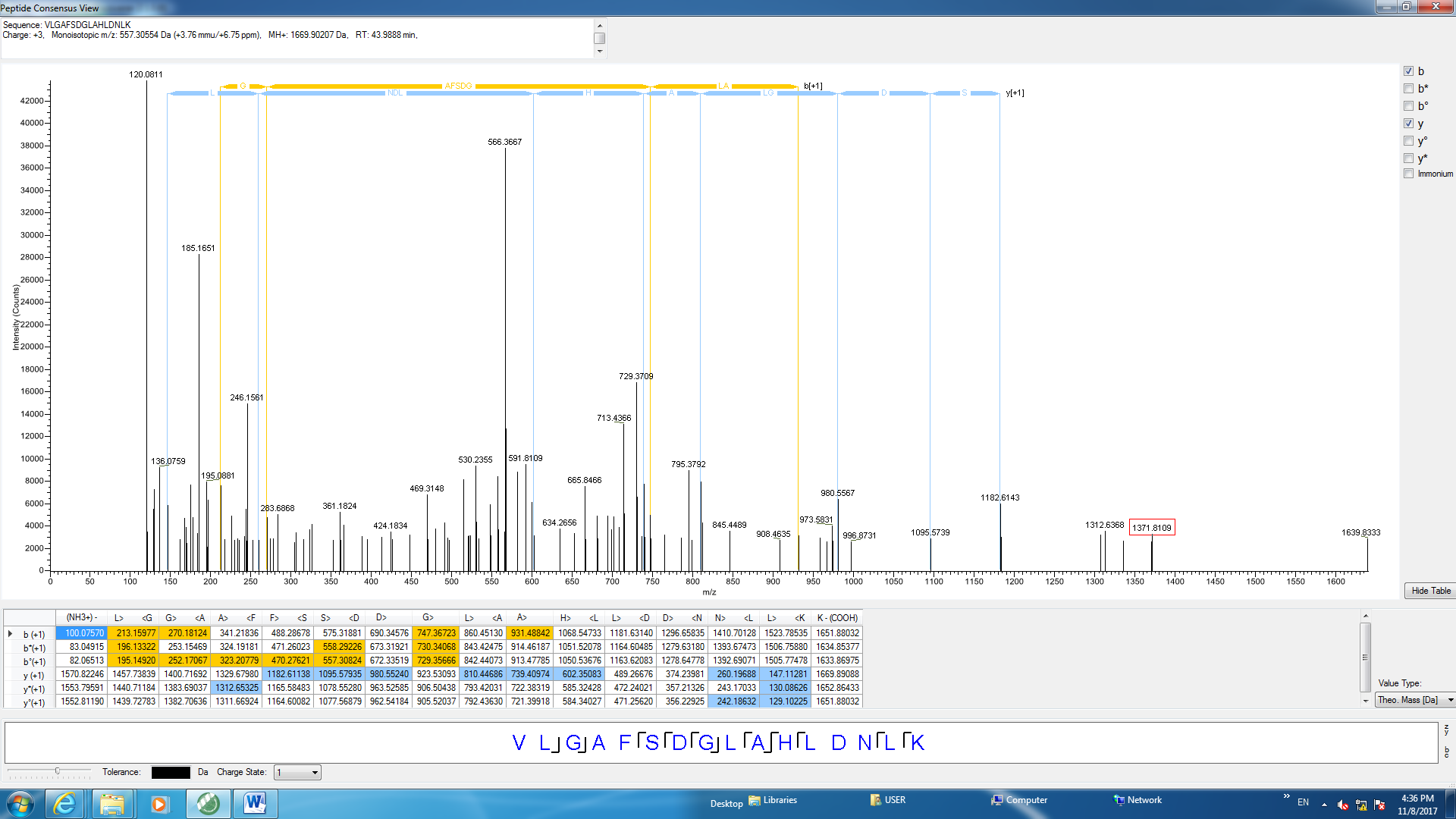


D9YZU5 Beta-globin (3/6)


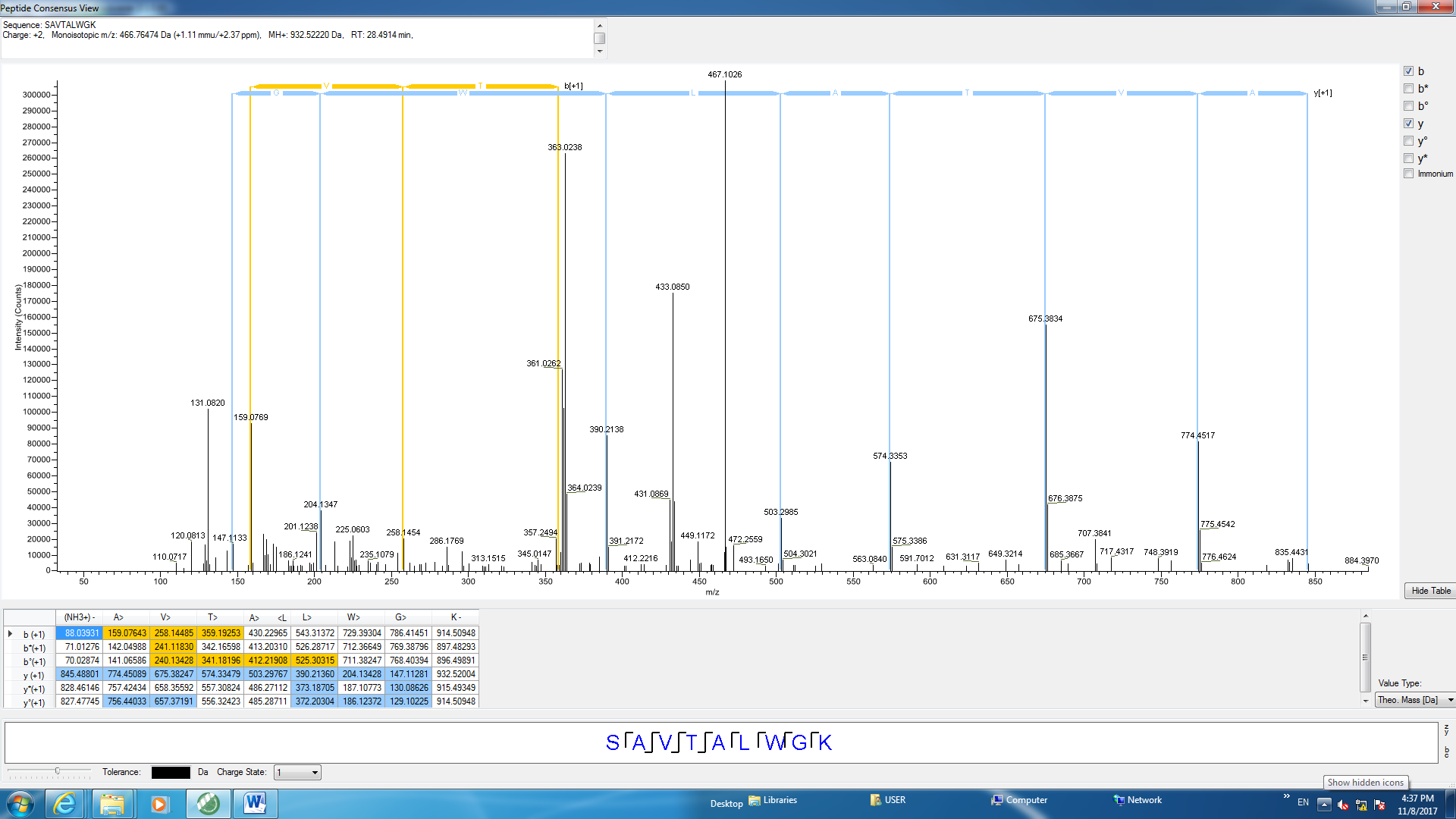


D9YZU5 Beta-globin (4/6)


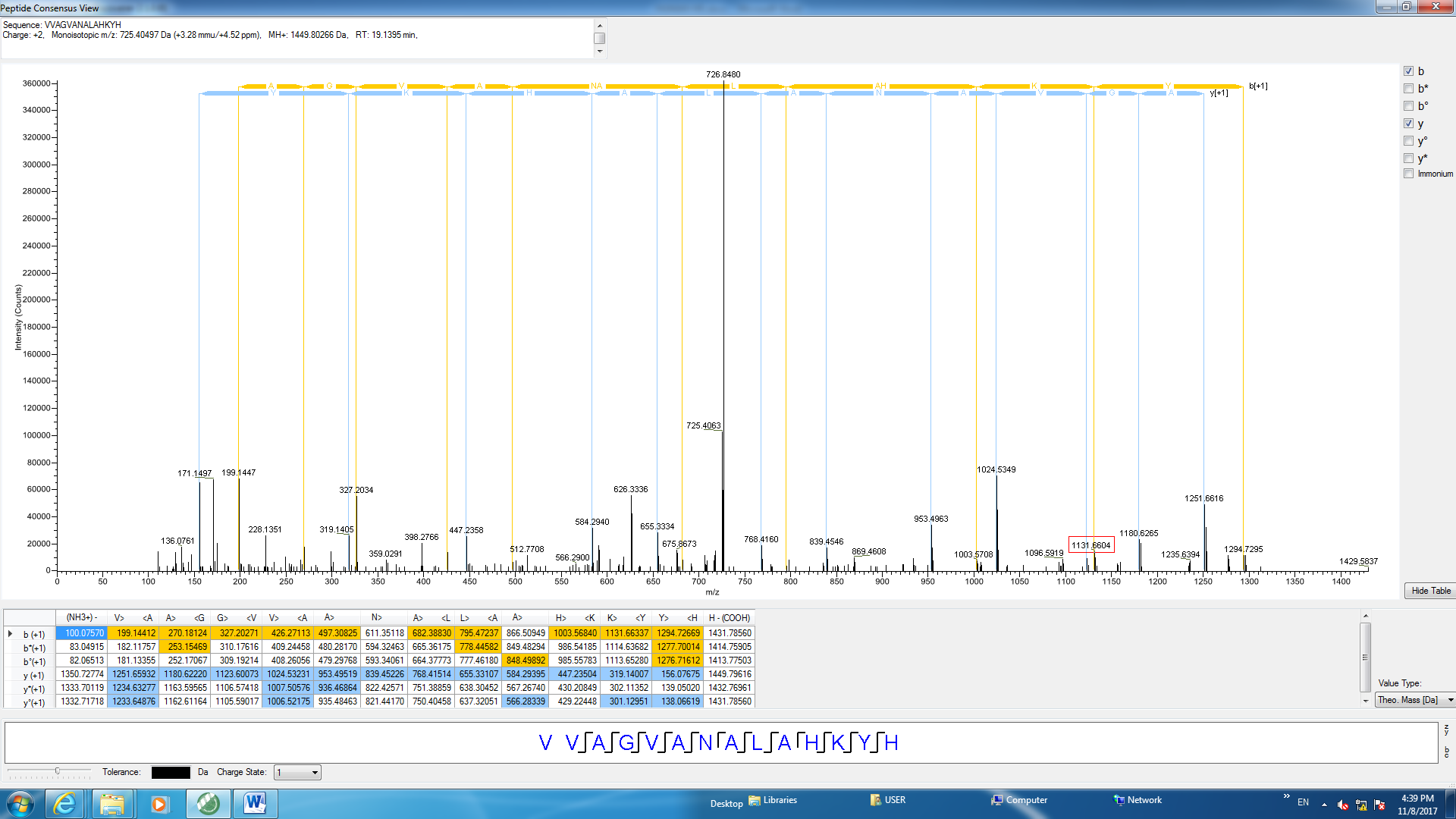


D9YZU5 Beta-globin (5/6)


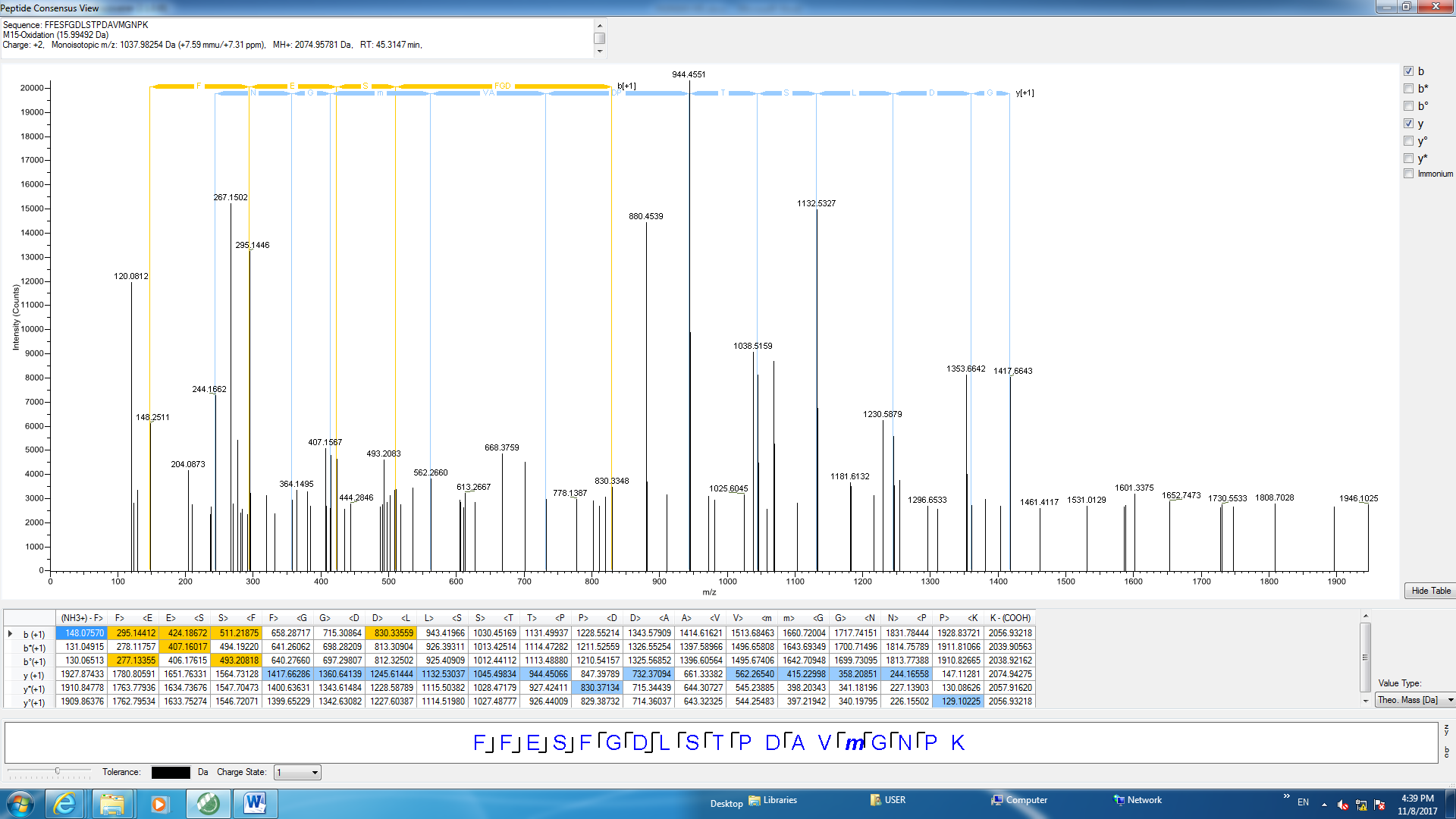


D9YZU5 Beta-globin (6/6)


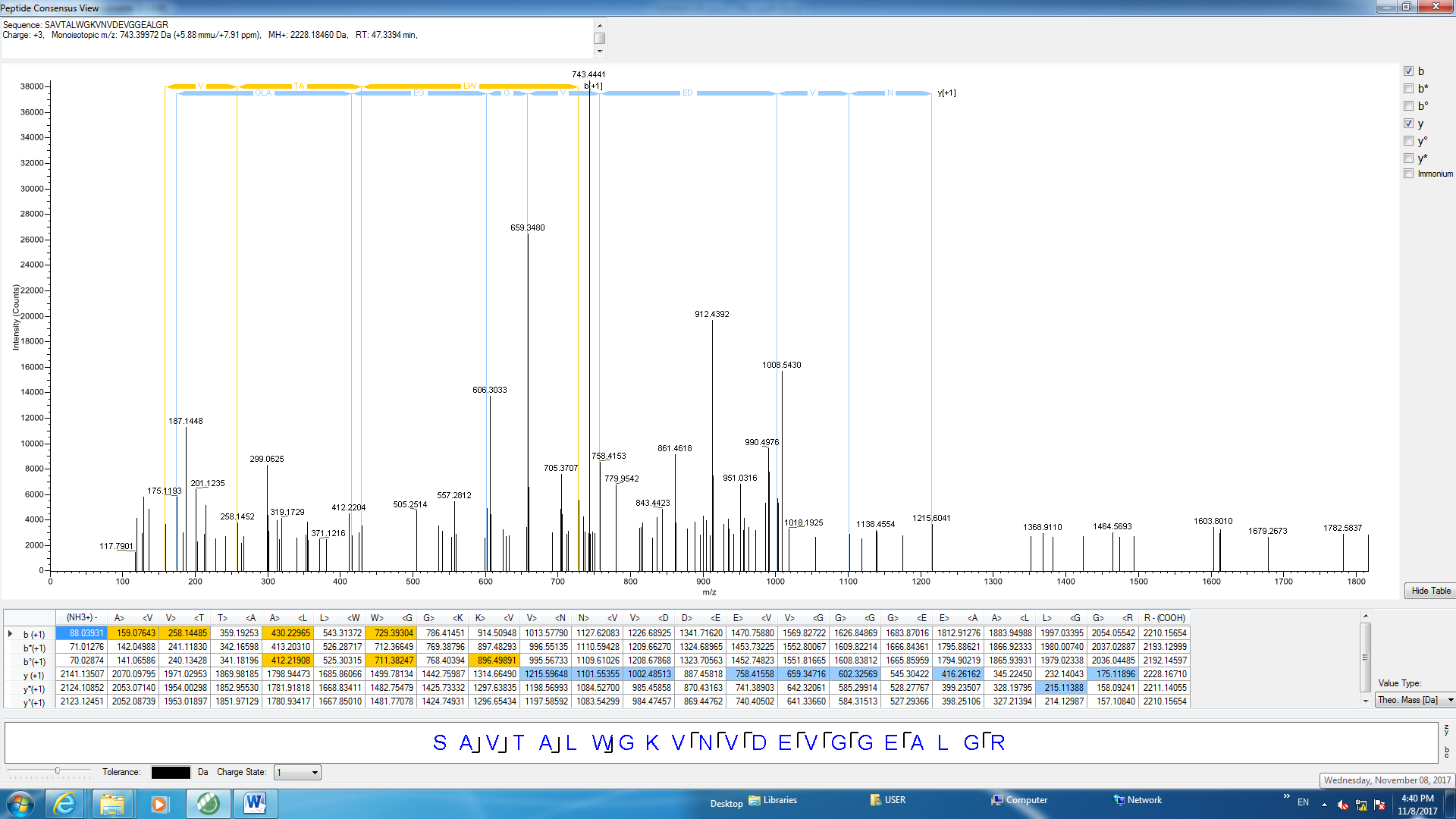


Q13867 Bleomycin hydrolase (1/1)


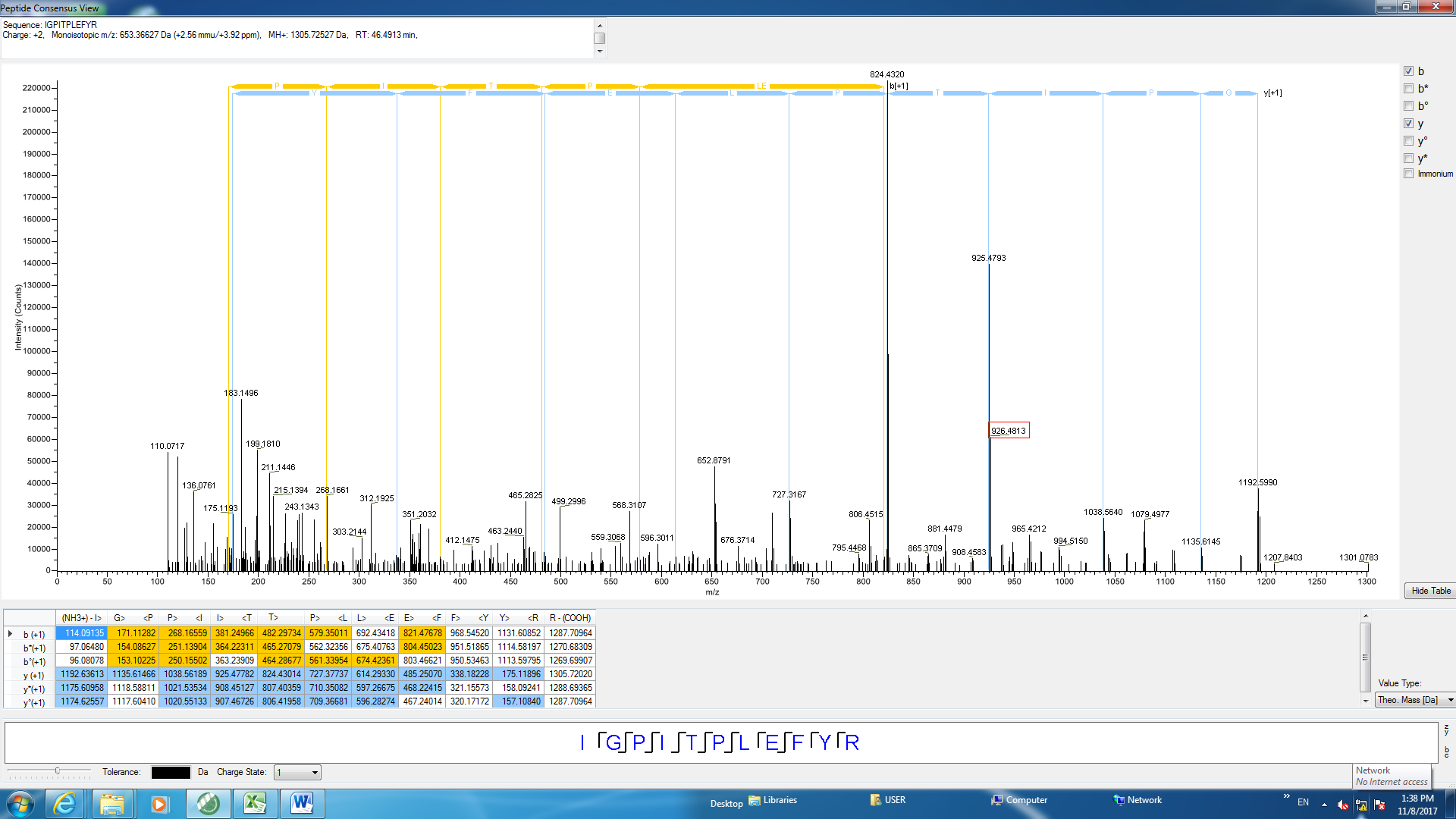


Q8N4F0 BPI fold-containing family B member 2 (1/2)


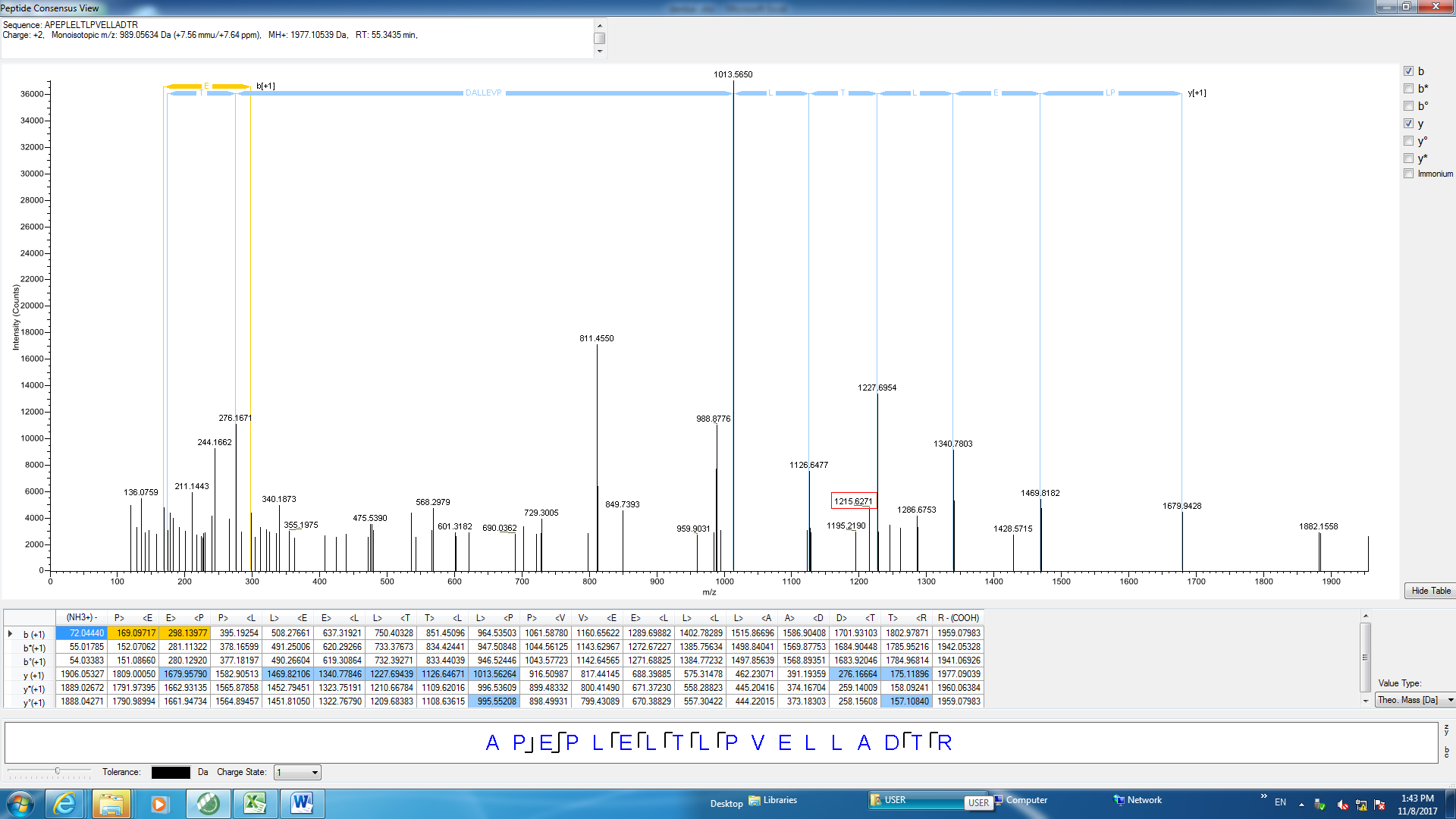


Q8N4F0 BPI fold-containing family B member 2 (2/2)


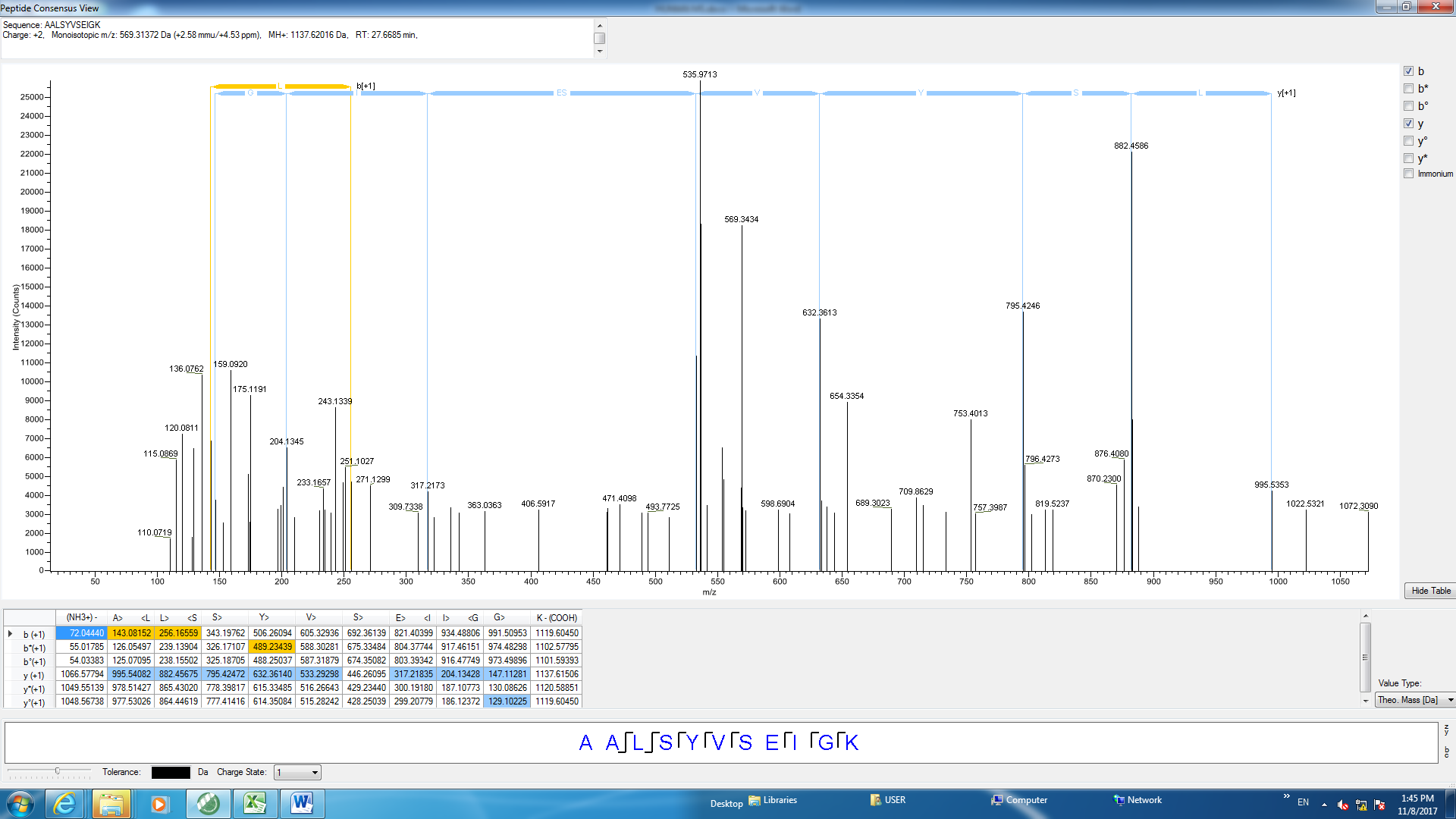


A0A087WXI5 Cadherin-1 (1/4)


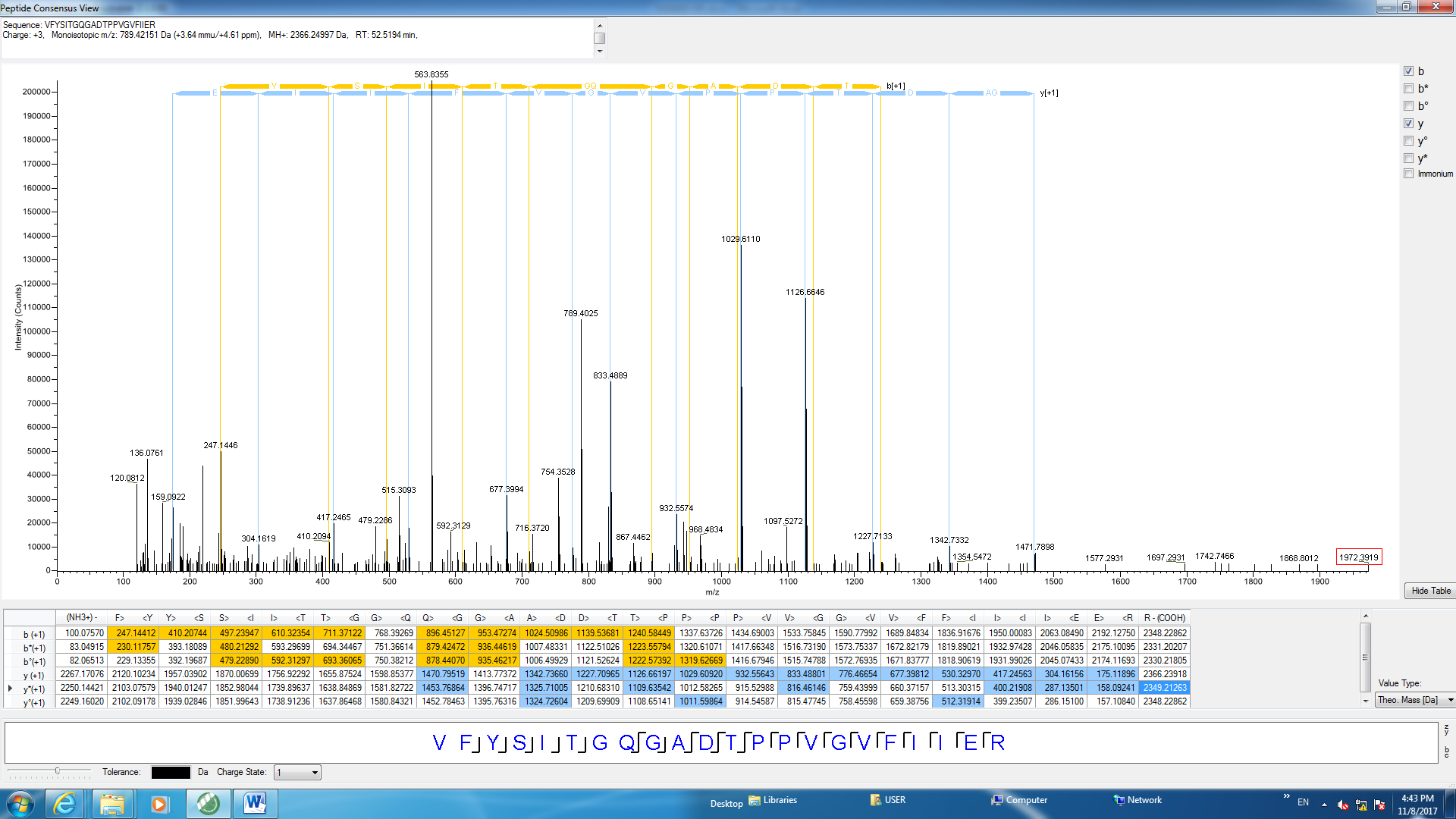


A0A087WXI5 Cadherin-1 (2/4)


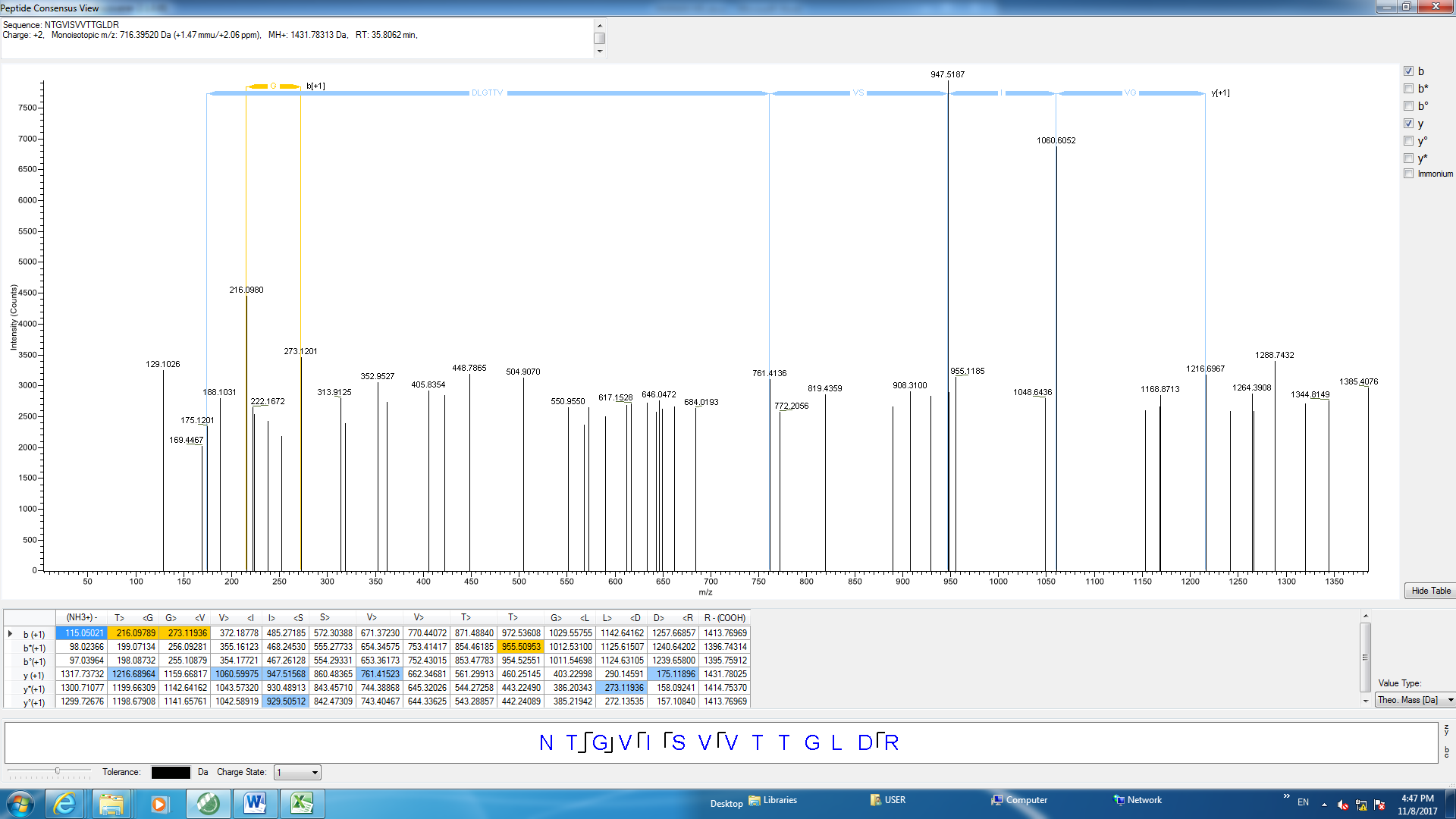


A0A087WXI5 Cadherin-1 (3/4)


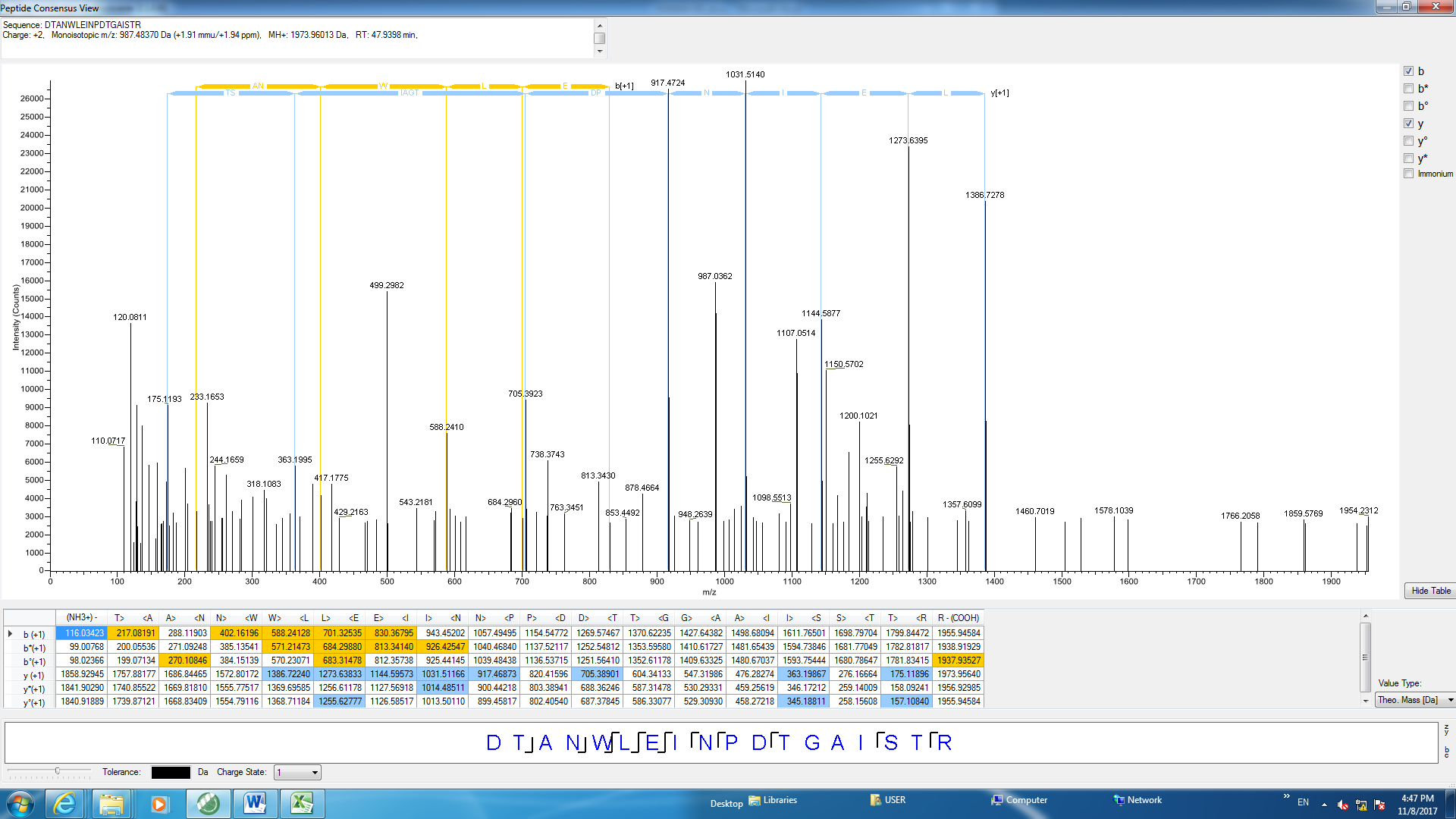


A0A087WXI5 Cadherin-1 (4/4)
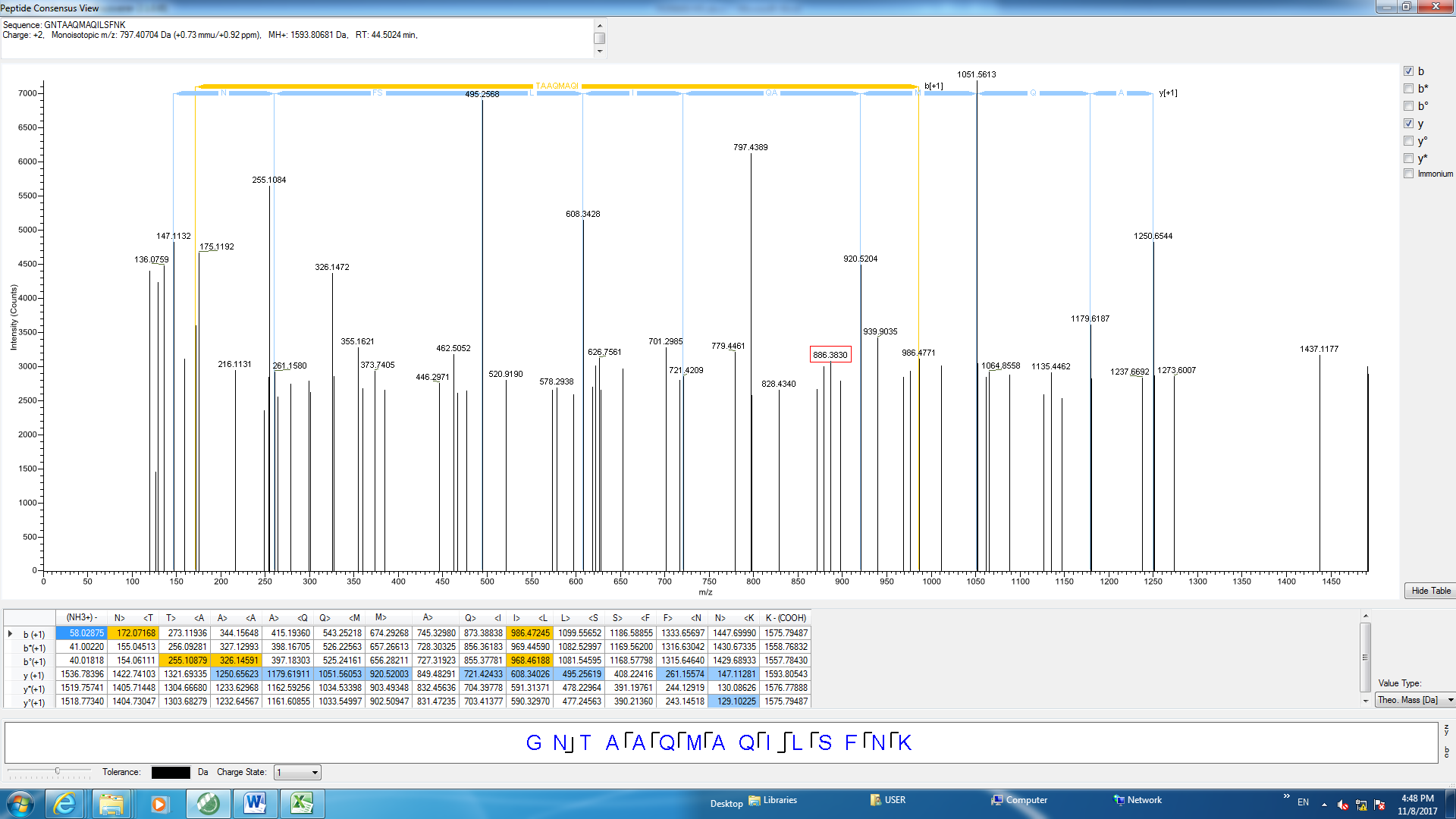


A9UFC0 Caspase 14 (1/2)


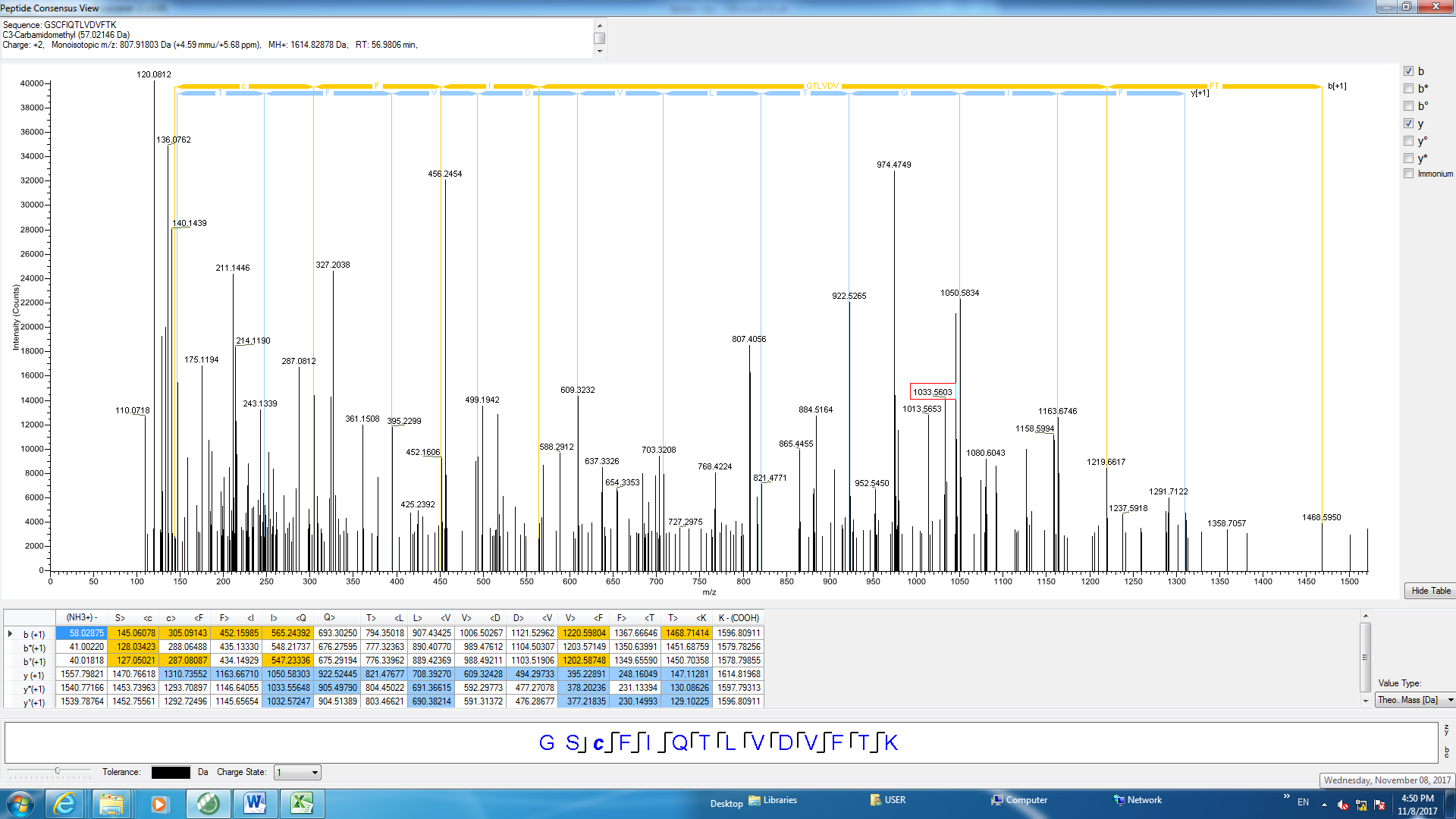


A9UFC0 Caspase 14 (2/2)


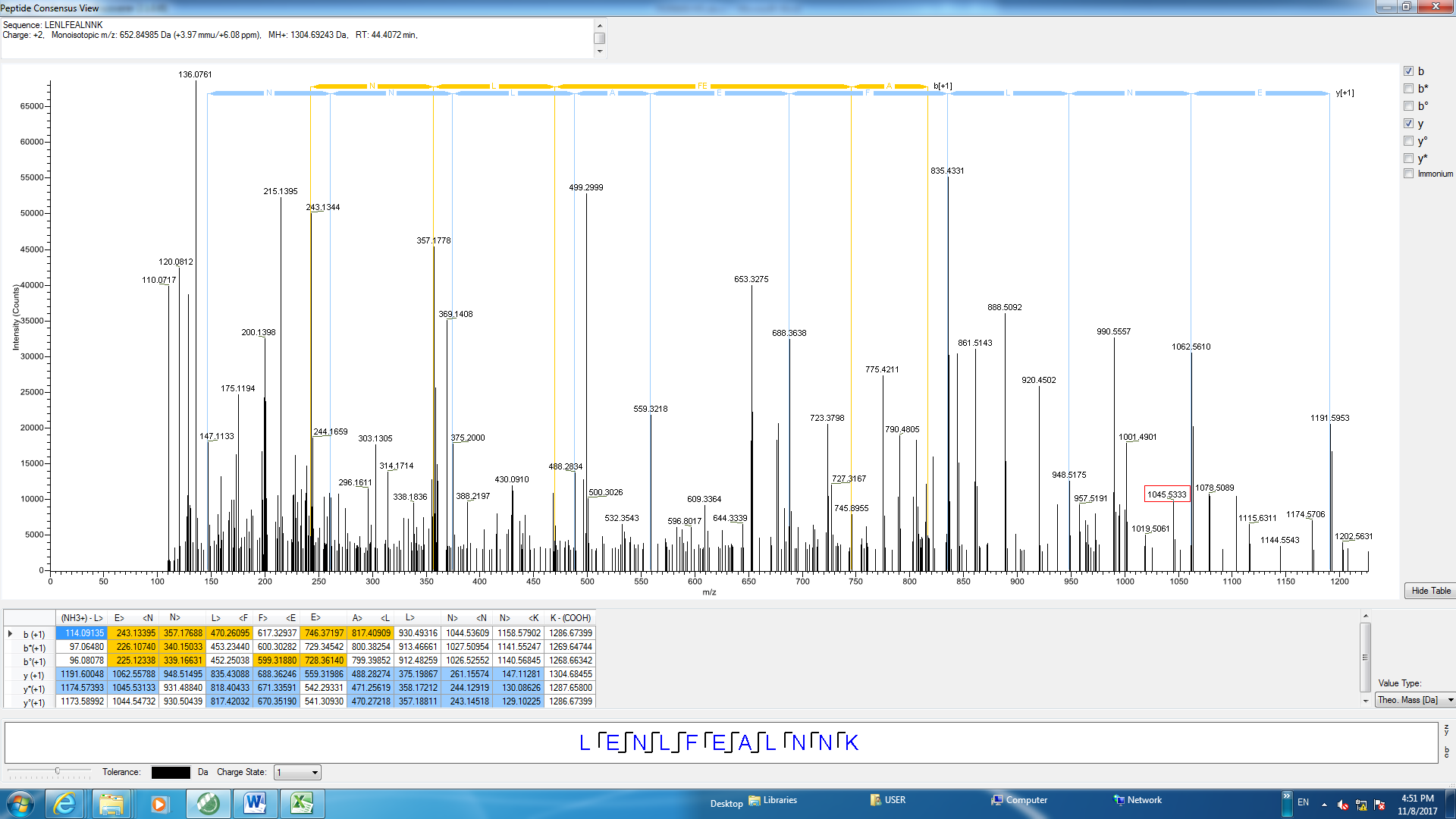


B4DV14 high similar to Napsin-A (1/1)


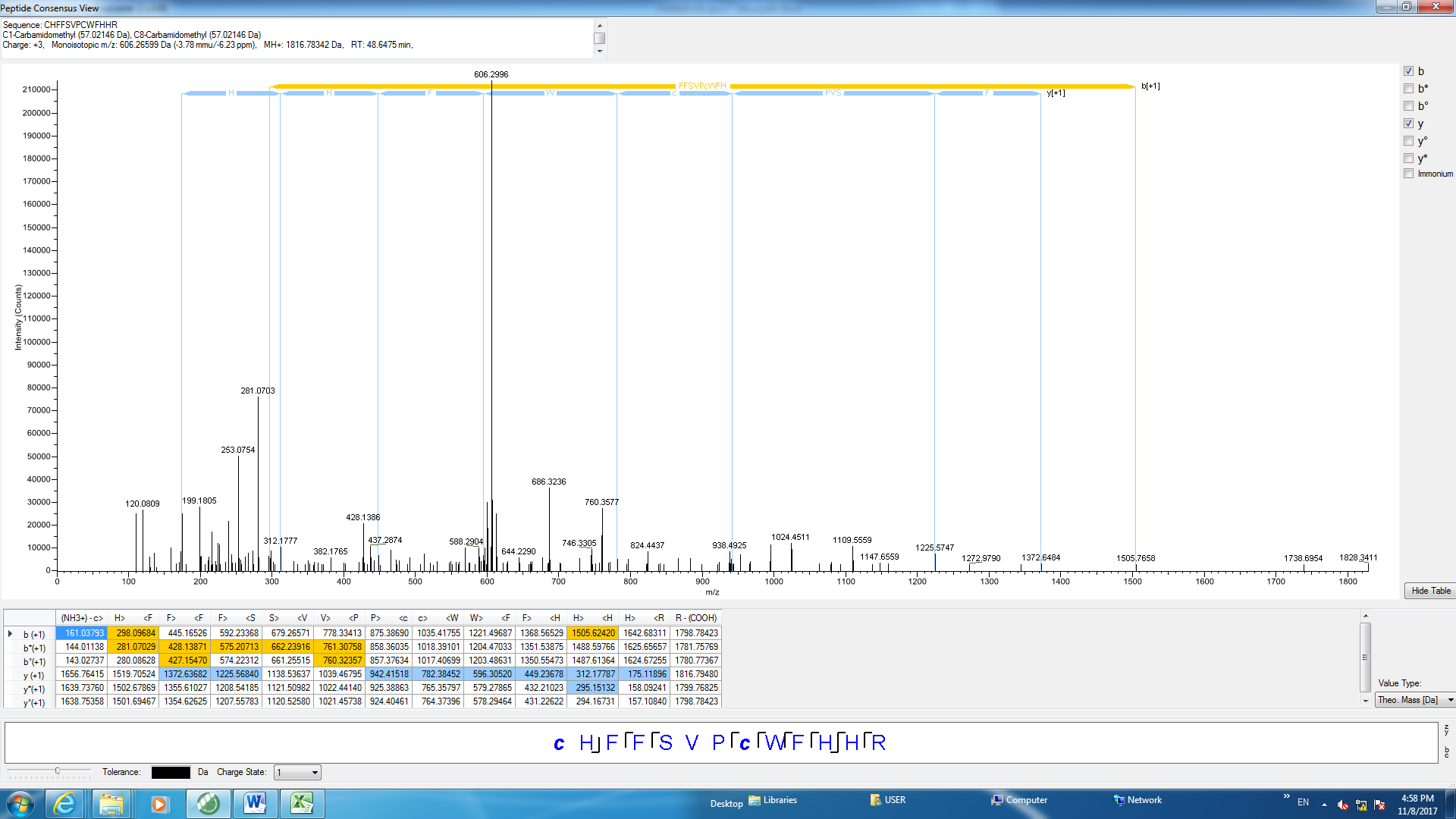


A8K7G6 highly similar to Homo sapiens regenerating islet-derived 1 alpha (1/3)


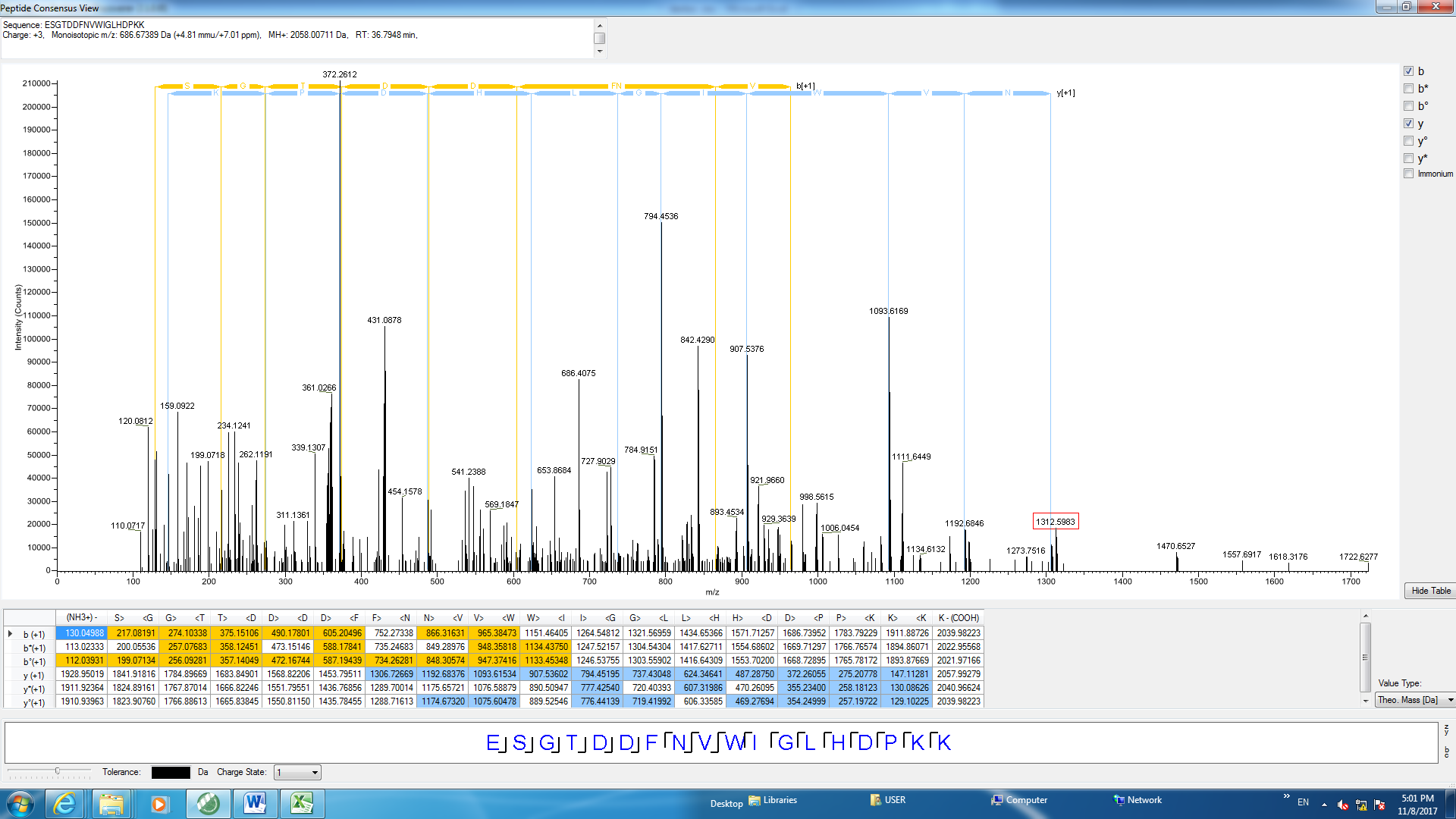


A8K7G6 highly similar to Homo sapiens regenerating islet-derived 1 alpha (2/3)


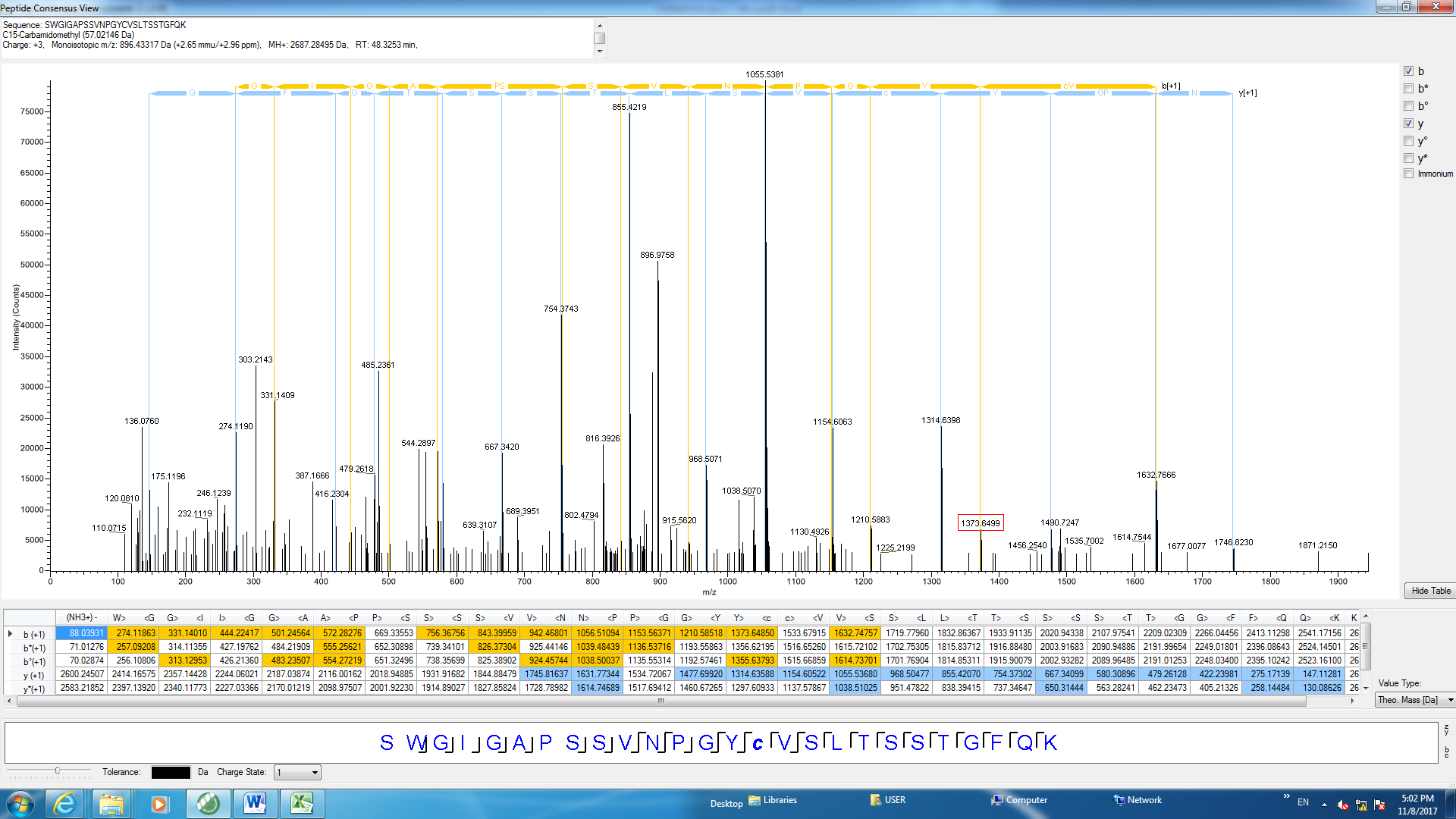


A8K7G6 highly similar to Homo sapiens regenerating islet-derived 1 alpha (3/3)


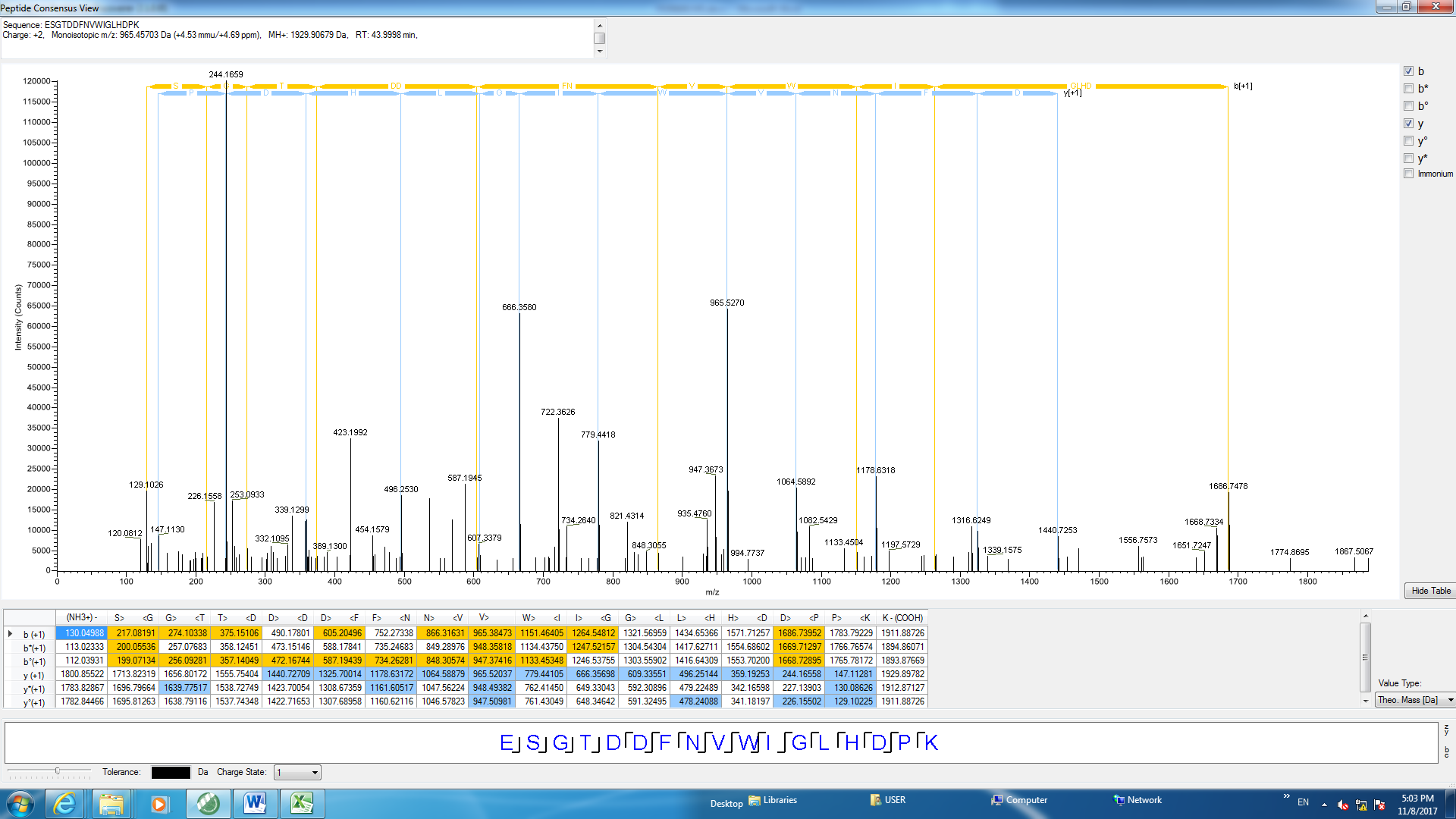


A0A1U9X8X6 CDSN (1/1)
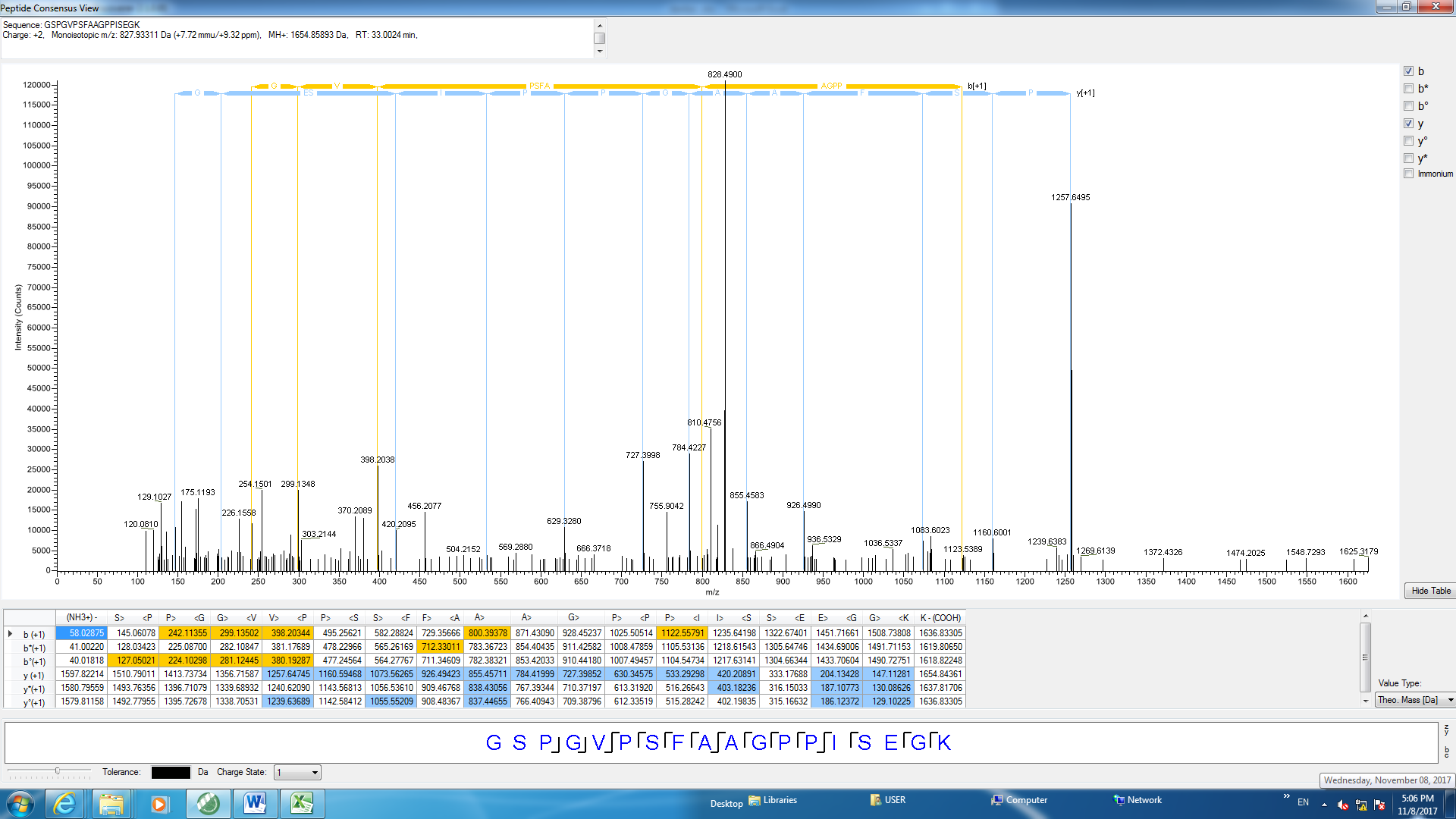


Q76LA1 CSTB protein (1/2)


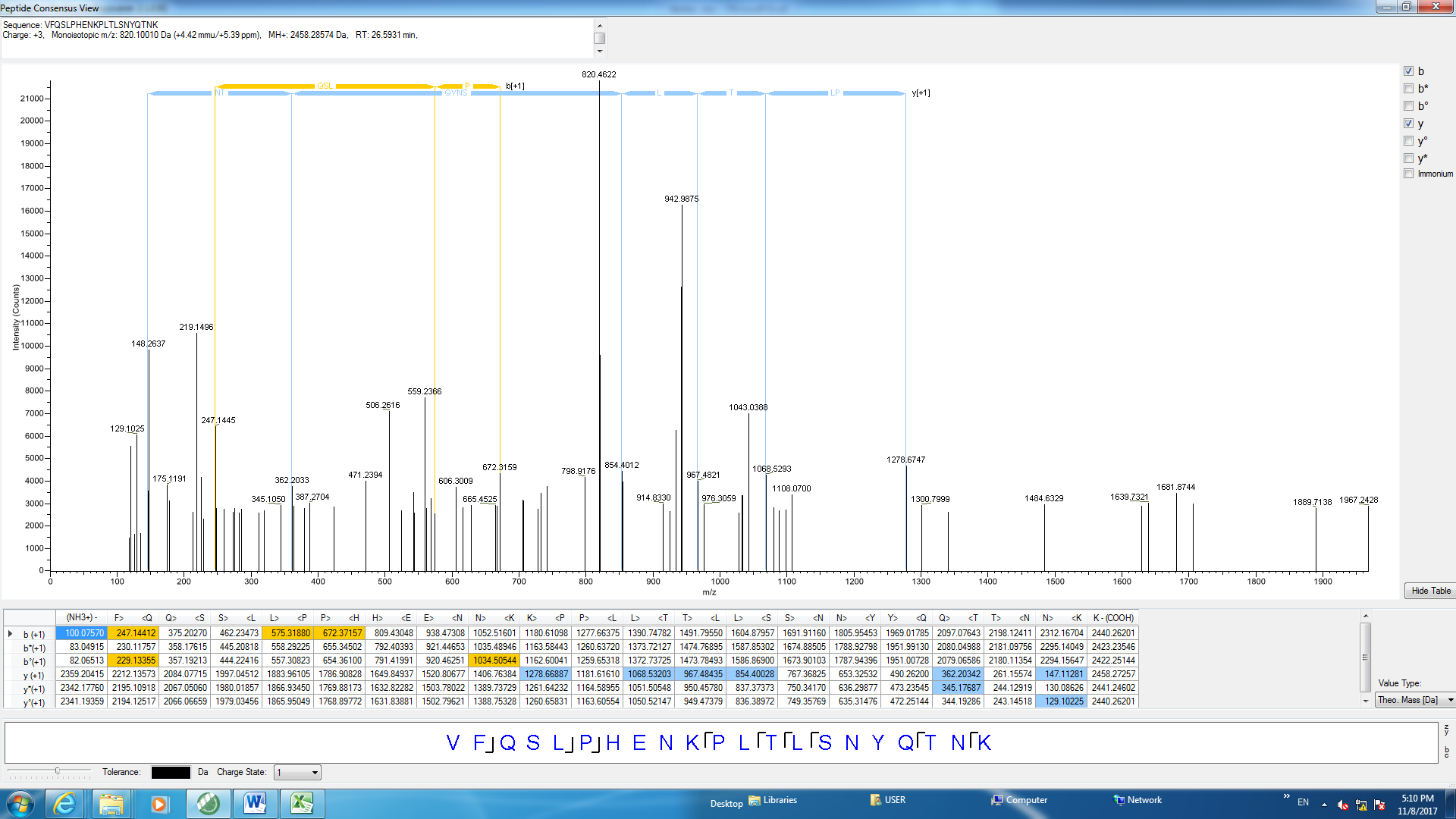


Q76LA1 CSTB protein (2/2)


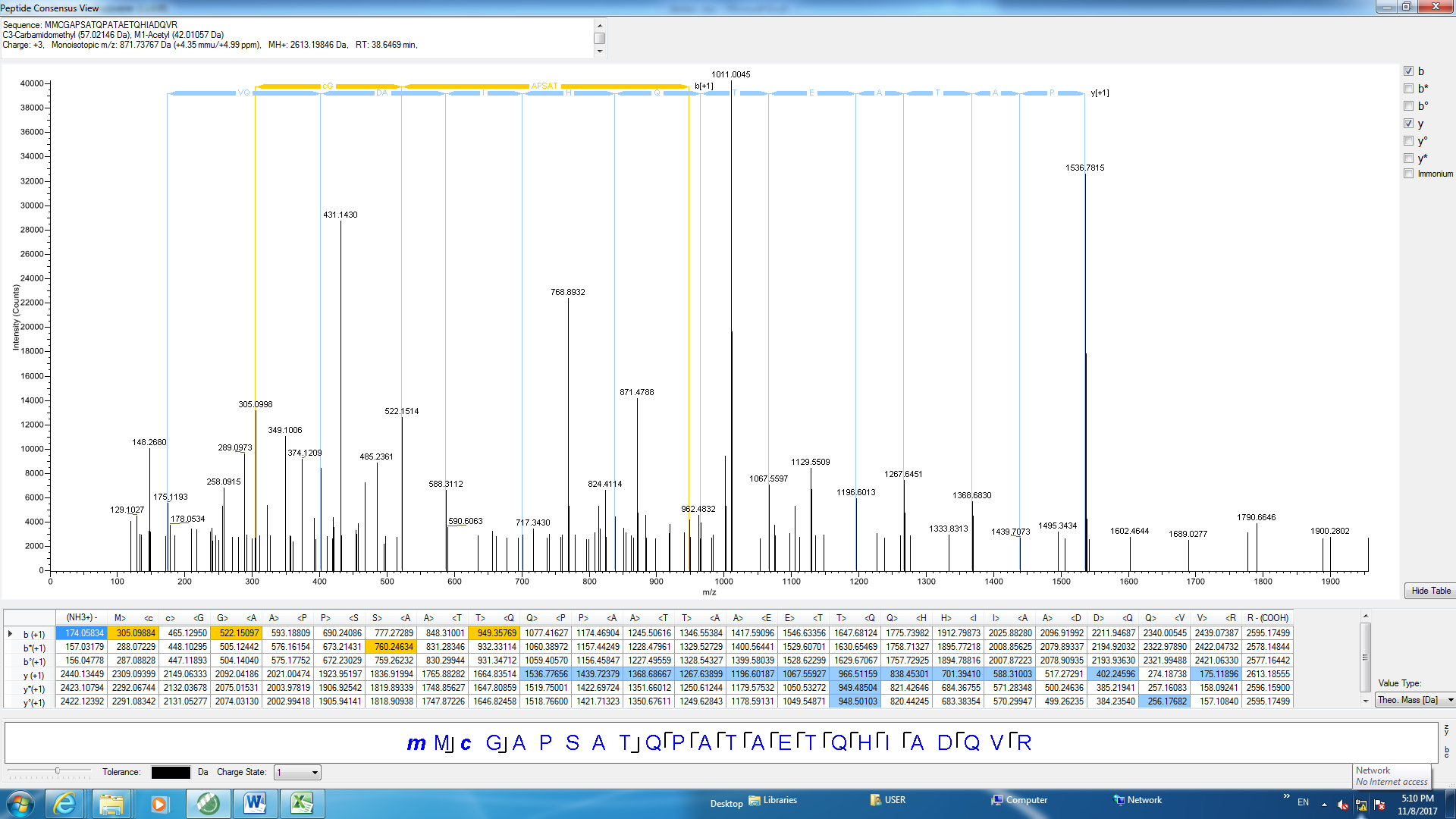


P01040 Cystatin-A (1/2)


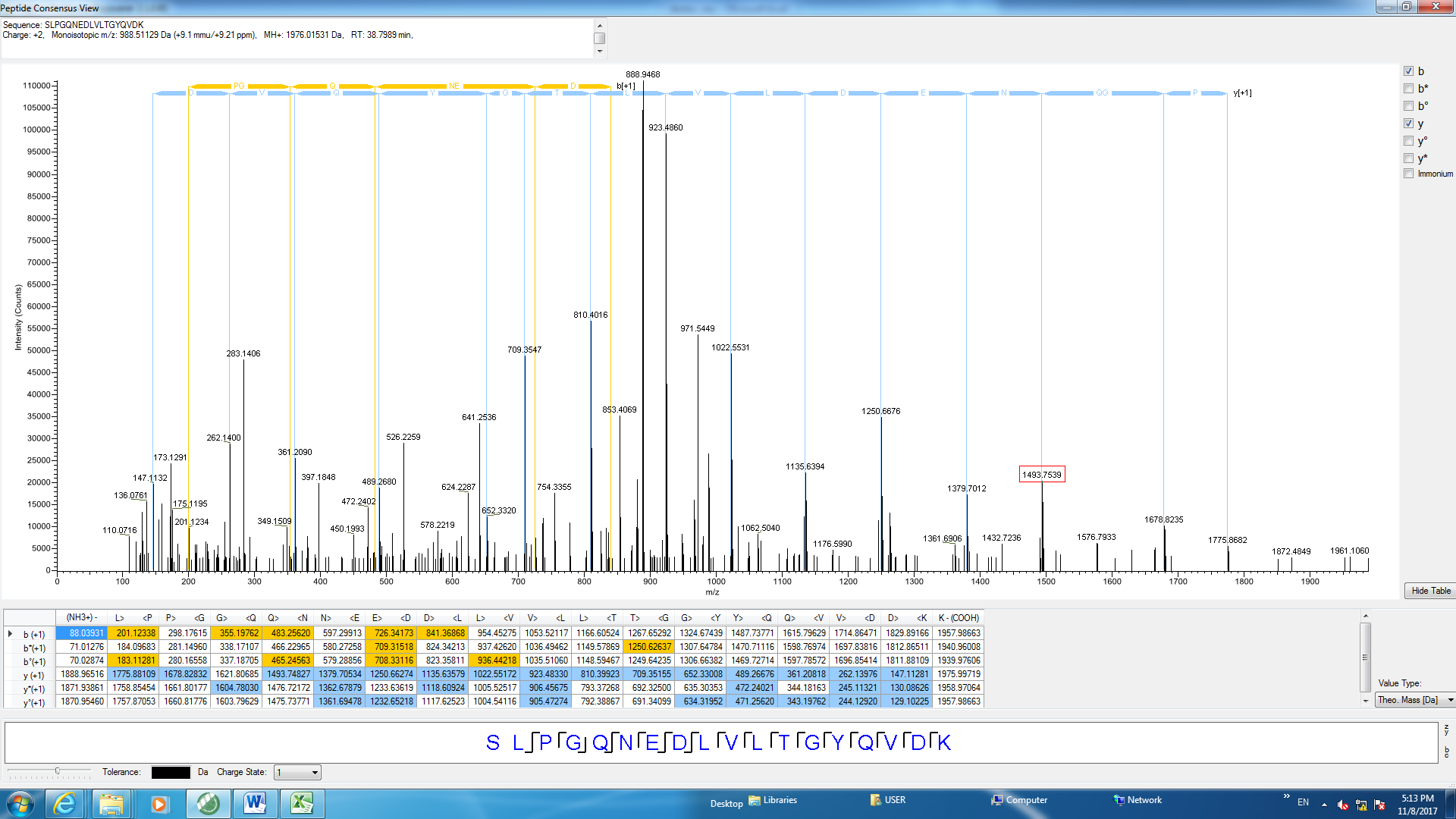


P01040 Cystatin-A (2/2)


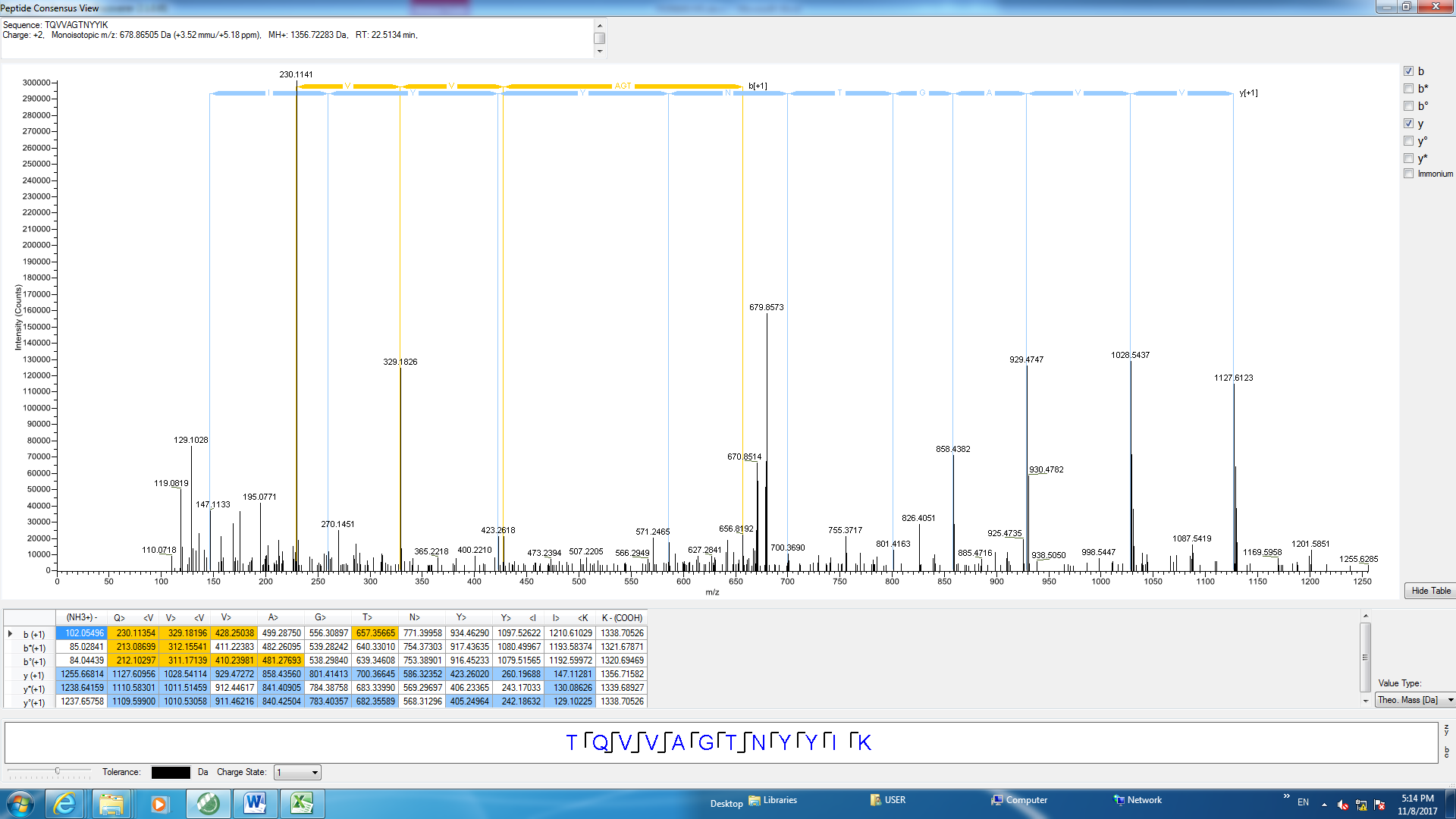


P01036 Cystatin-S (1/1)


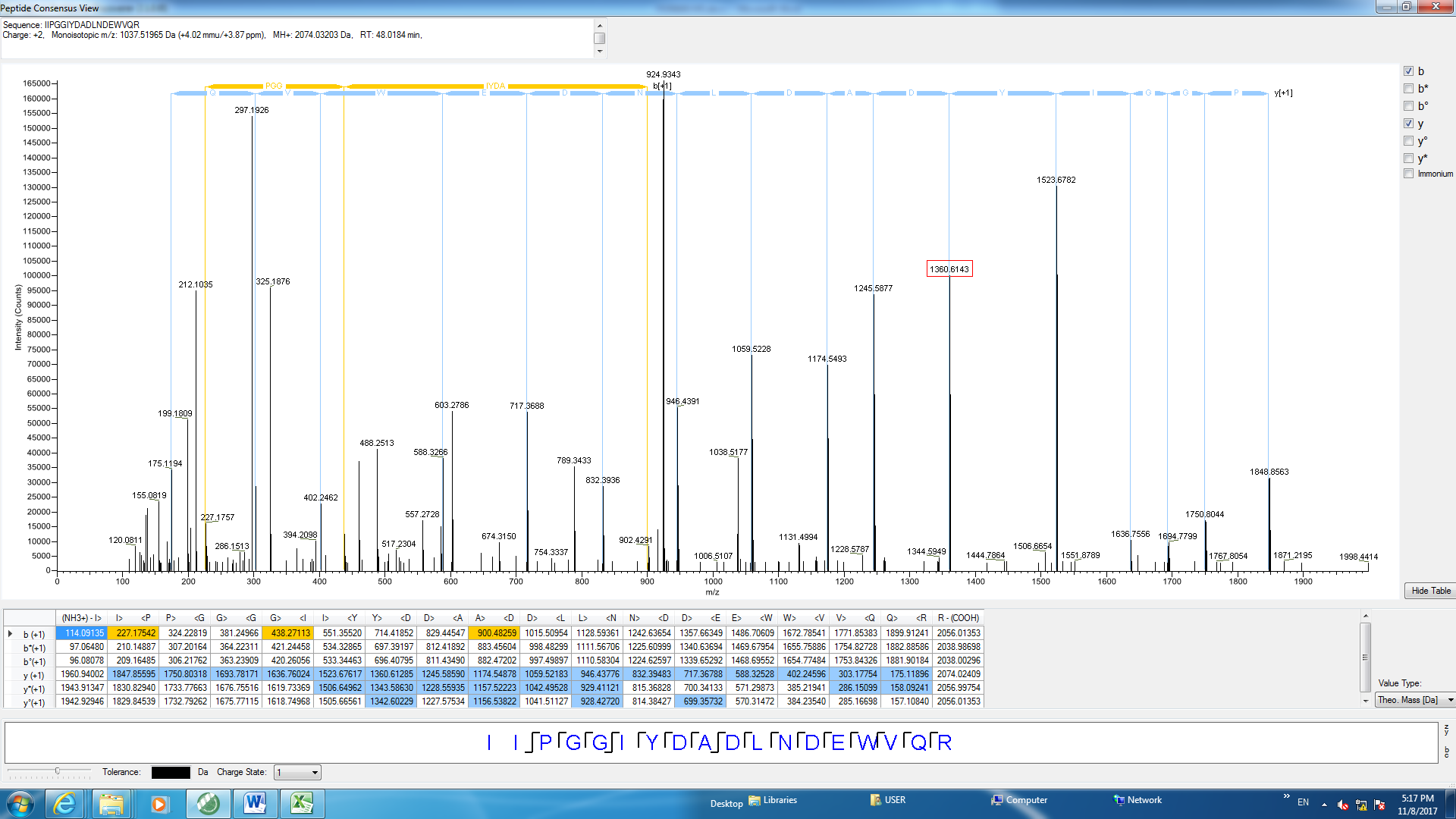


P01037 Cystatin-SN (1/5)


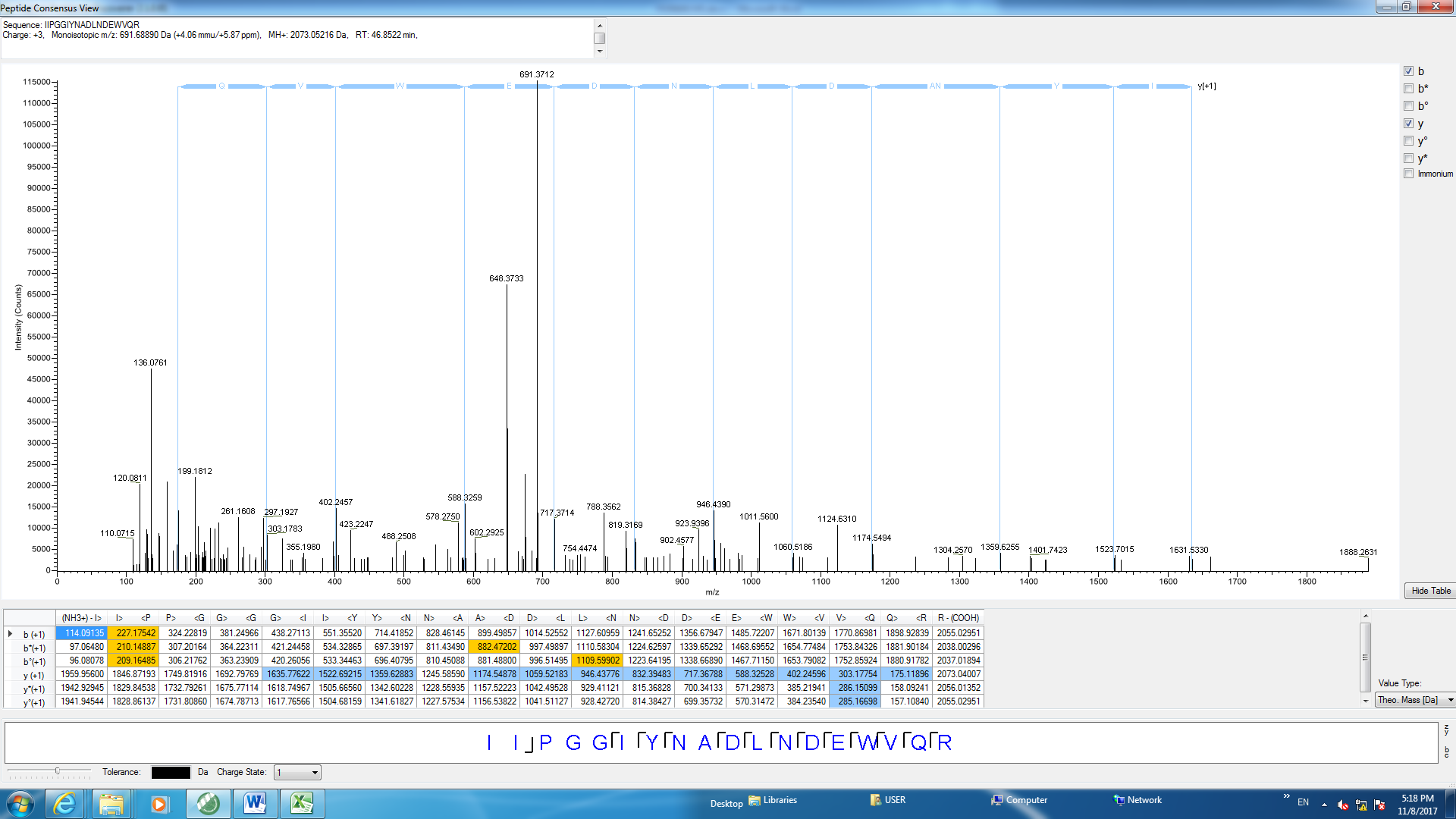


P01037 Cystatin-SN (2/5)


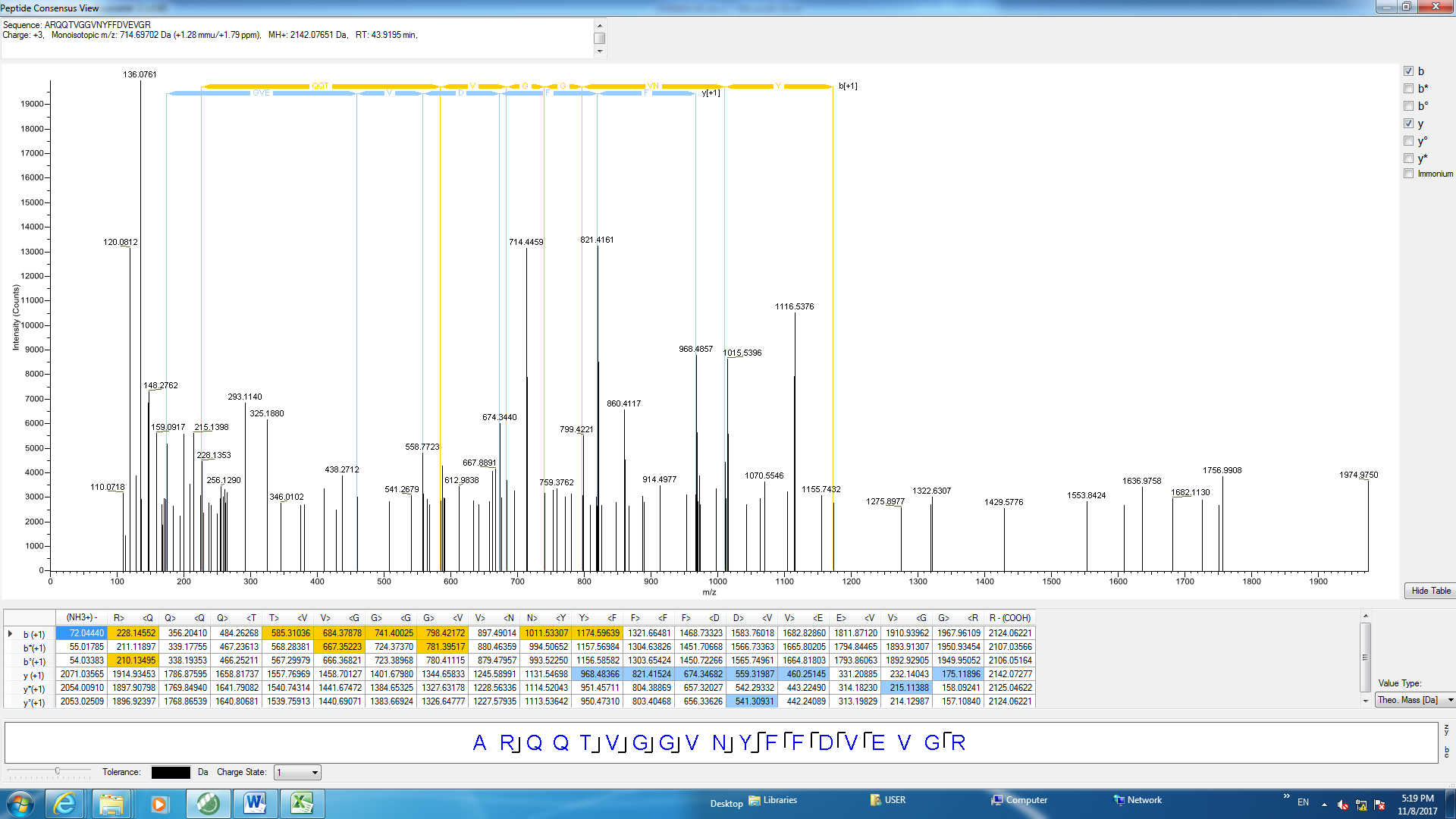


P01037 Cystatin-SN (3/5)


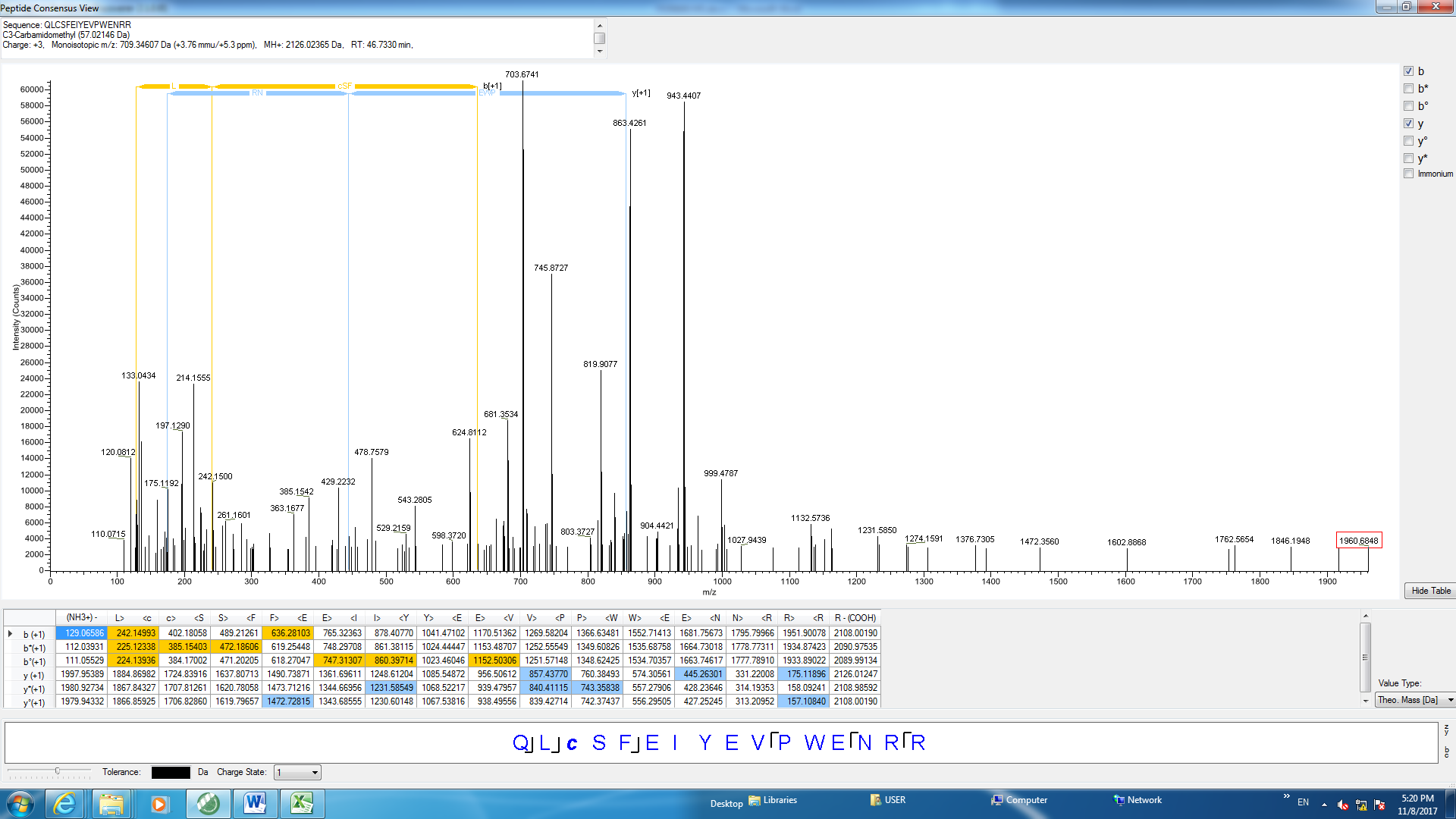


P01037 Cystatin-SN (4/5)


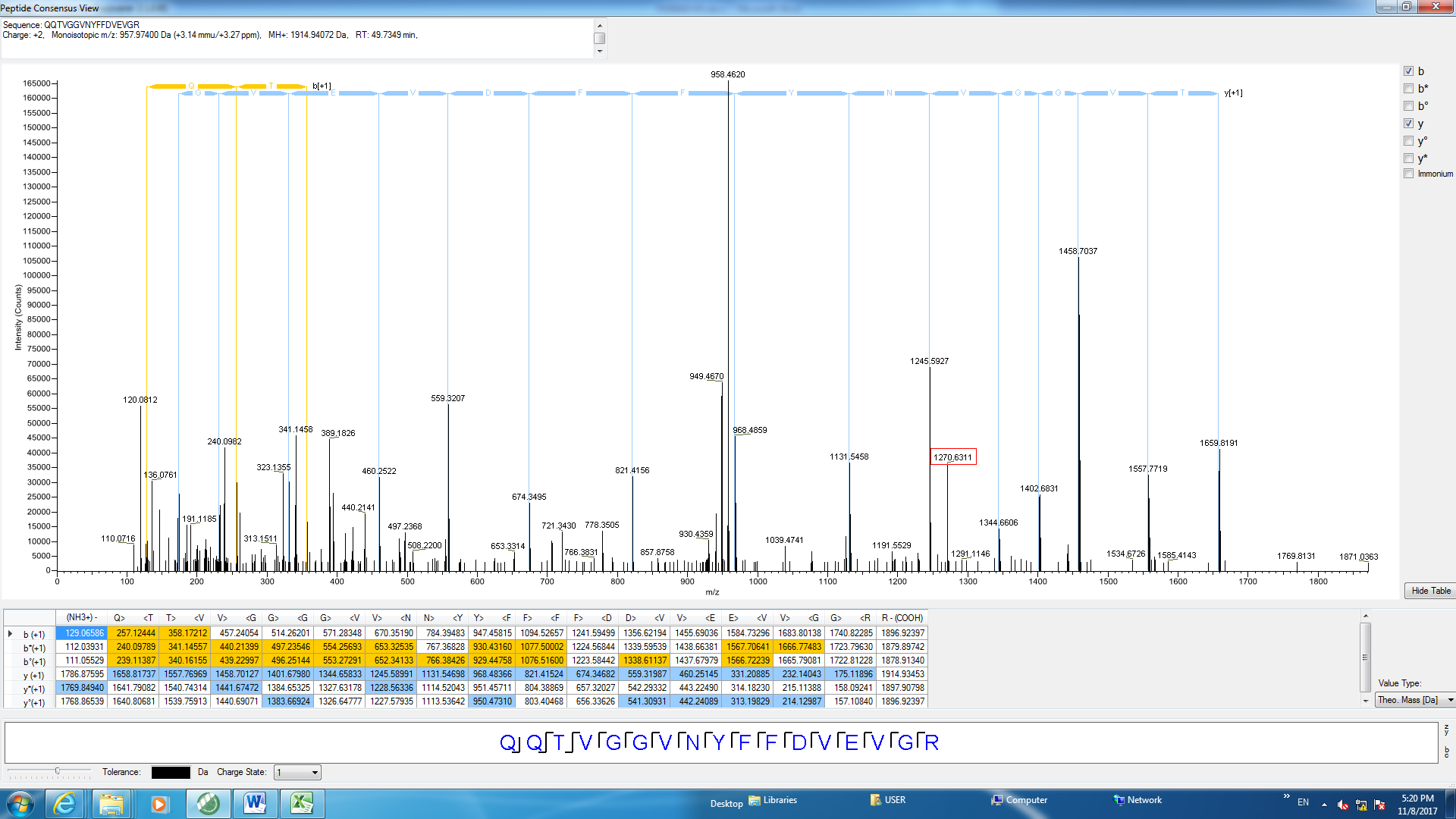


P01037 Cystatin-SN (5/5)


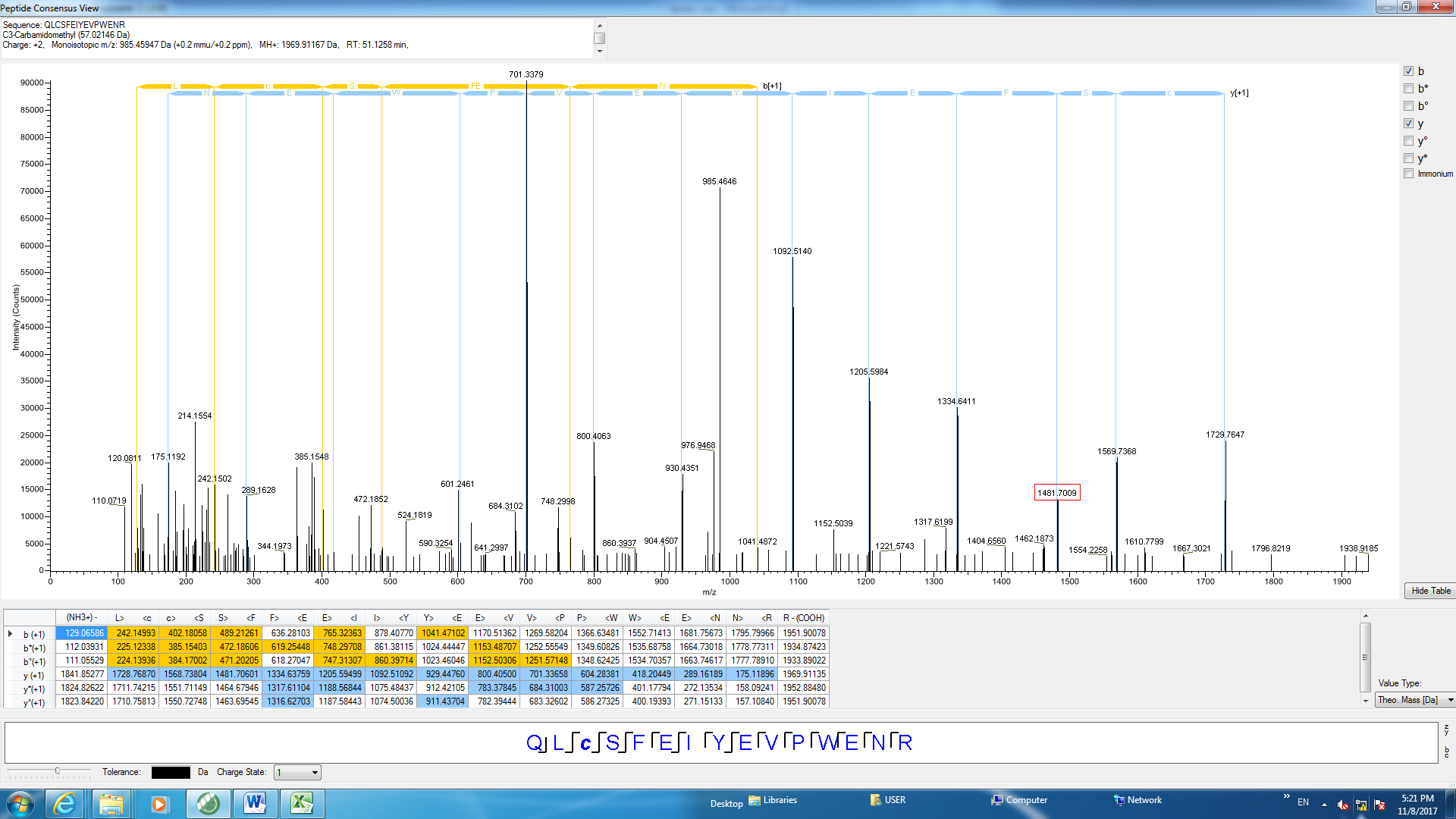


P99999 Cytochrome C (1/4)


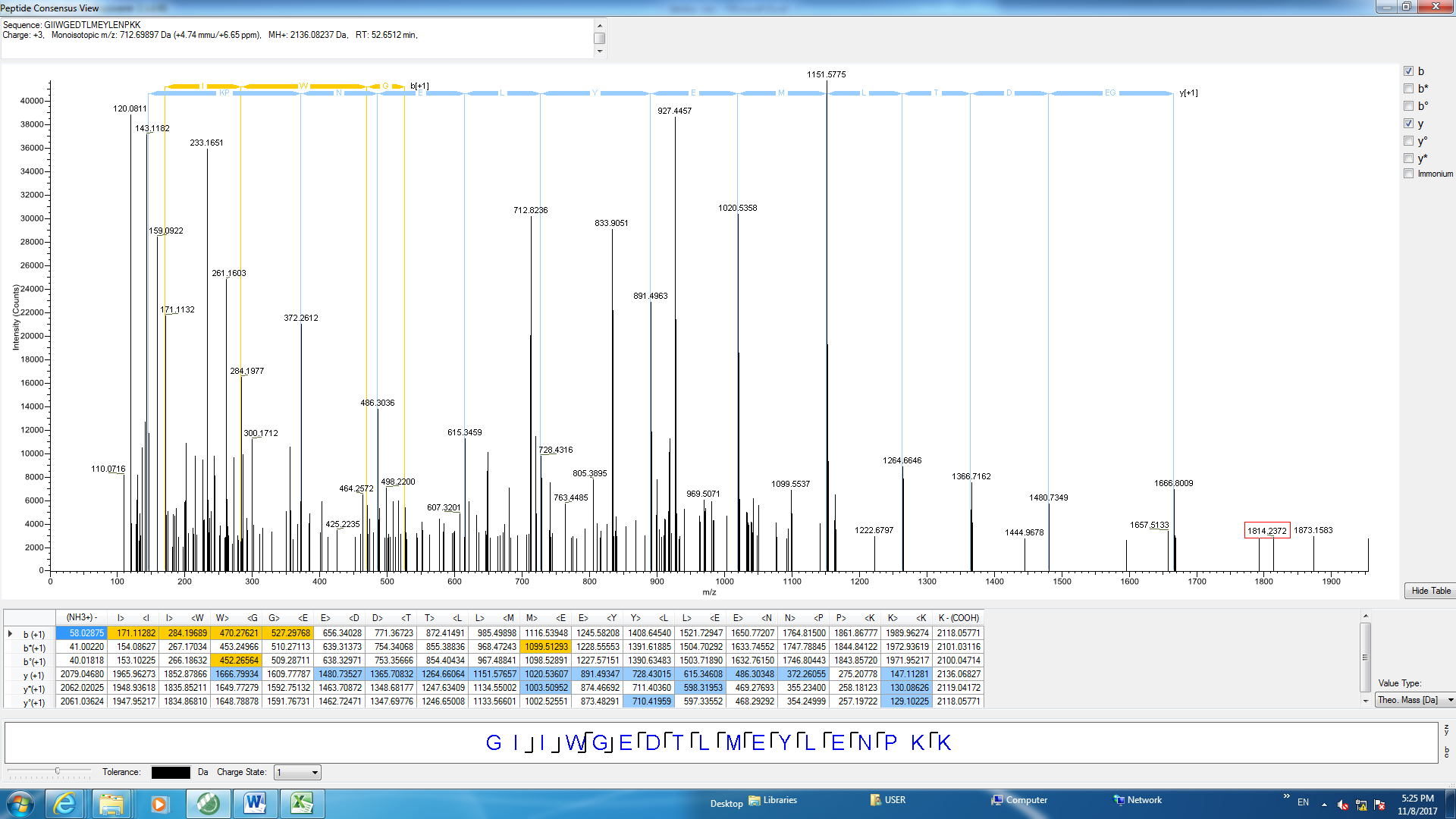


P99999 Cytochrome C (2/4)


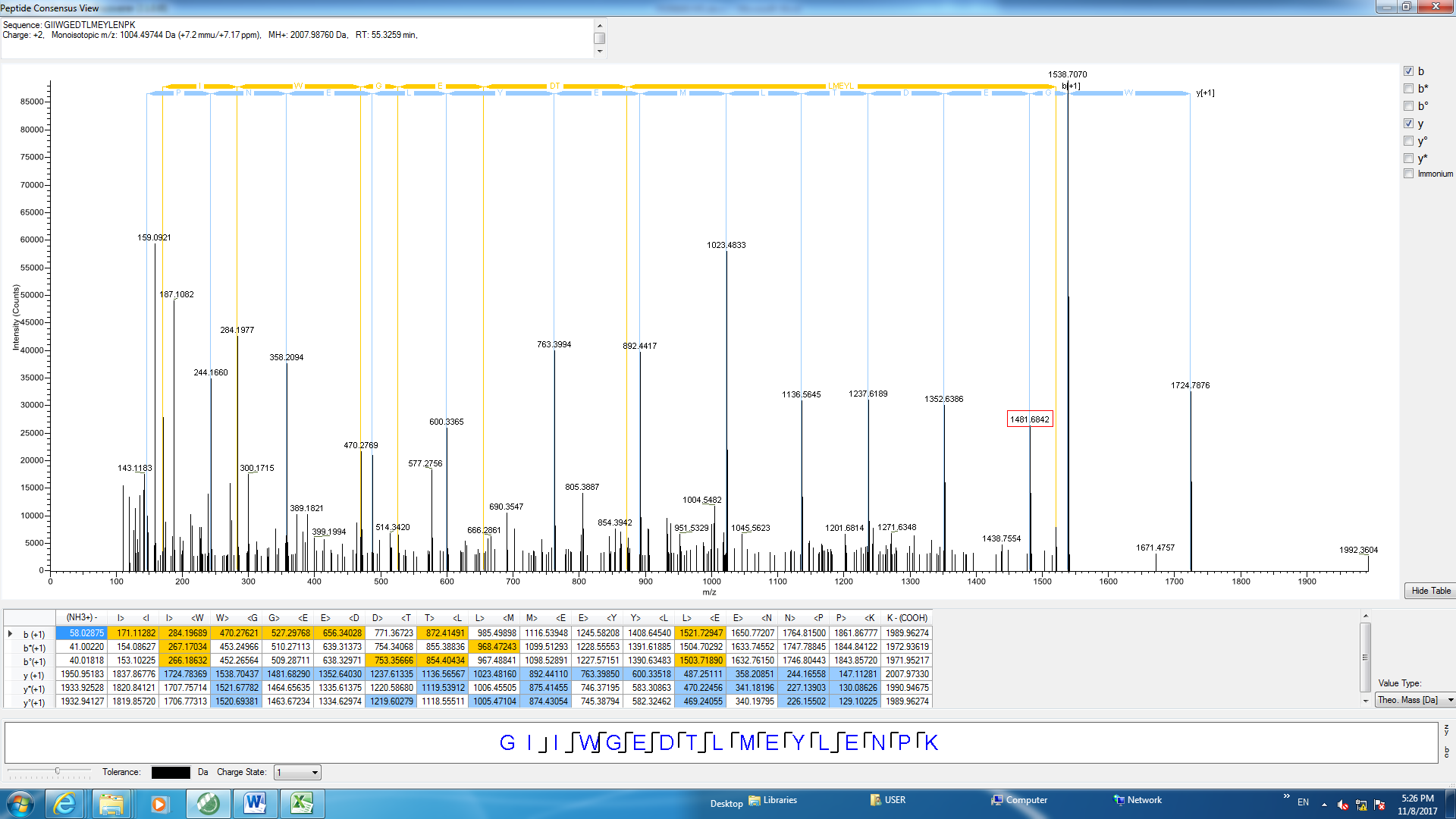


P99999 Cytochrome C (3/4)


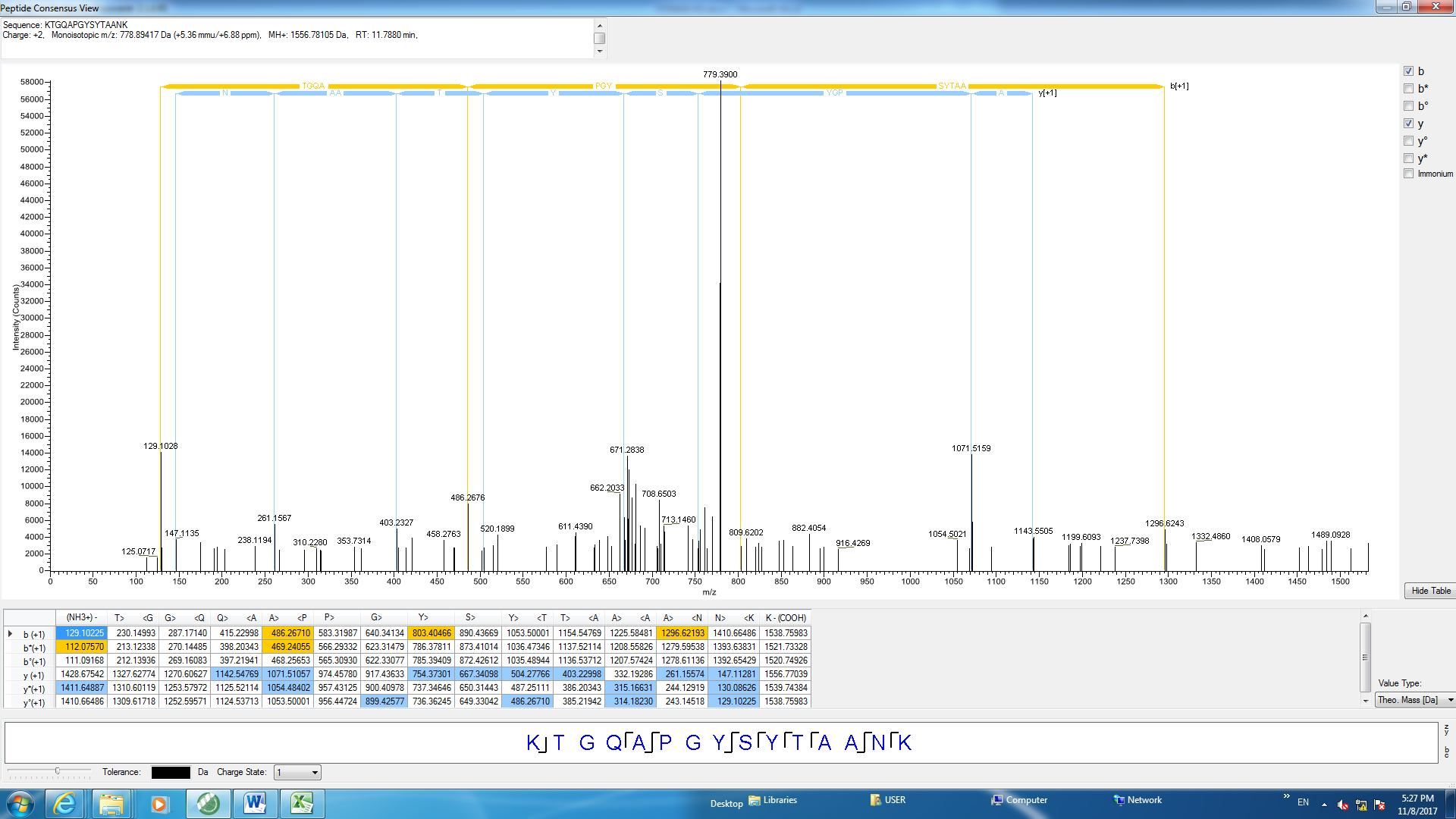


P99999 Cytochrome C (4/4)


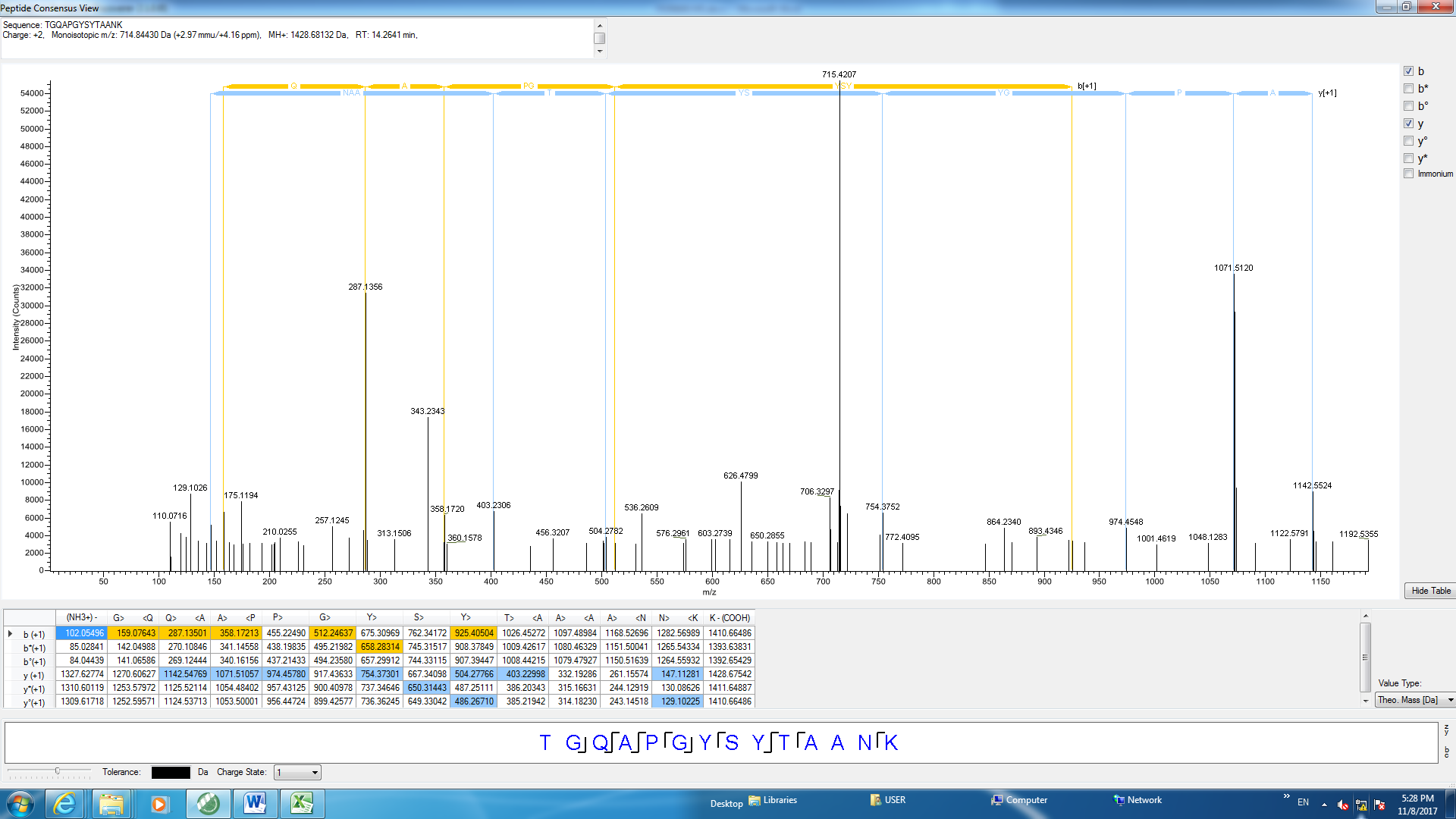


Q8TCX0 Delta 2-isopentenyl pyrophosphate transferase-like protein (Fragment) (1/1)


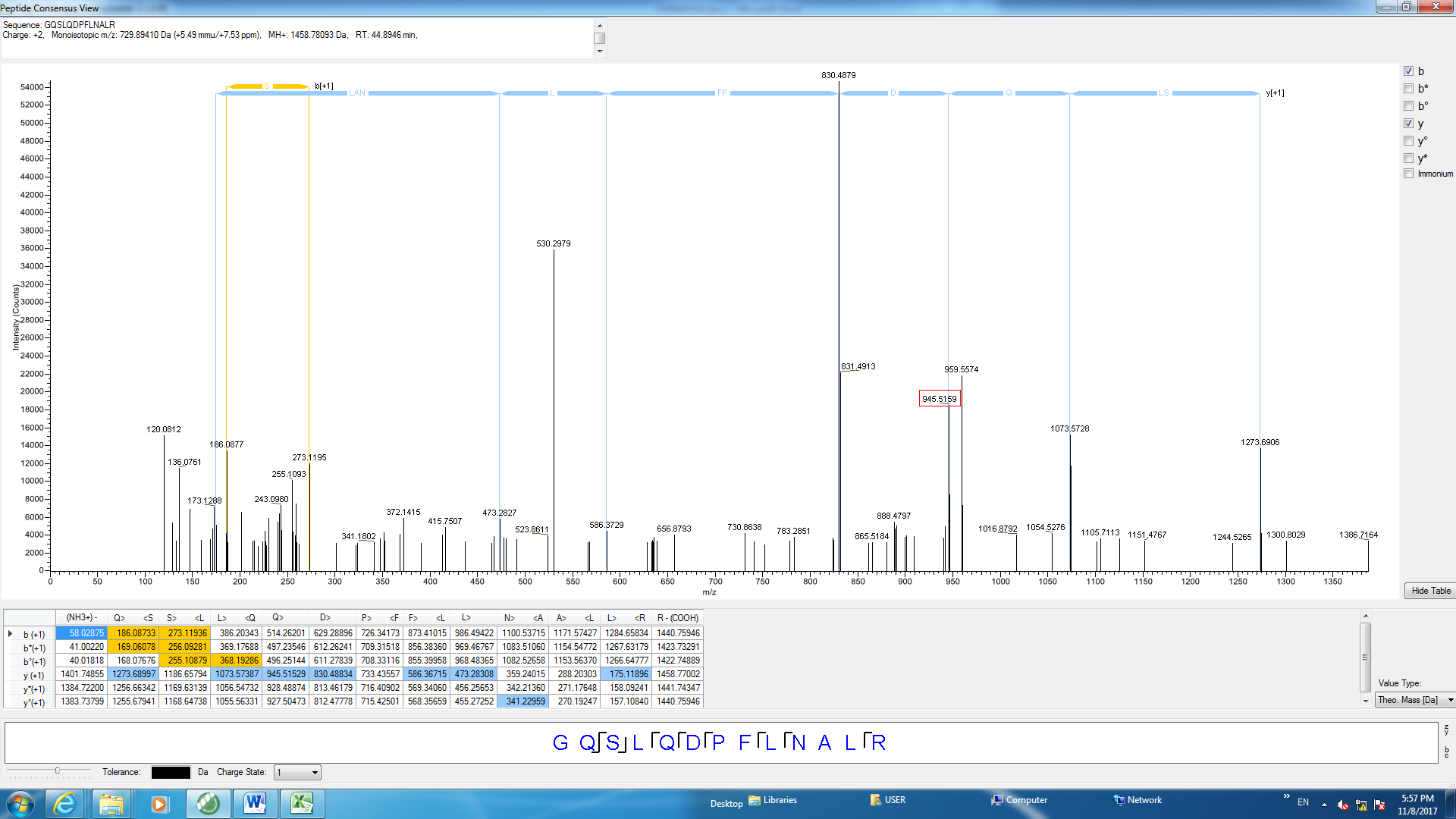


P36957 Dihydrolipoyllysine-residue succinyltransferase component of 2-oxoglutarate dehydrogenase complex, mitochondrial (1/1)


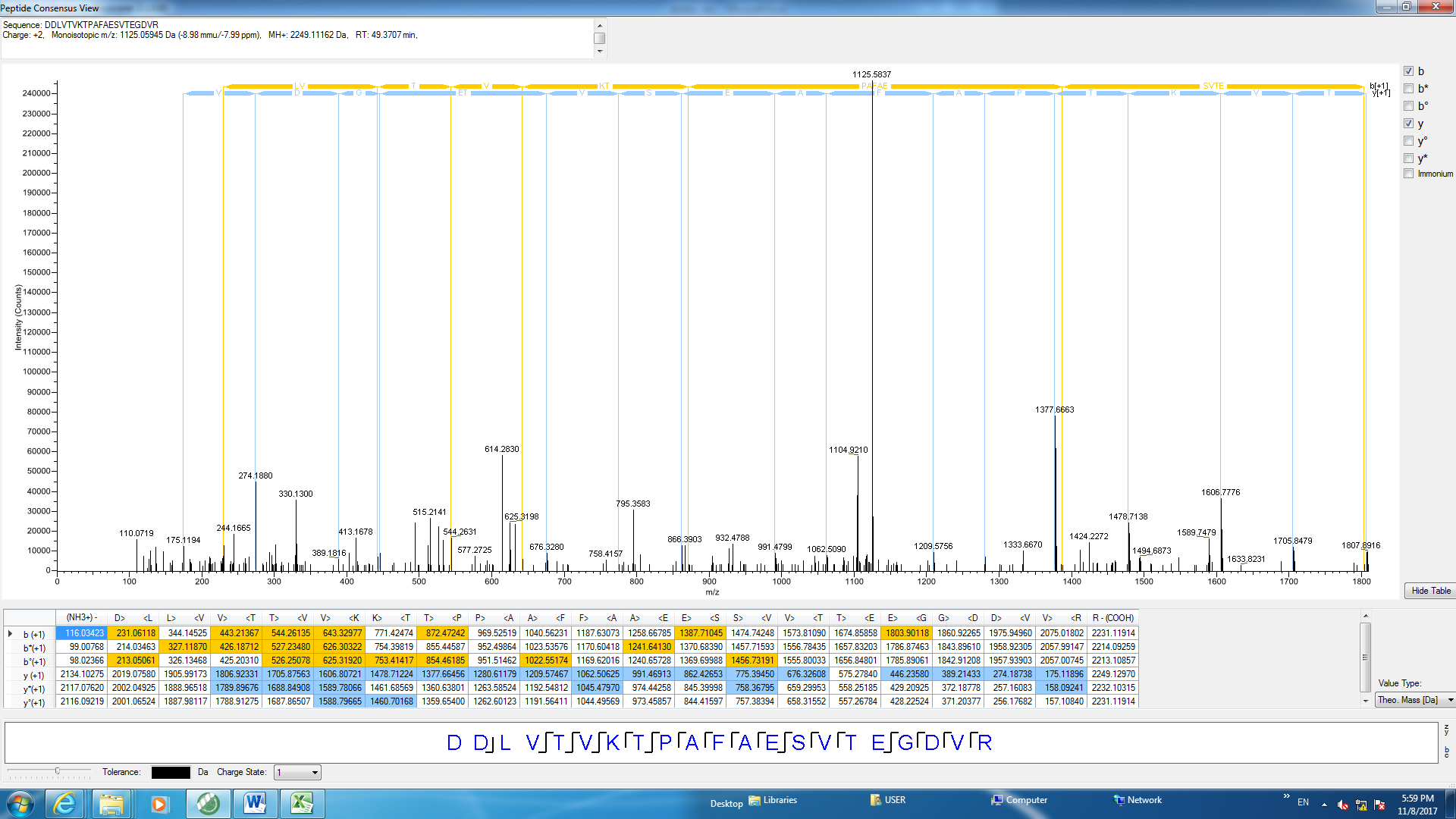


V9HW80 Epididymis luminal protein 220 (1/1)


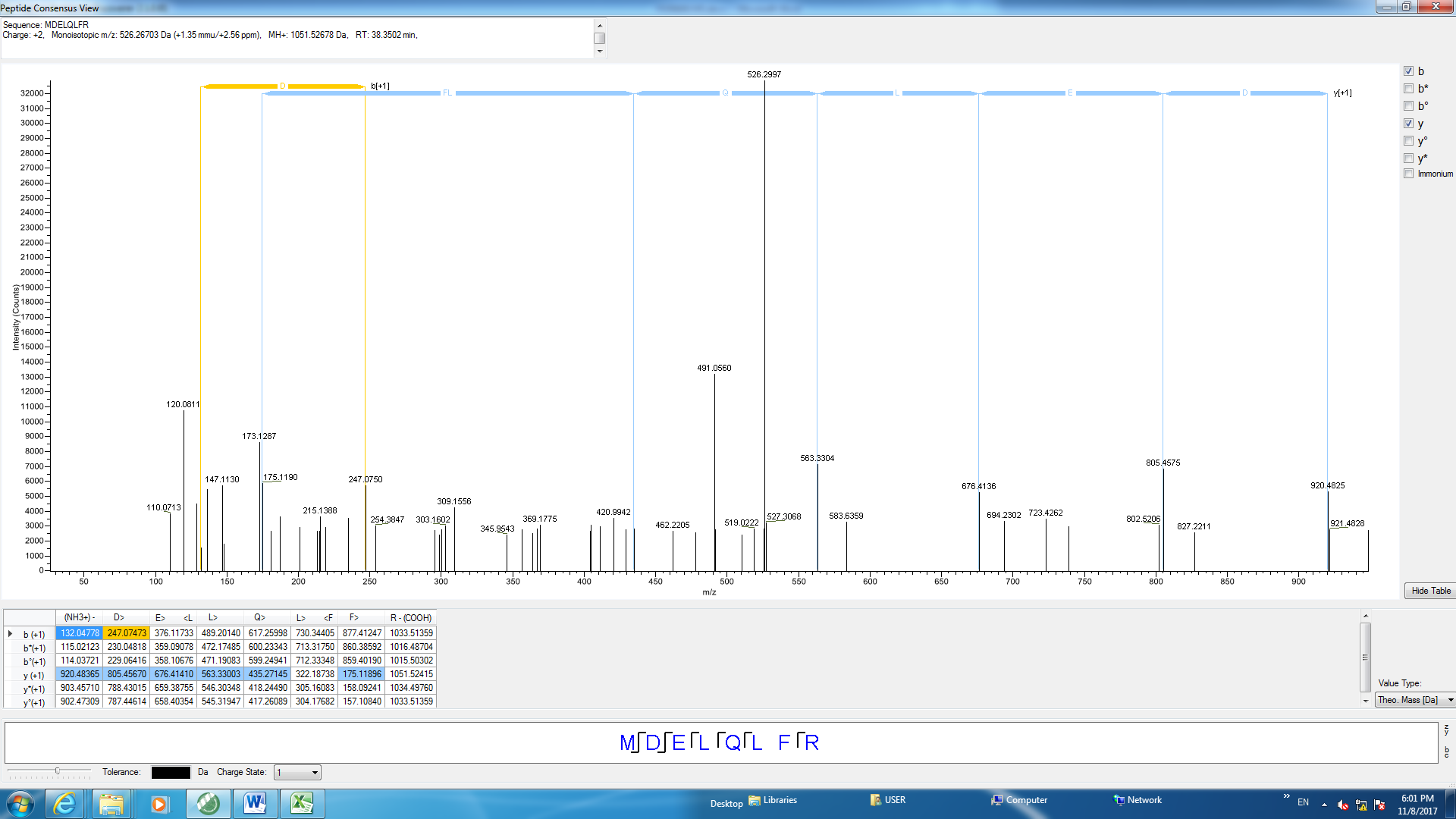


V9HWA9 Epididymis secretory sperm binding protein Li 62p (1/2)


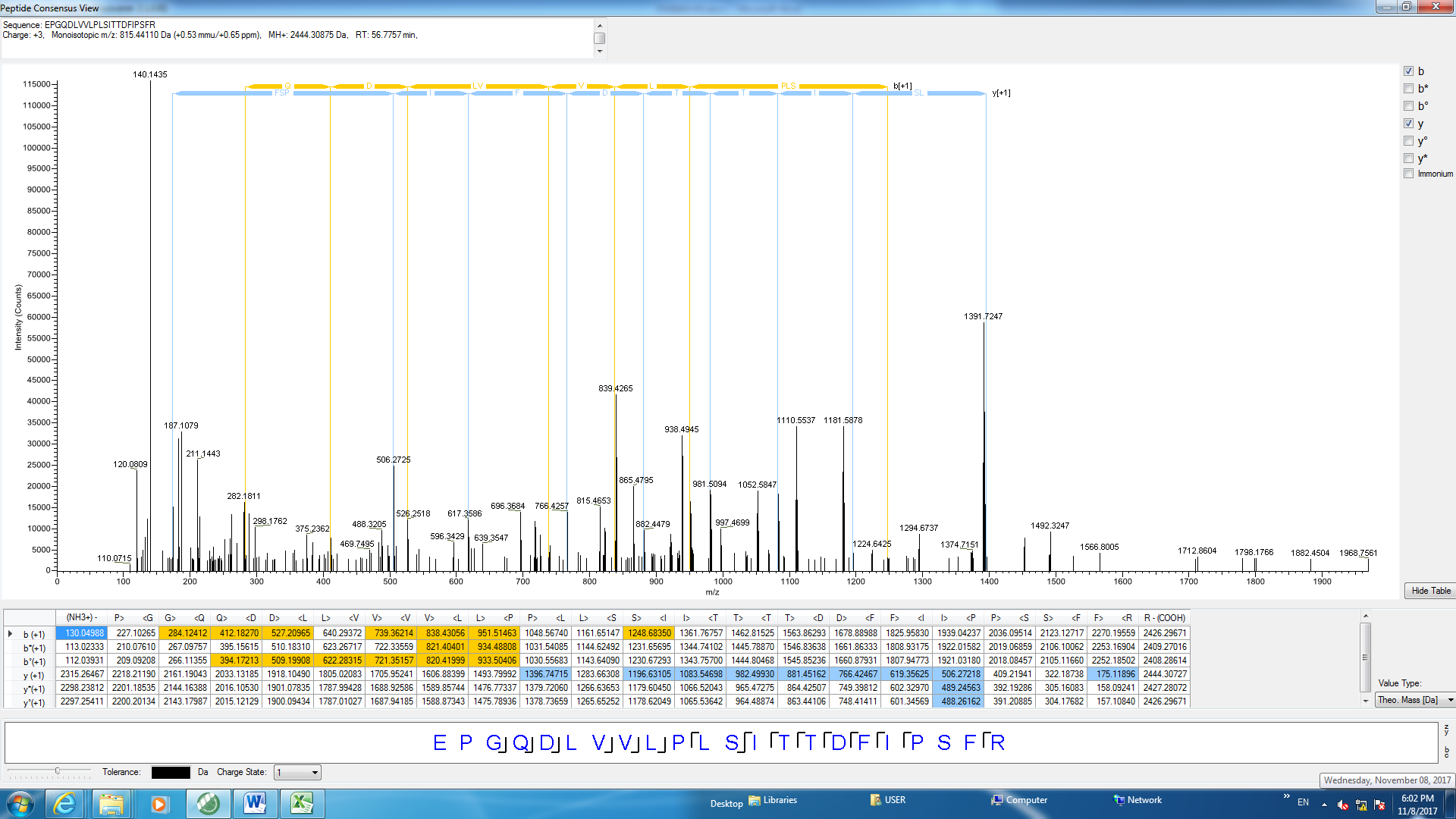


V9HWA9 Epididymis secretory sperm binding protein Li 62p (2/2)


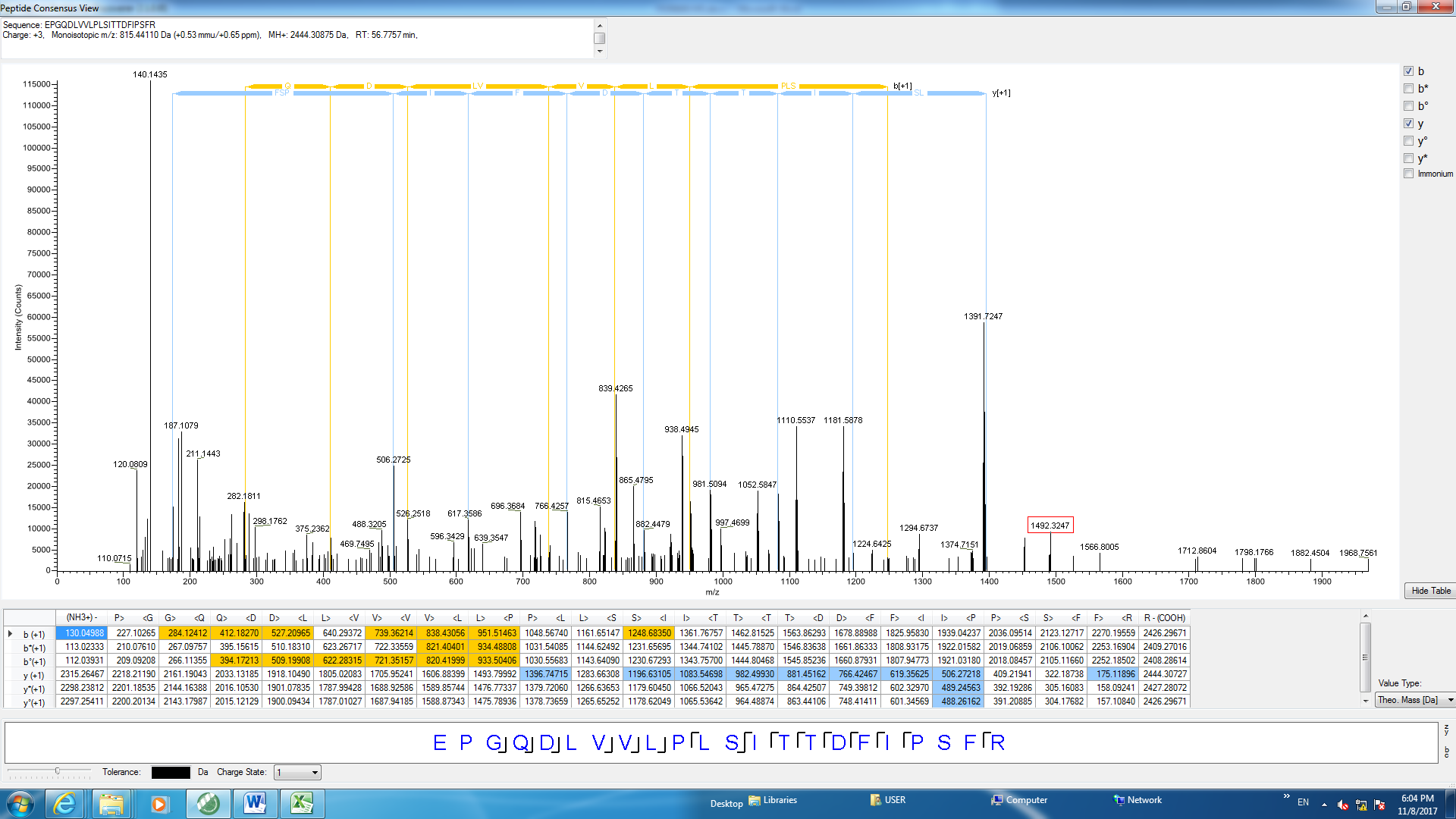


B7Z3K9 Fructose-bisphosphate aldolase (1/1)


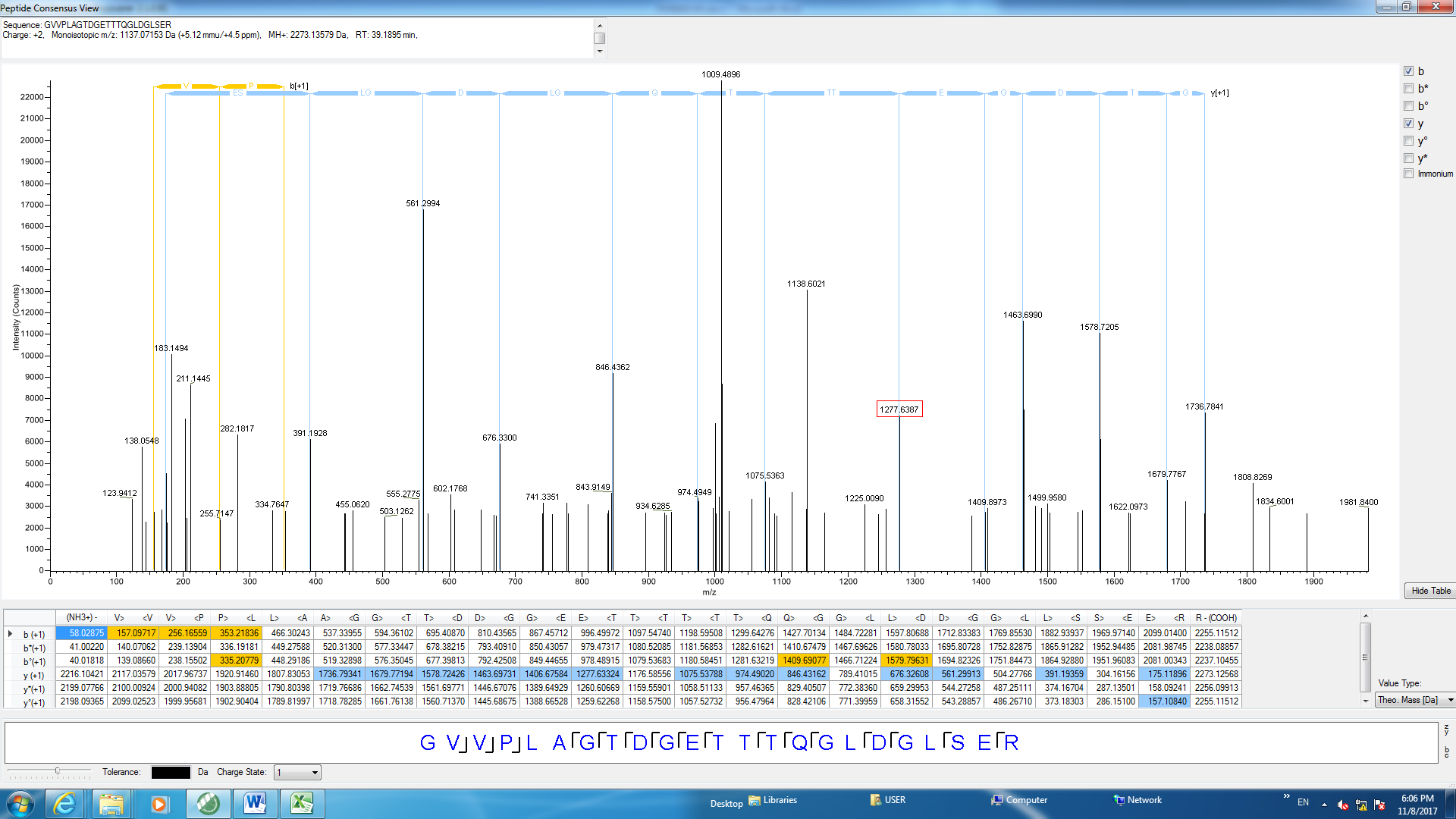


A0A1K0GXZ1 Globin C1 (1/2)


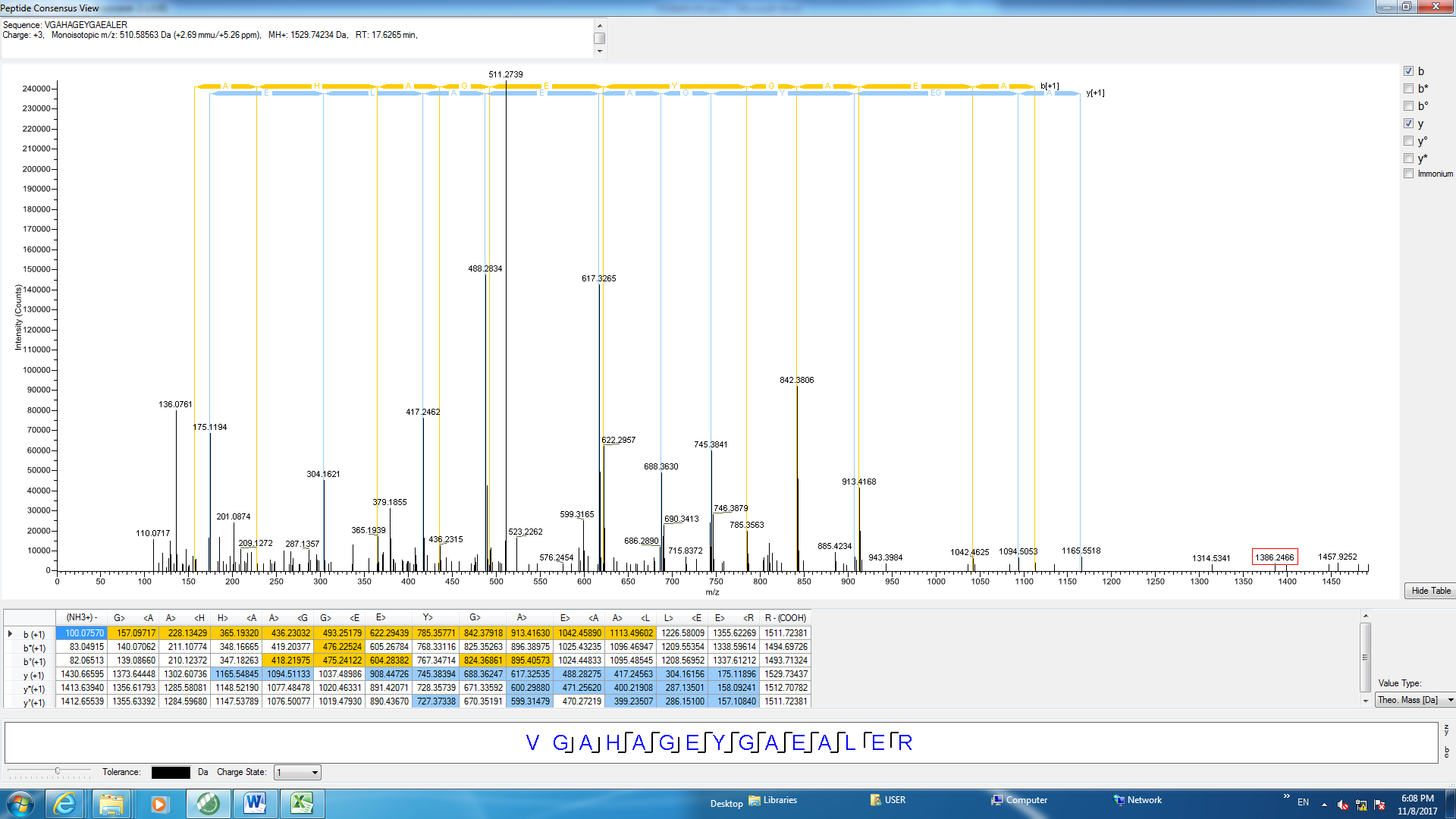


A0A1K0GXZ1 Globin C1 (2/2)


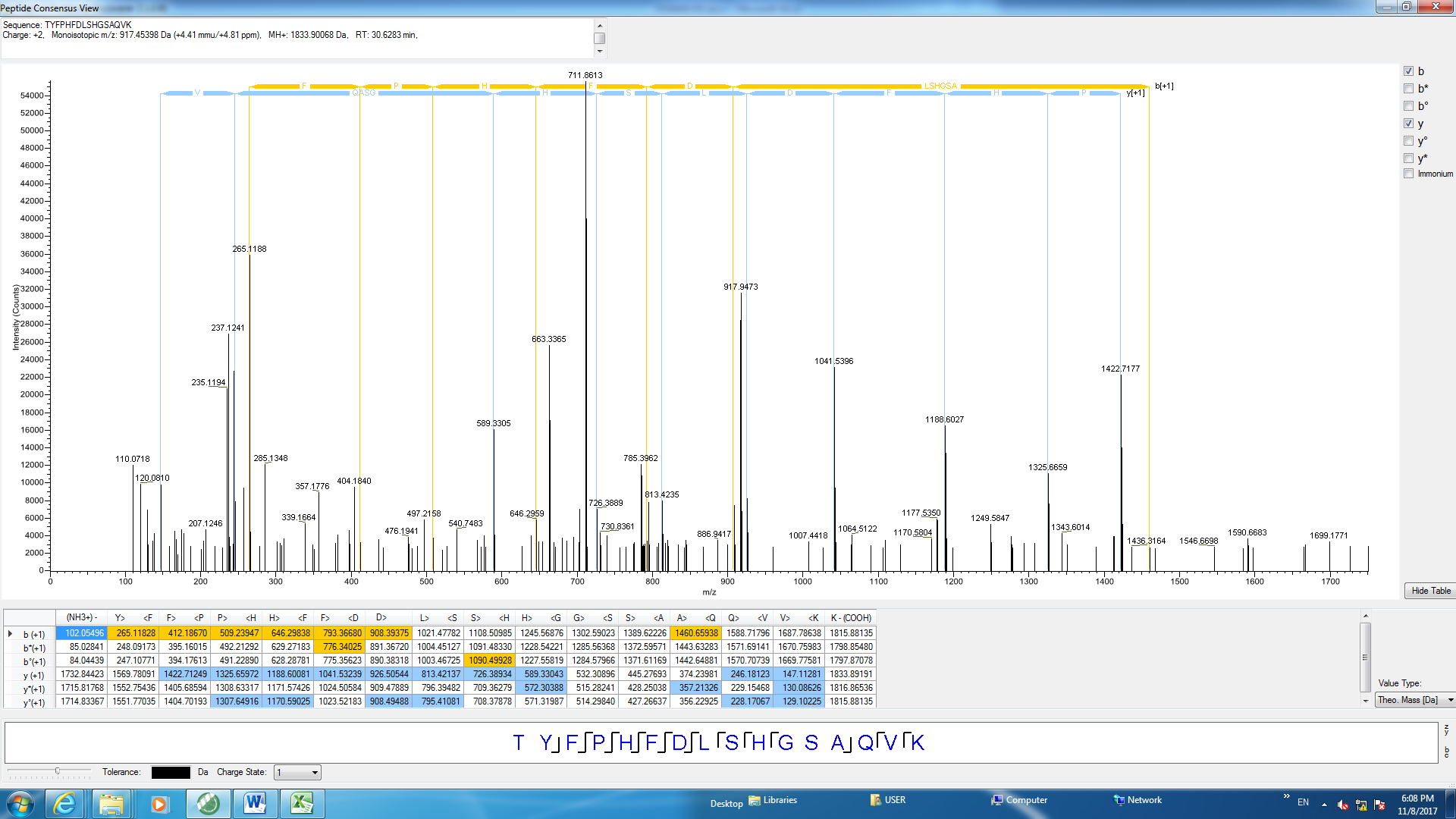


A0A024RAM2 Glutaredoxin (Thioltransferase), isoform CRA_c (1/1)


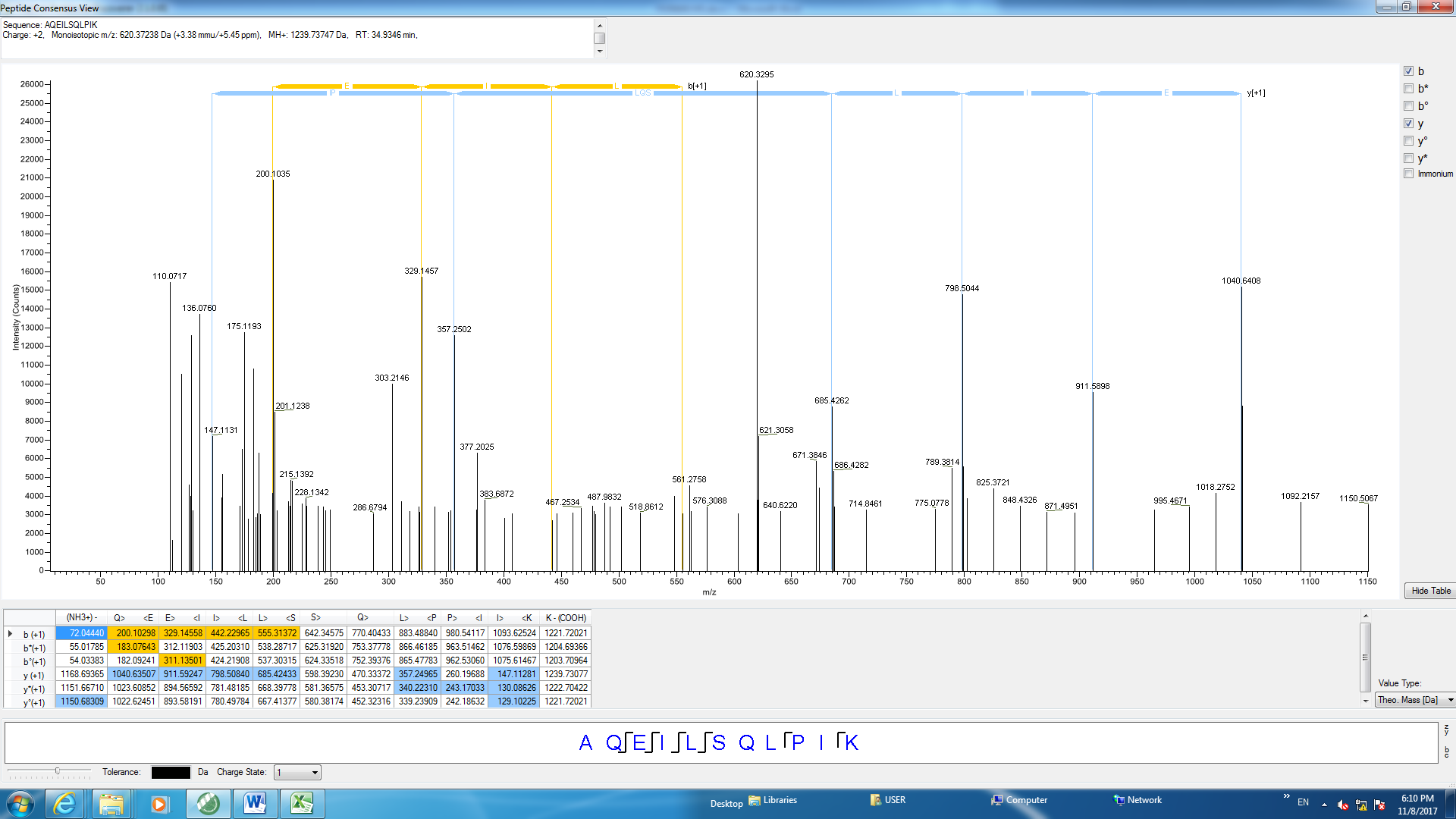


P04406 Glyceraldehyde-3-phosphate dehydrogenase (1/1)


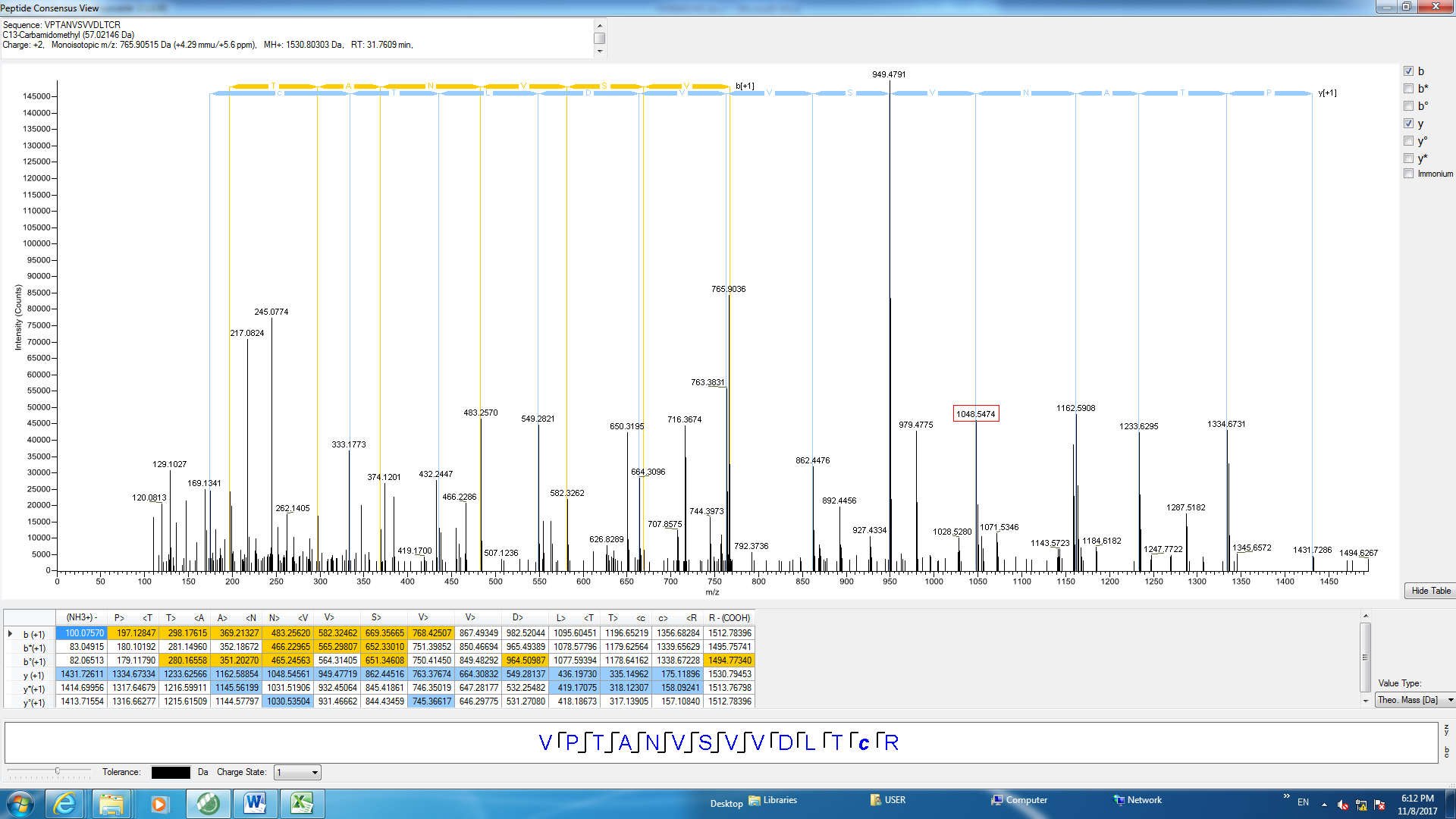


Q99988 Growth/differentiation factor 15 (1/2)


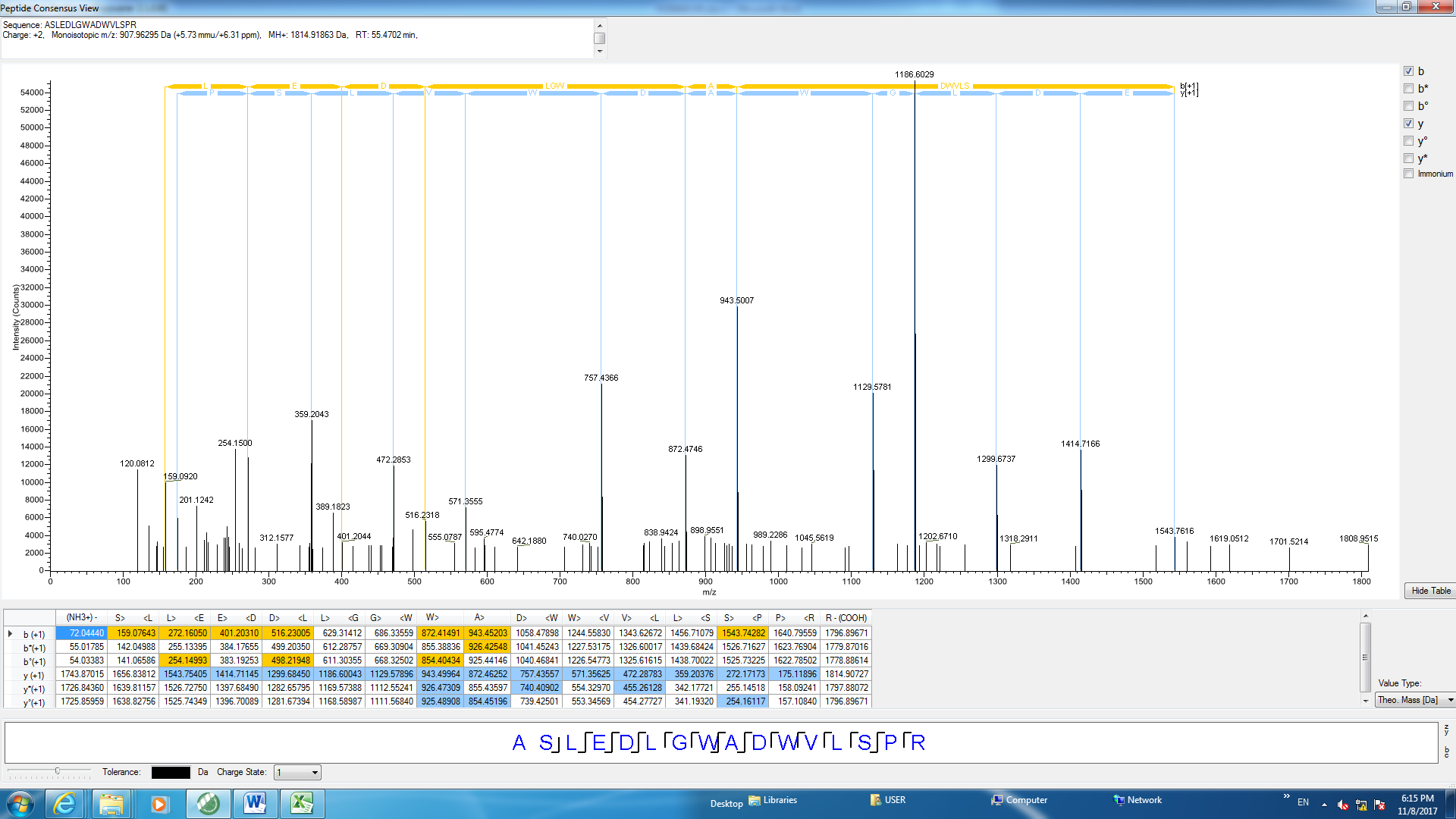


Q99988 Growth/differentiation factor 15 (2/2)


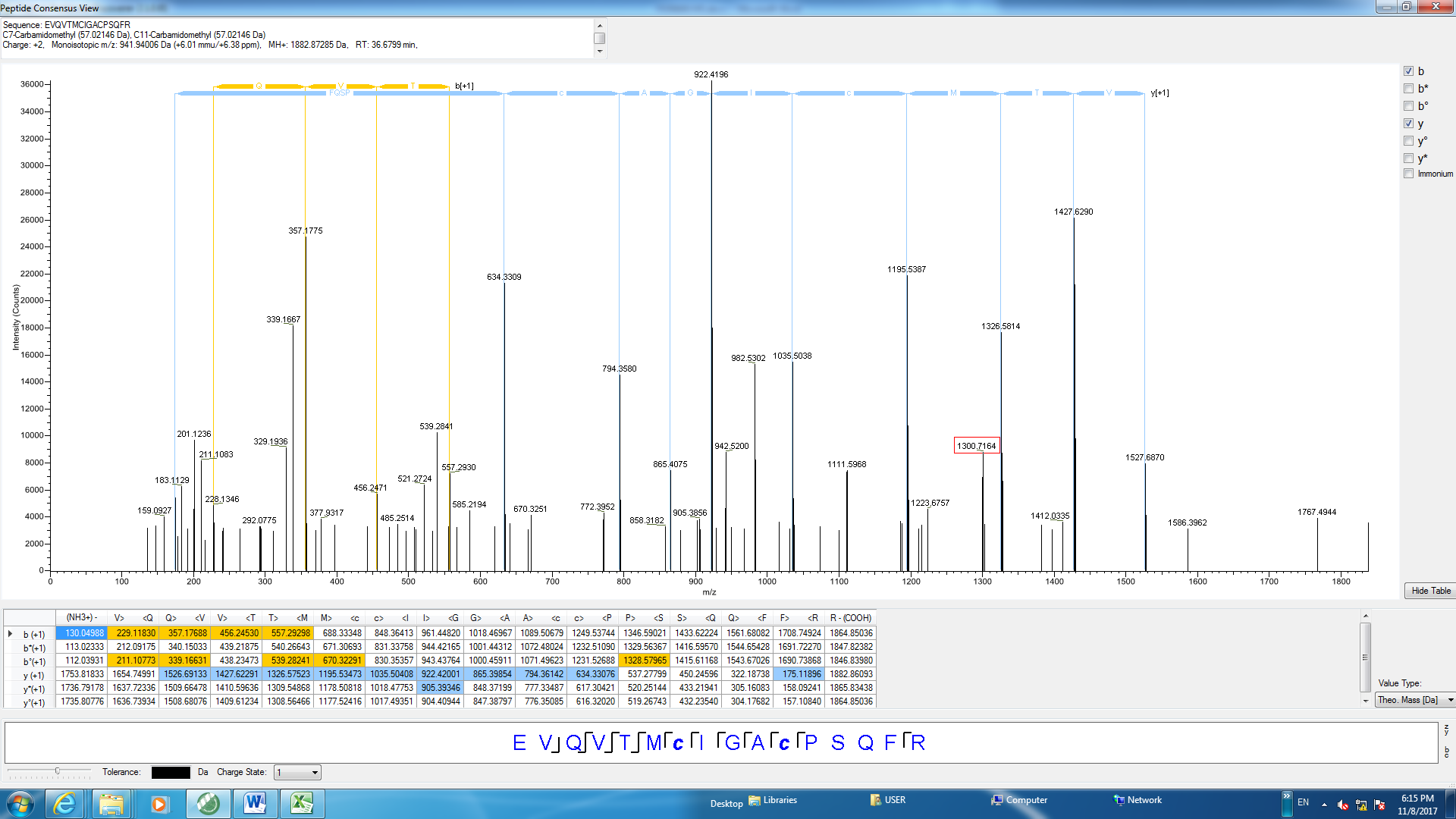


Q05DB4 HEBP2 protein (Fragment) (1/2)


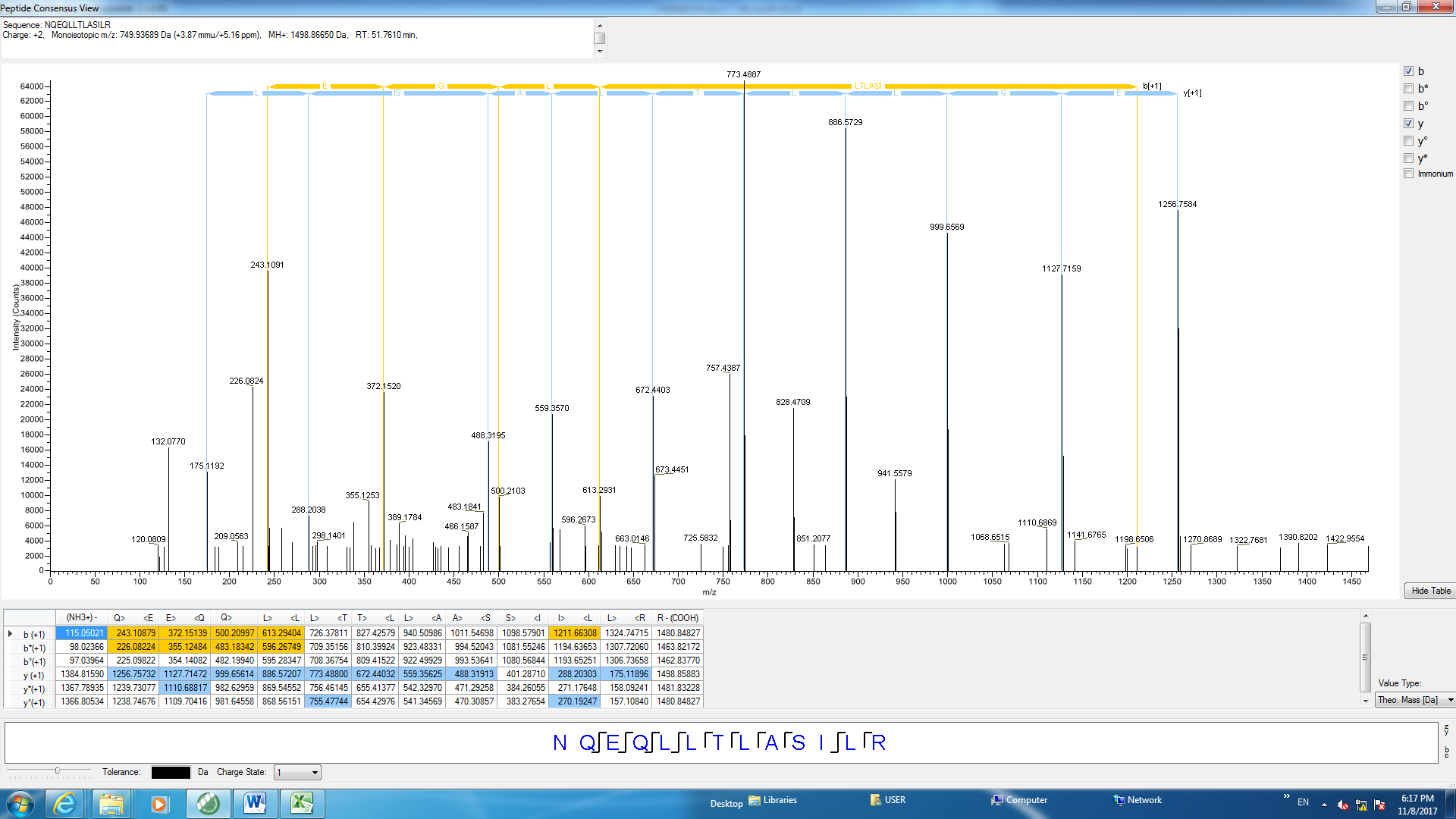


Q05DB4 HEBP2 protein (Fragment) (2/2)


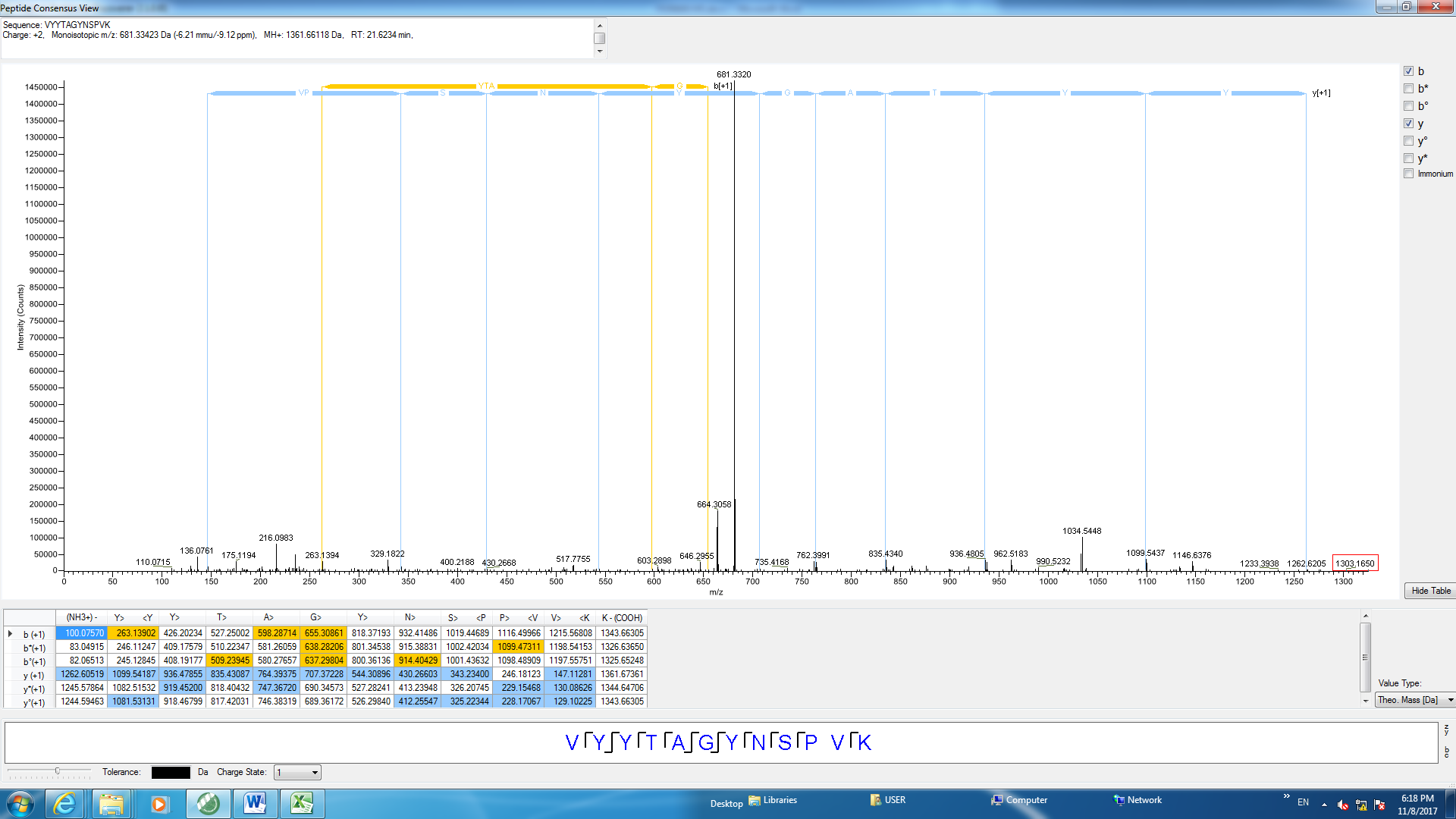


Q6FH62 HSD17B3 protein (1/1)


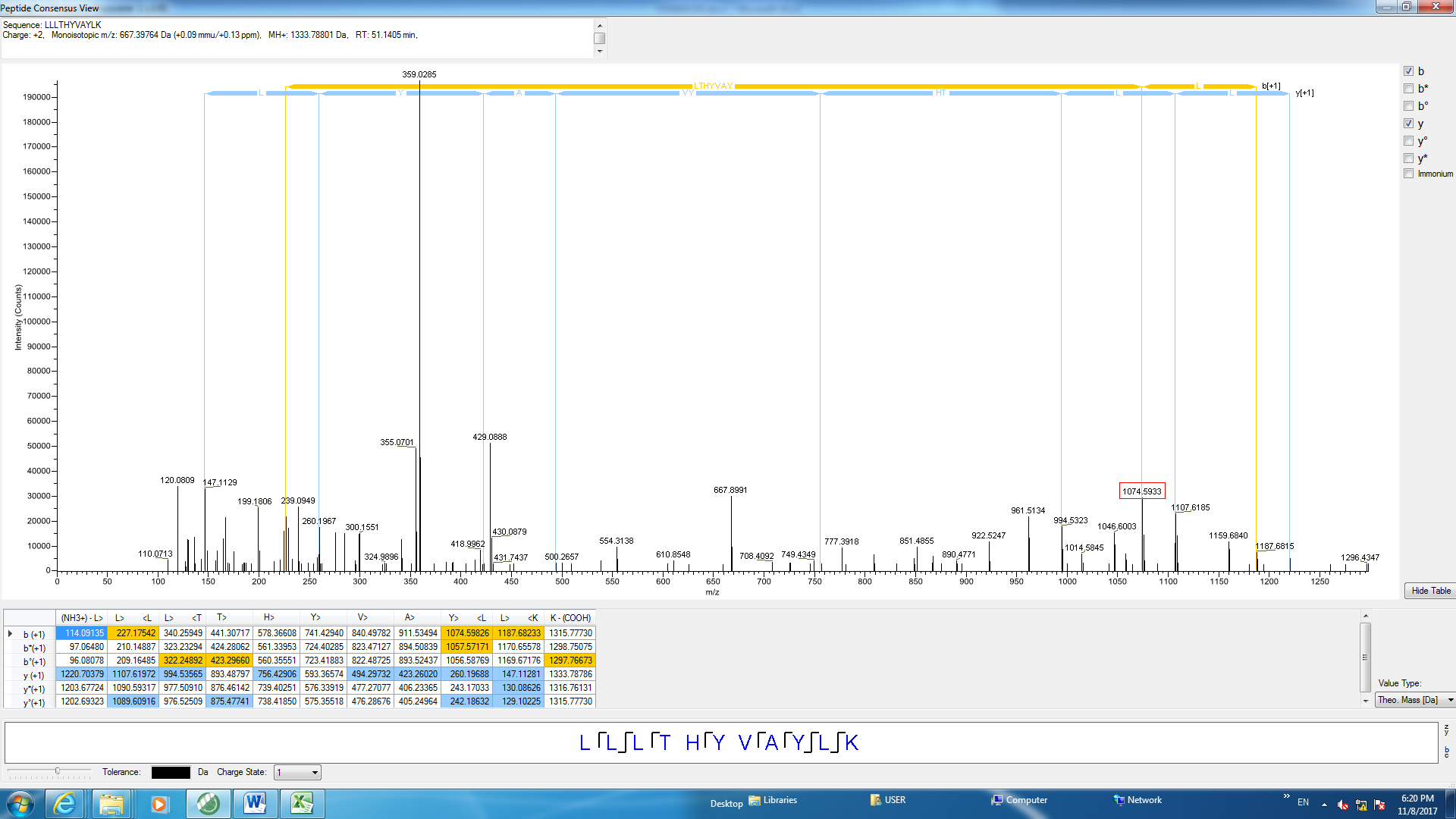


S6B294 IgG L chain (1/1)


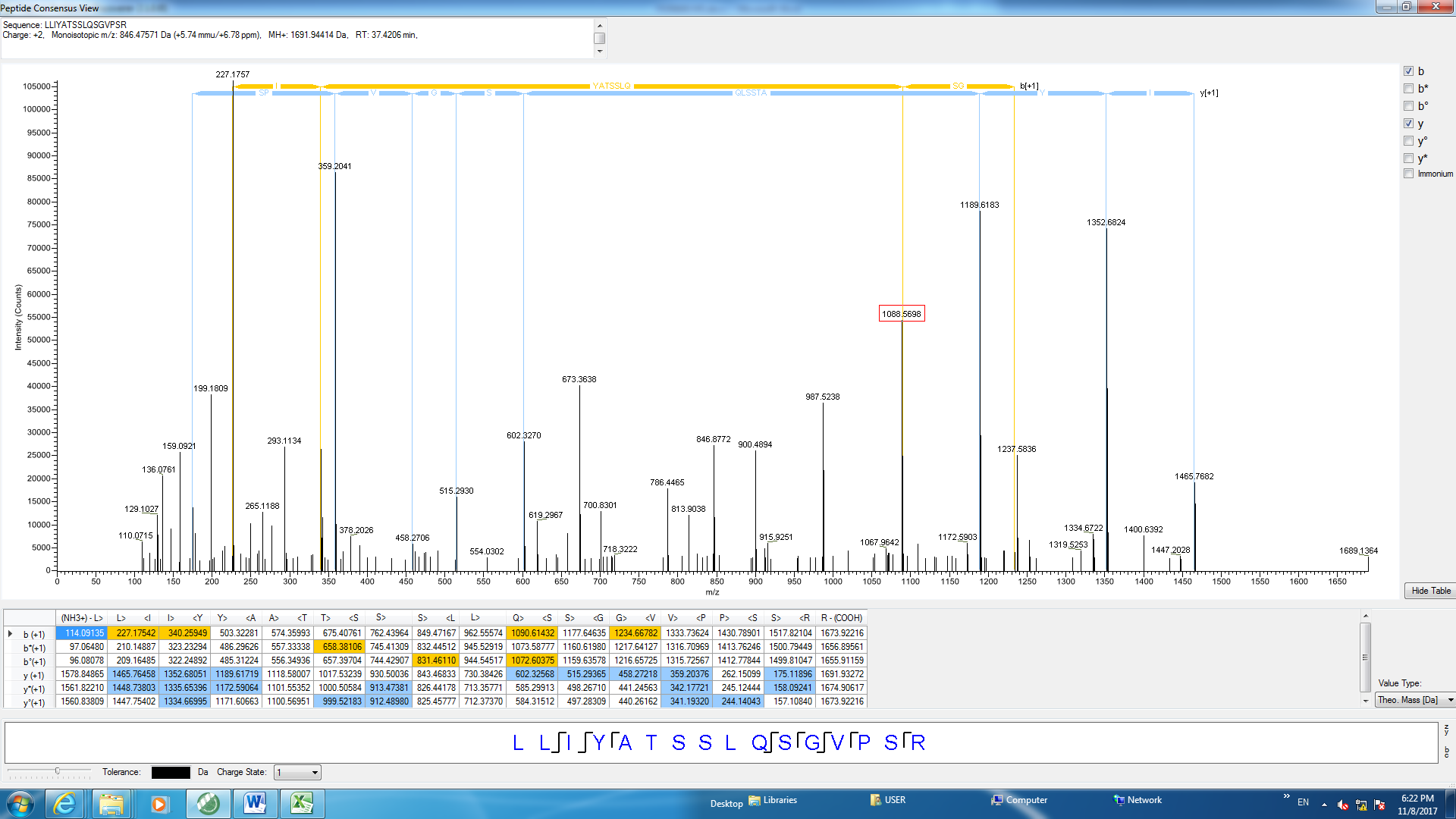


X6R7Y7 Intraflagellar transport protein 25 homolog (1/1)


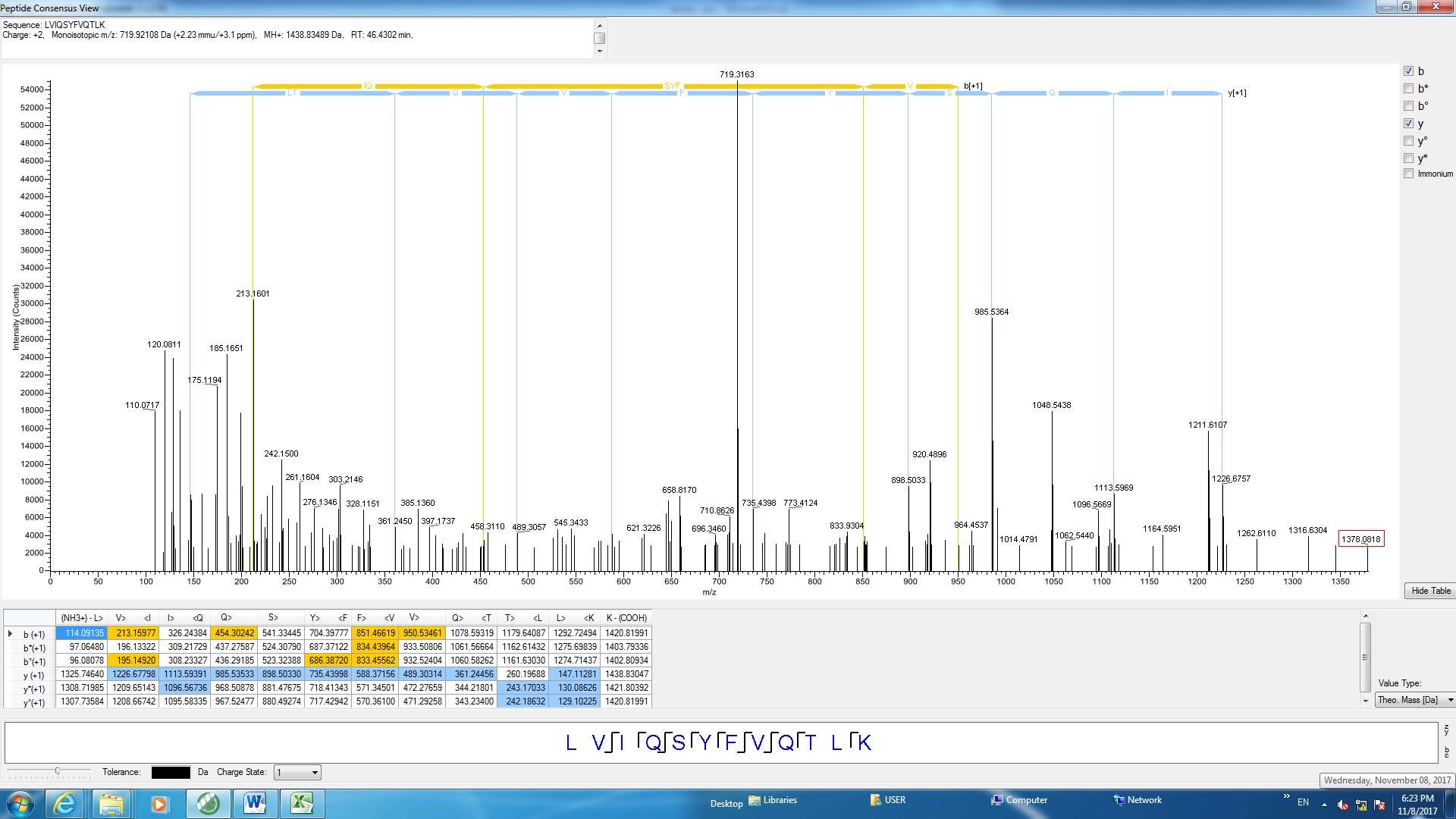


Q96P63-2 Isoform 2 of Serpin B12 (1/1)


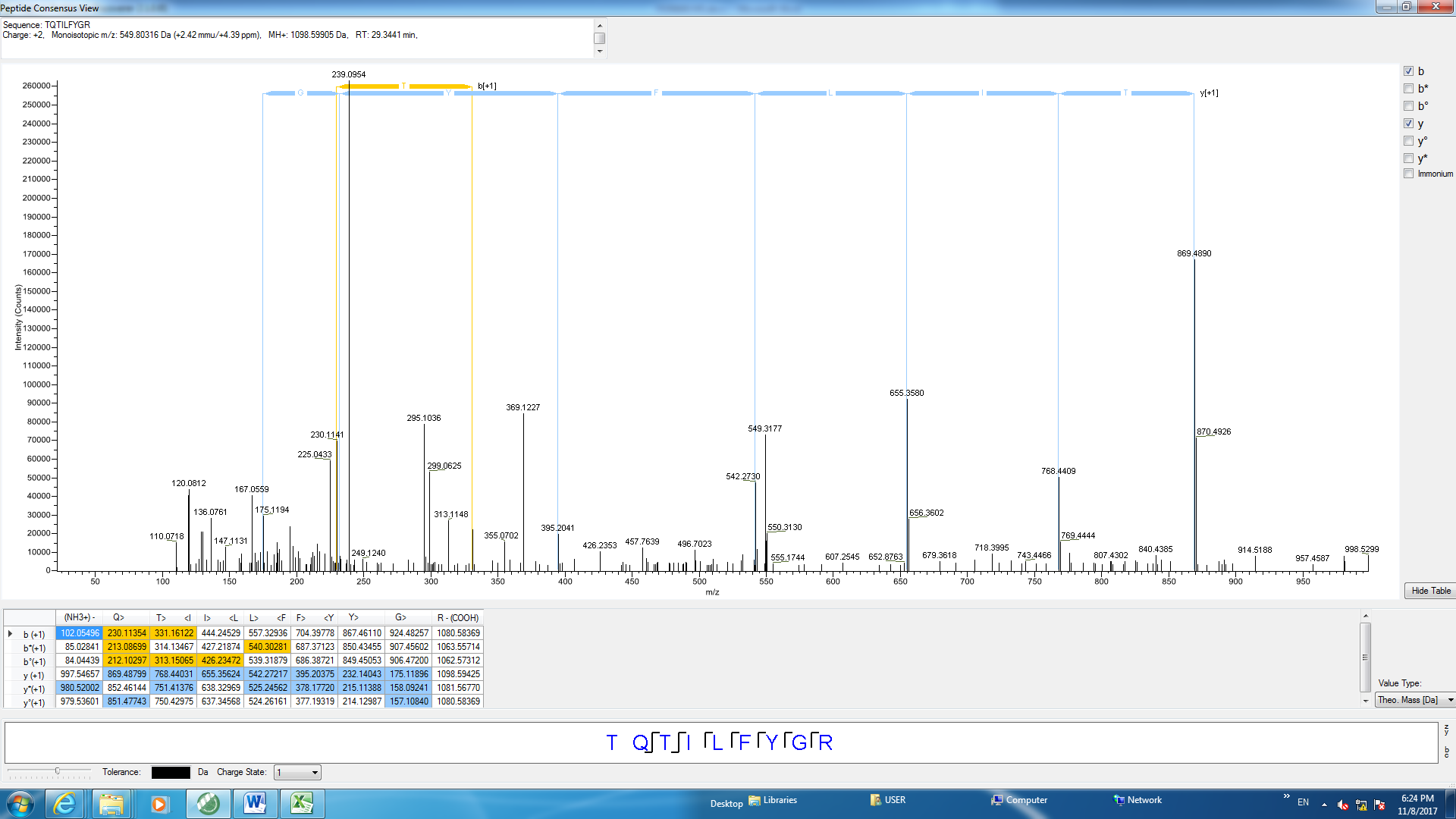


P48304 Lithostathine-1-beta (1/1)


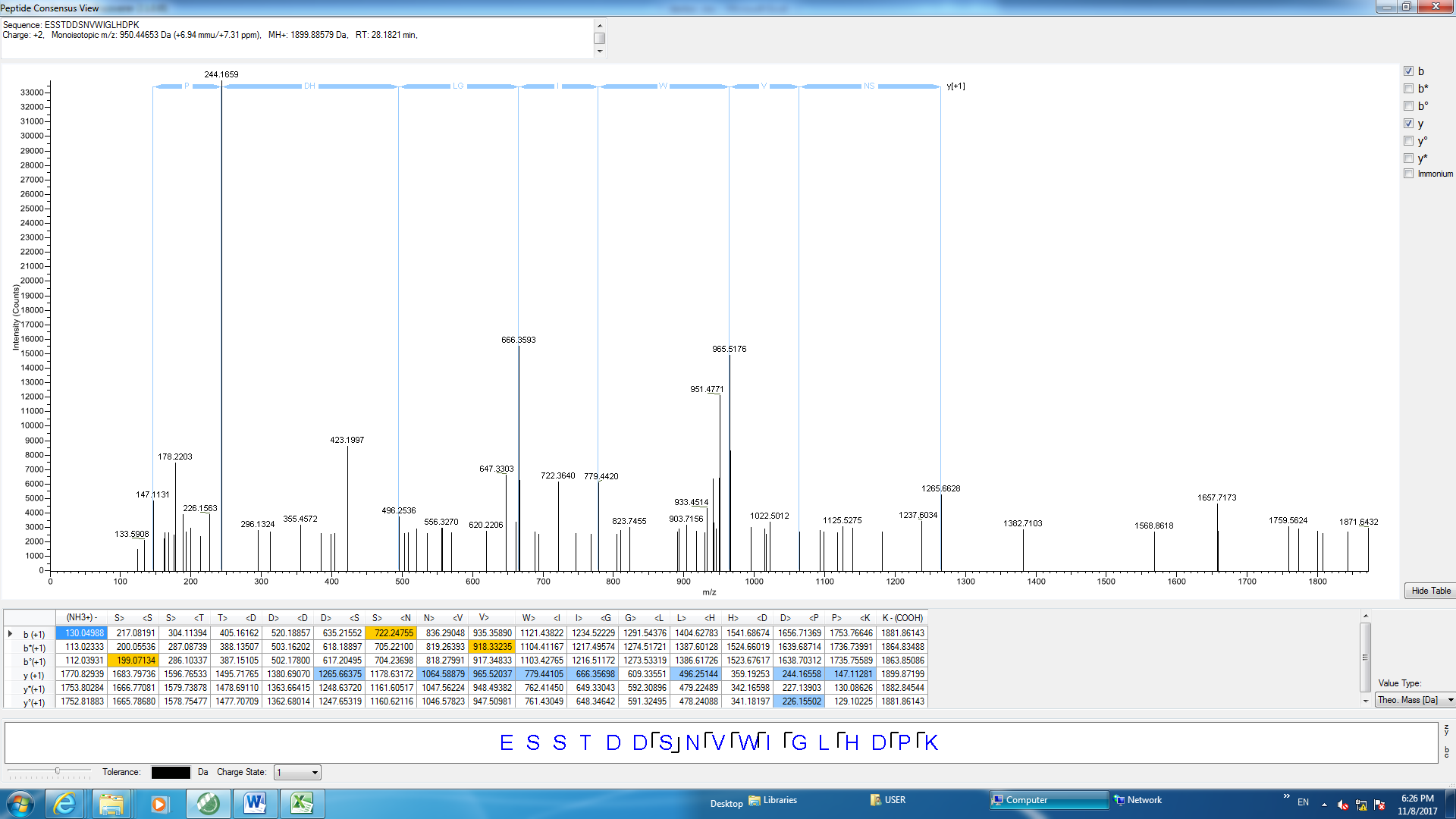


A7Y9J9 Mucin 5AC, oligomeric mucus/gel-forming (1/2)


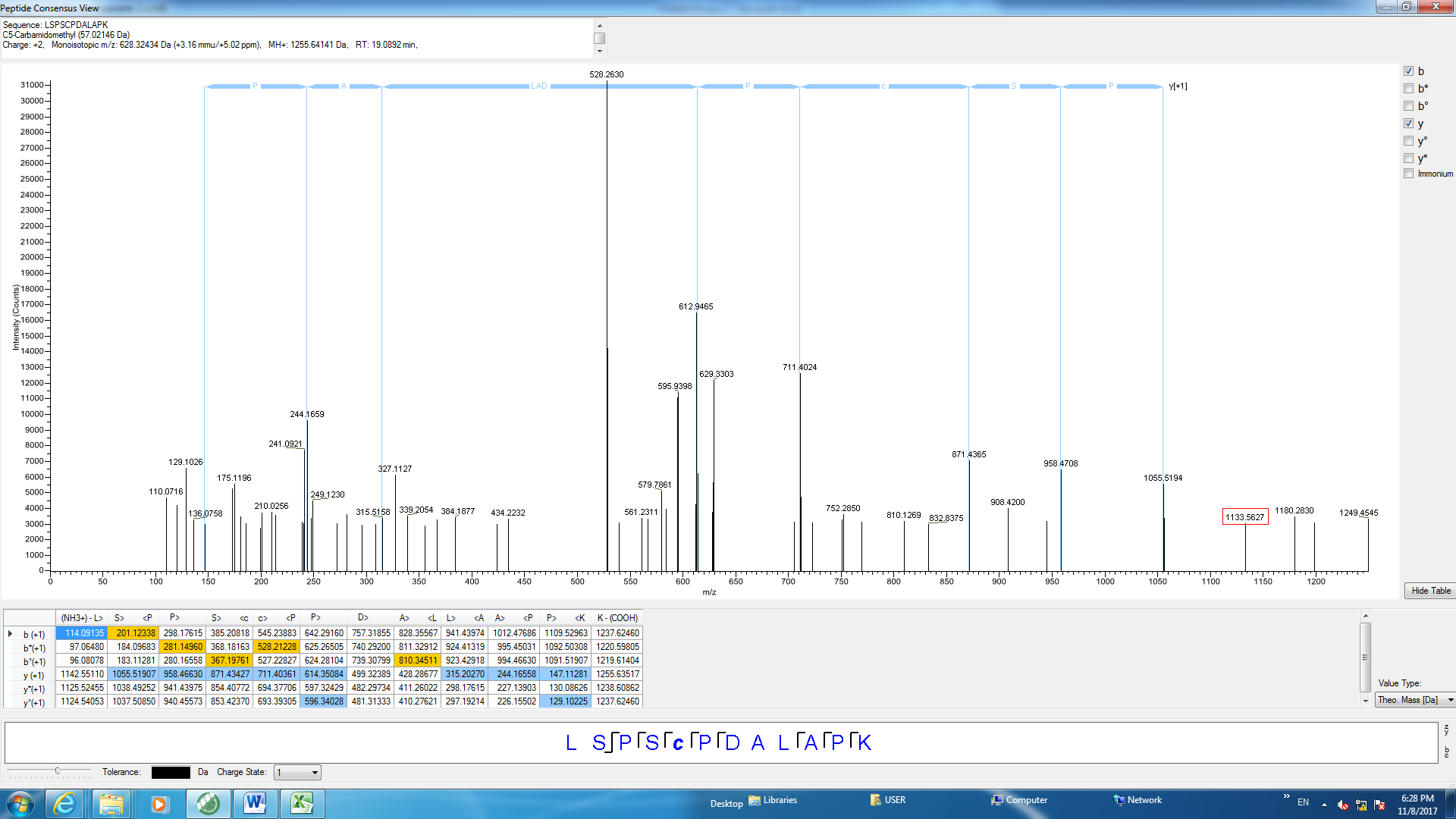


A7Y9J9 Mucin 5AC, oligomeric mucus/gel-forming (2/2)


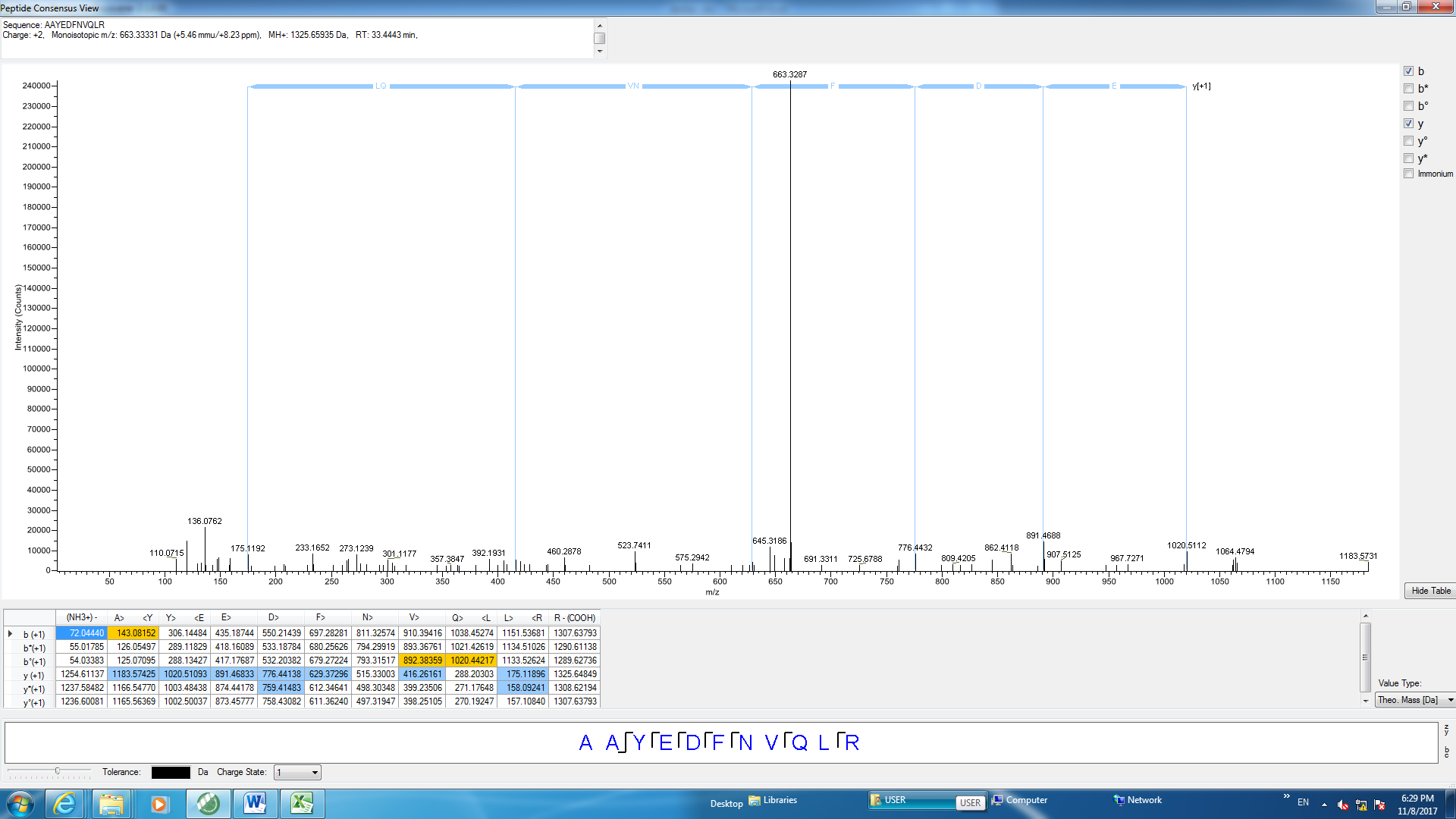


Q8TAX7 Mucin-7 (1/3)


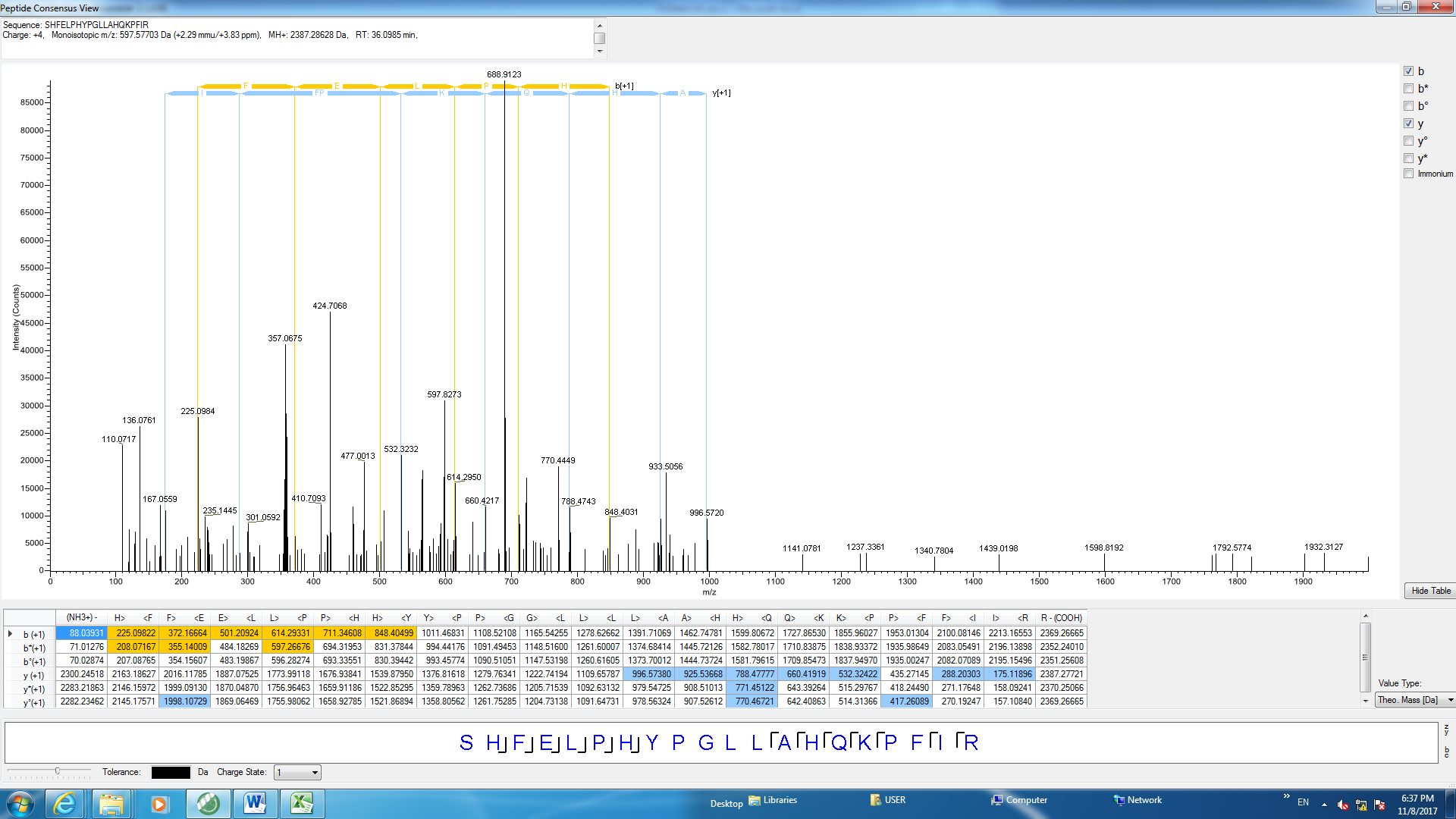


Q8TAX7 Mucin-7 (2/3)


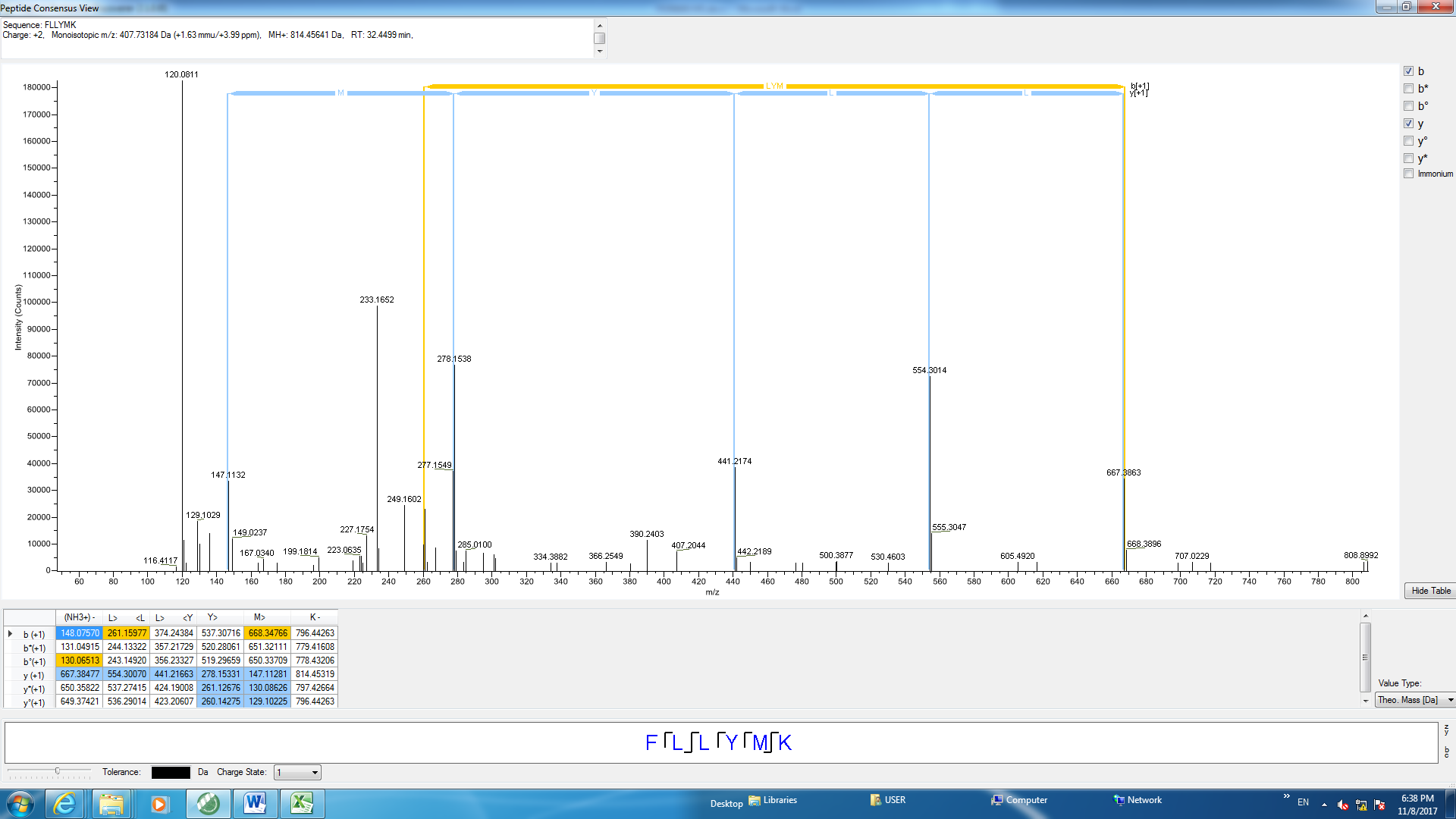


Q8TAX7 Mucin-7 (3/3)


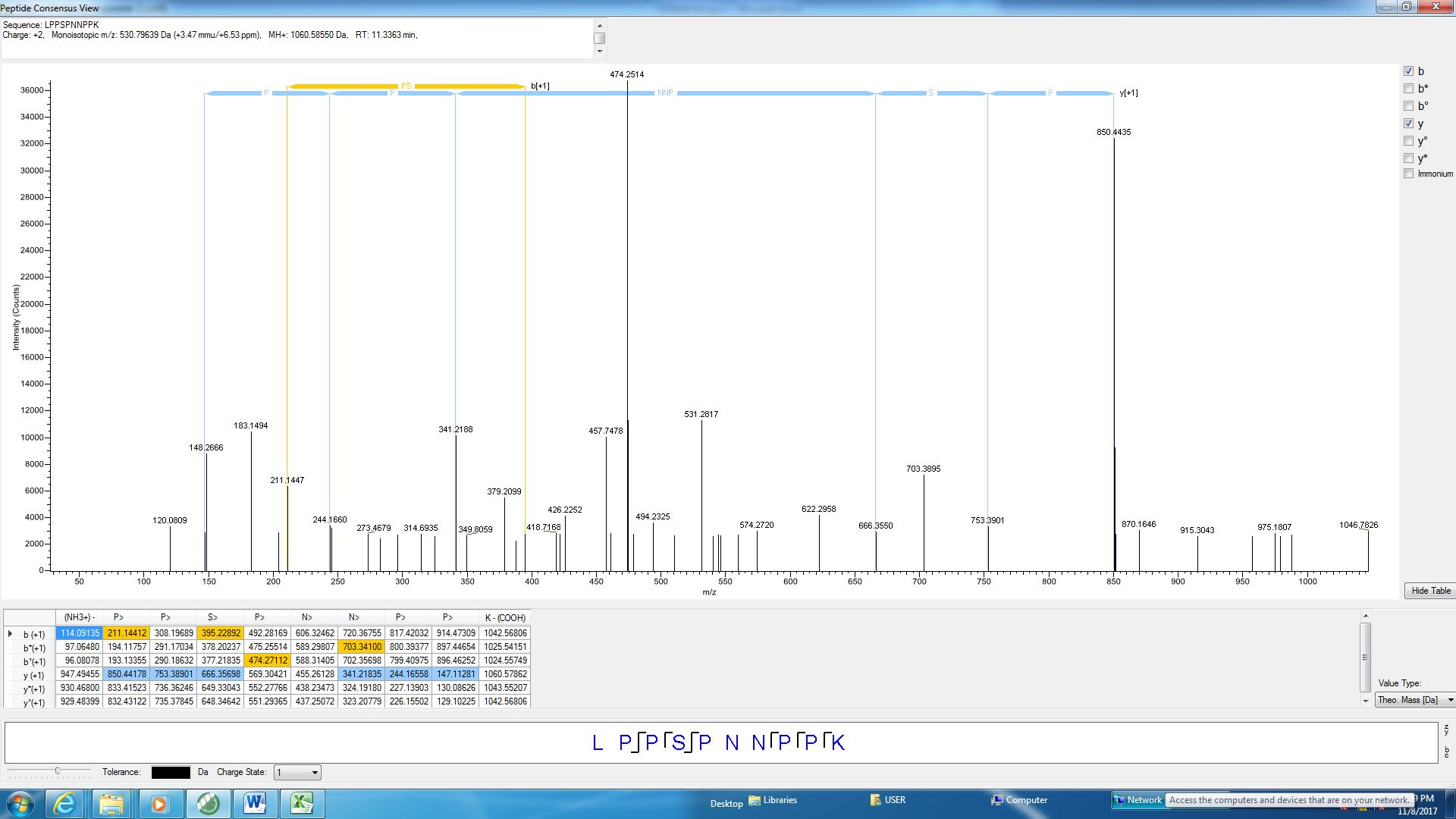


P59665 Neutrophil defensin 1 (1/1)


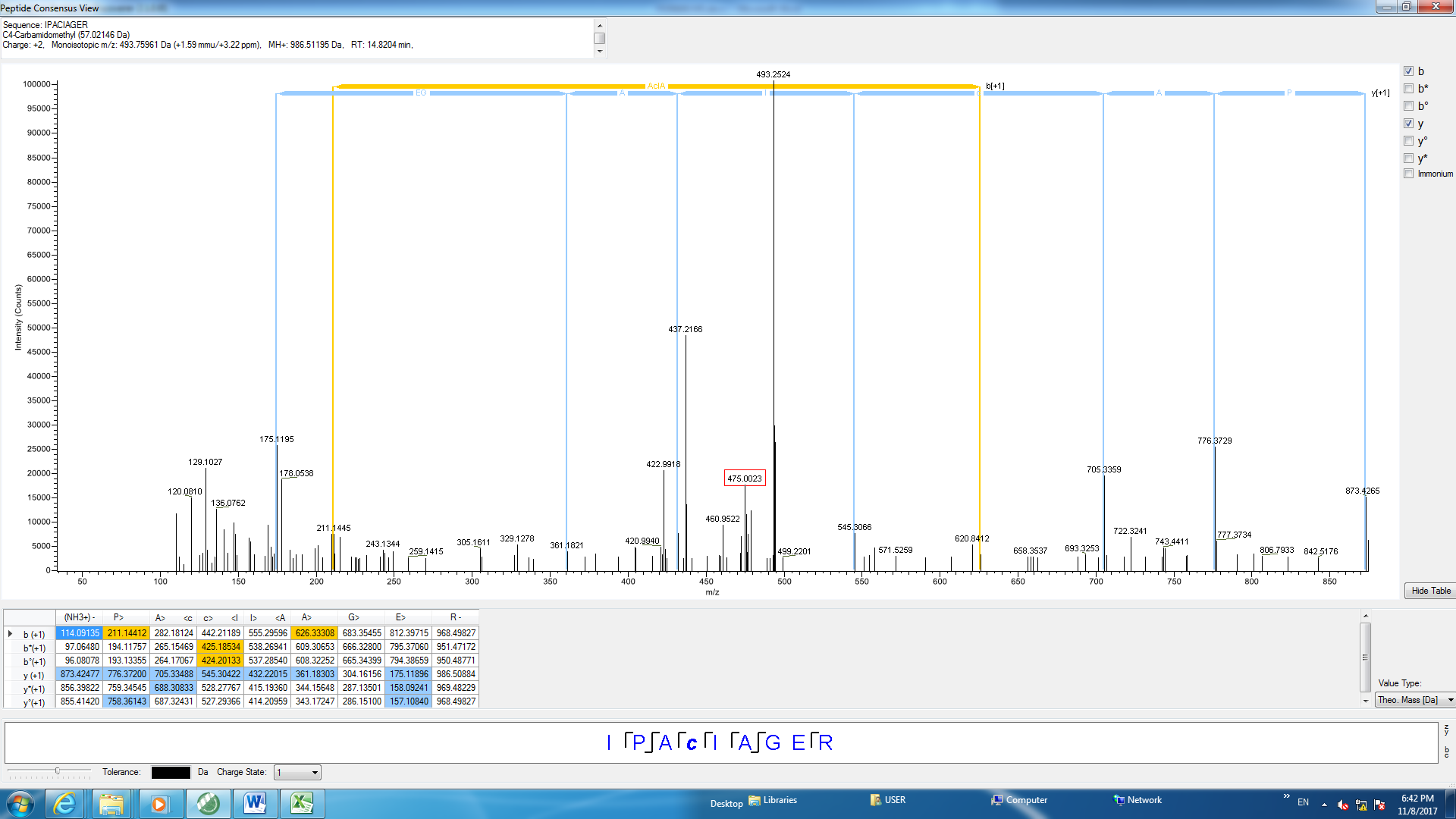


P01833 Polymeric immunoglobulin receptor(1/1)


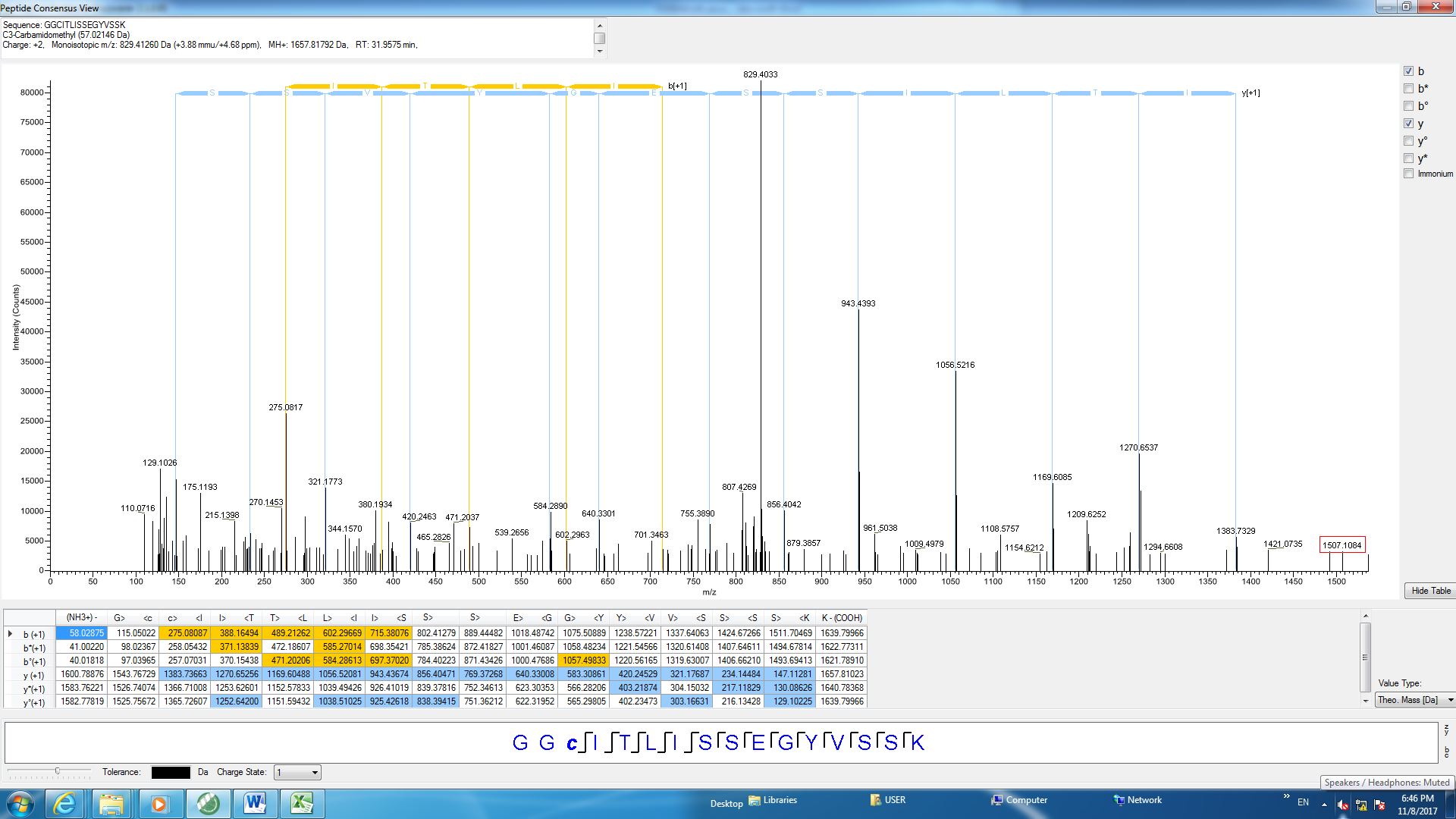


P31151 Protein S100-A7 (1/2)


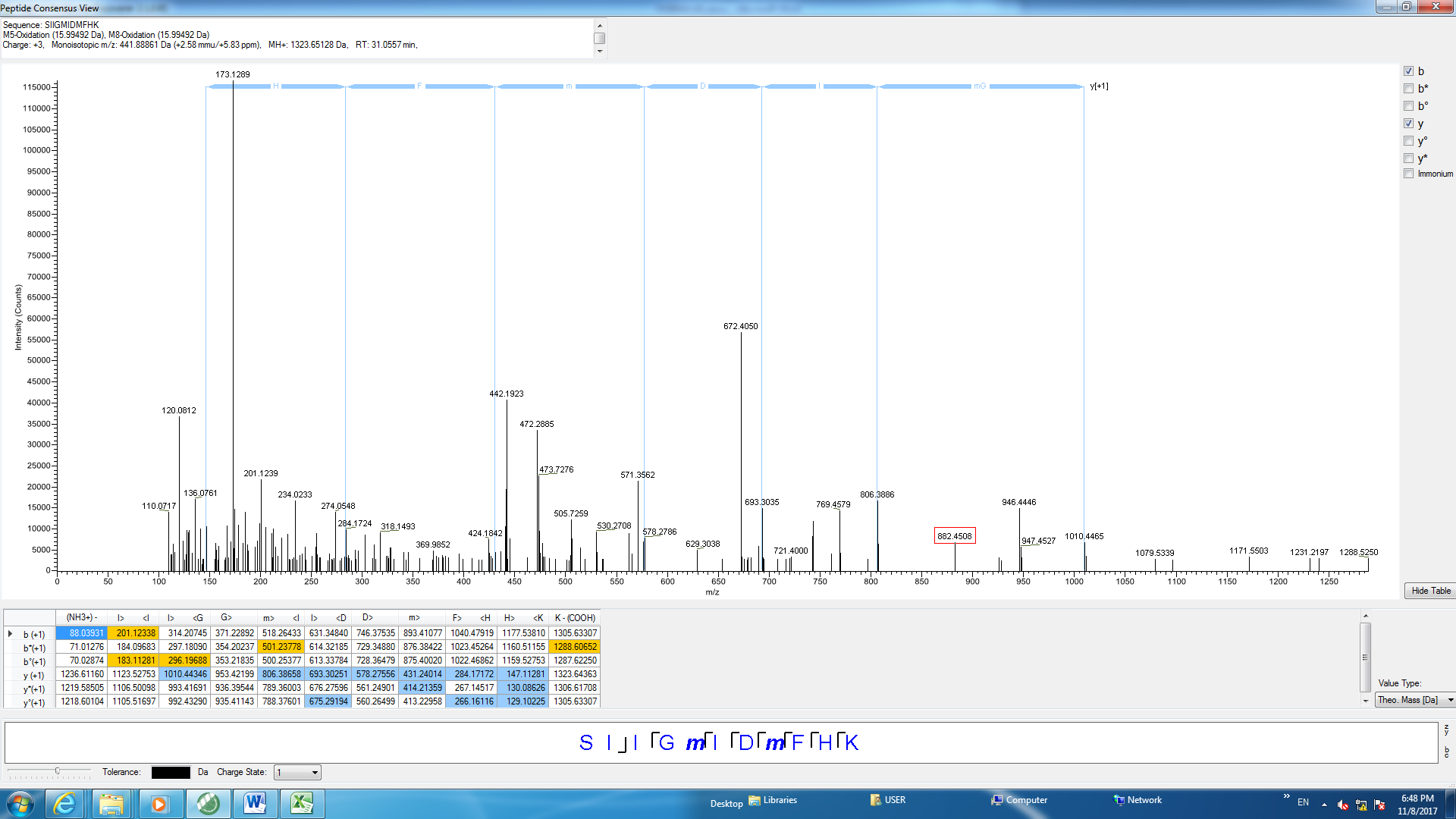


P31151 Protein S100-A7 (2/2)


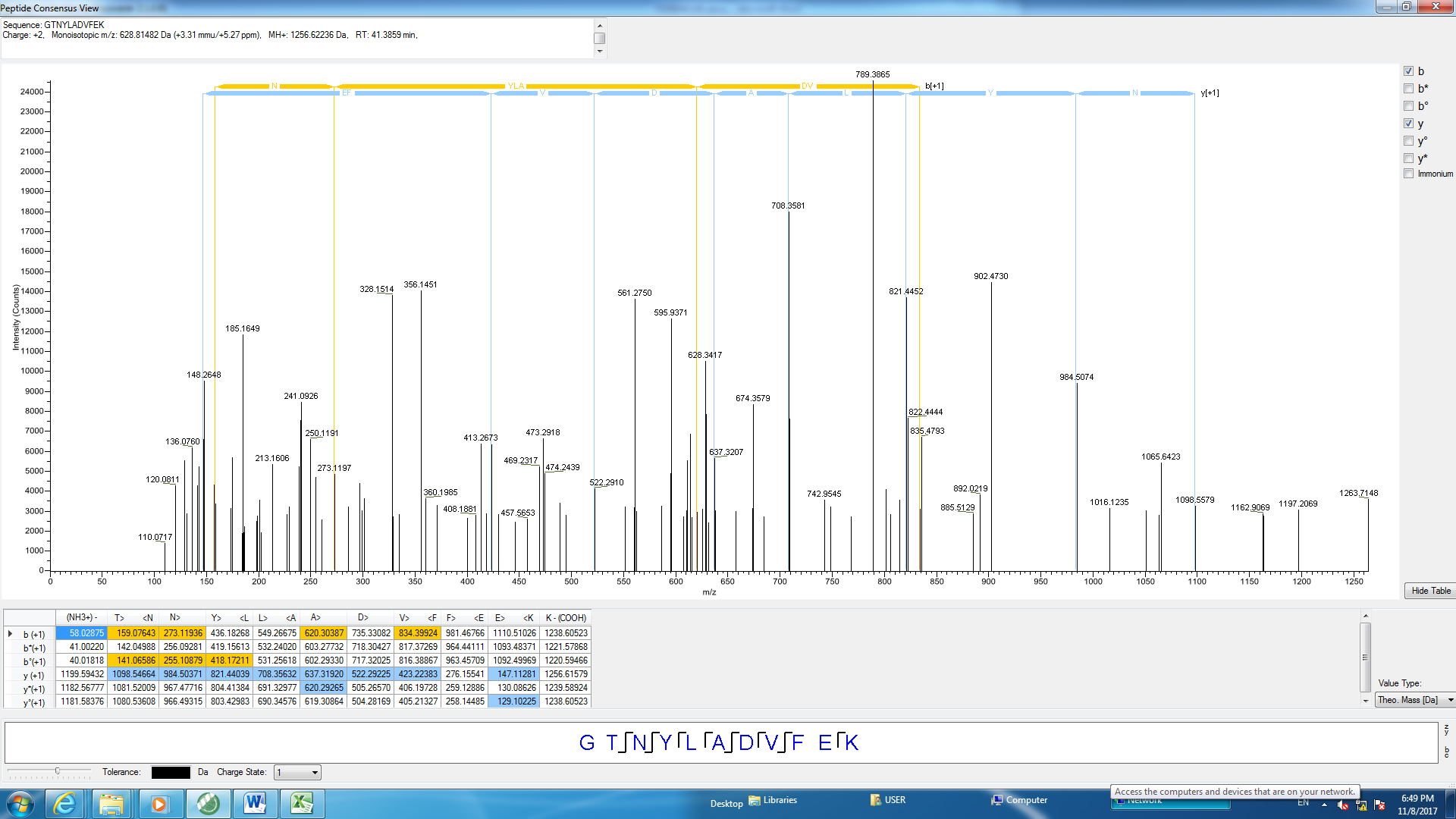


A1A508 PRSS3 protein (1/1)


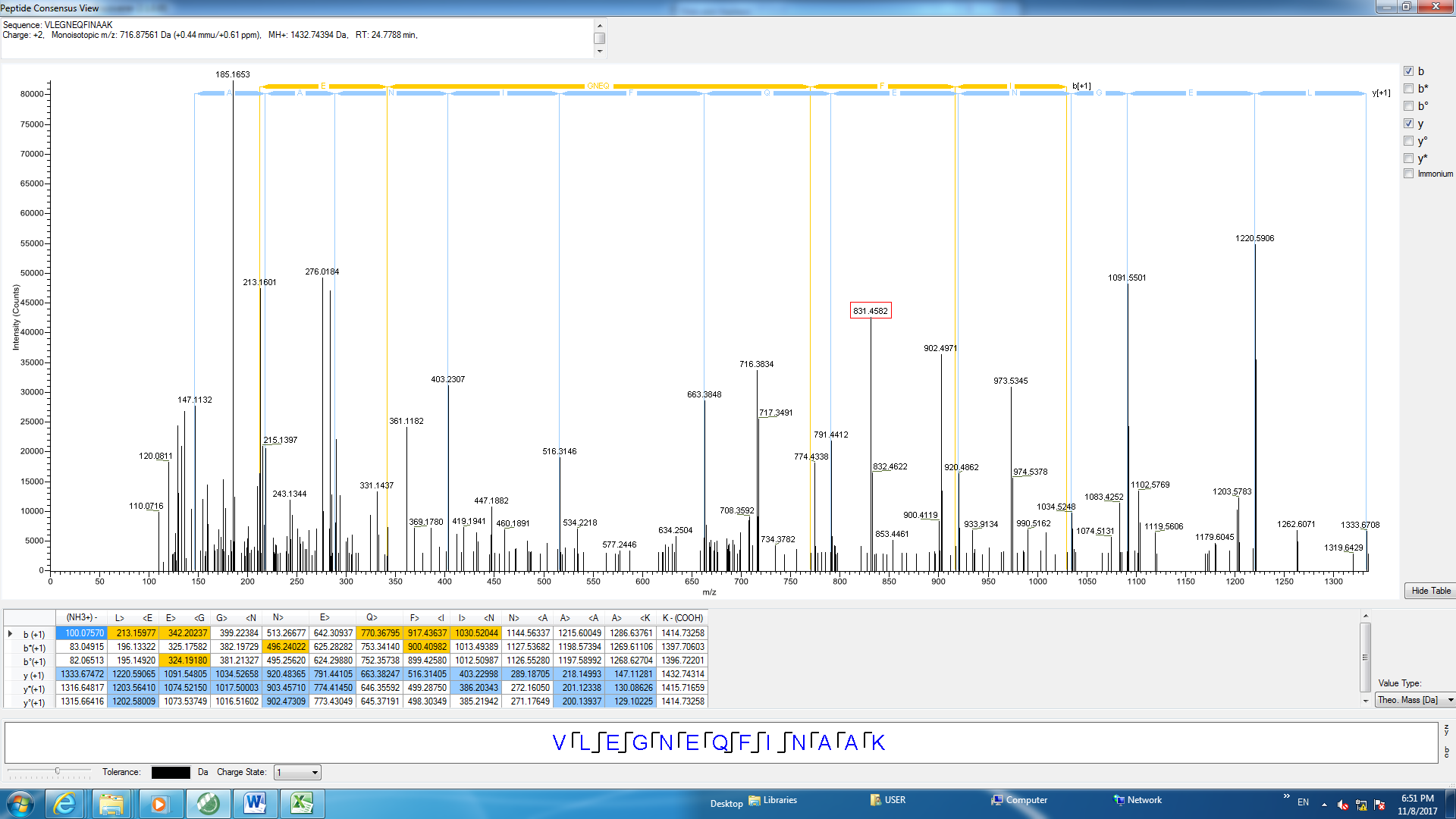


A0A158RFU6 RAB7, member RAS oncogene family, isoform CRA_a (1/1)


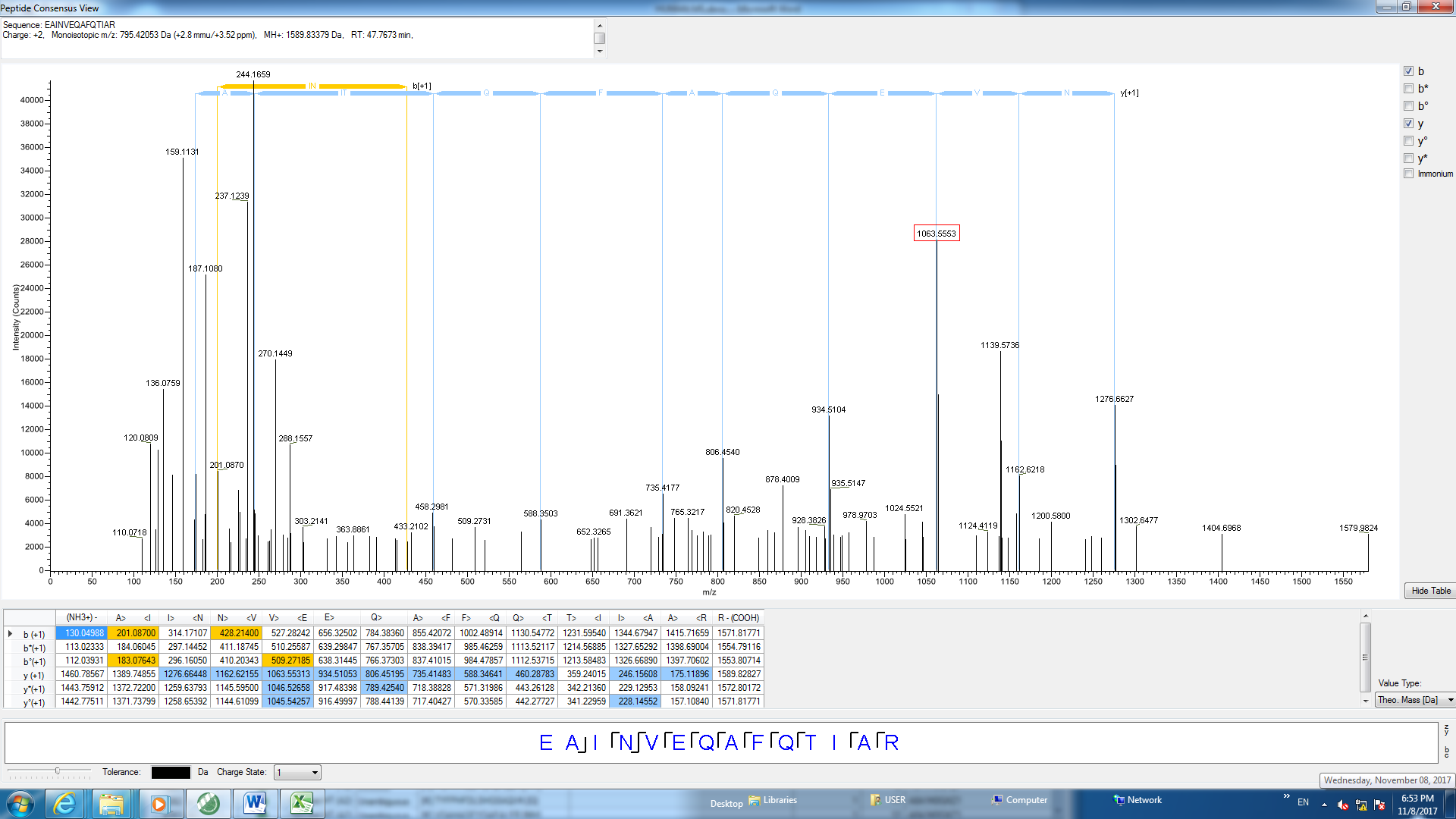


P29508 Serpin B3 (1/1)


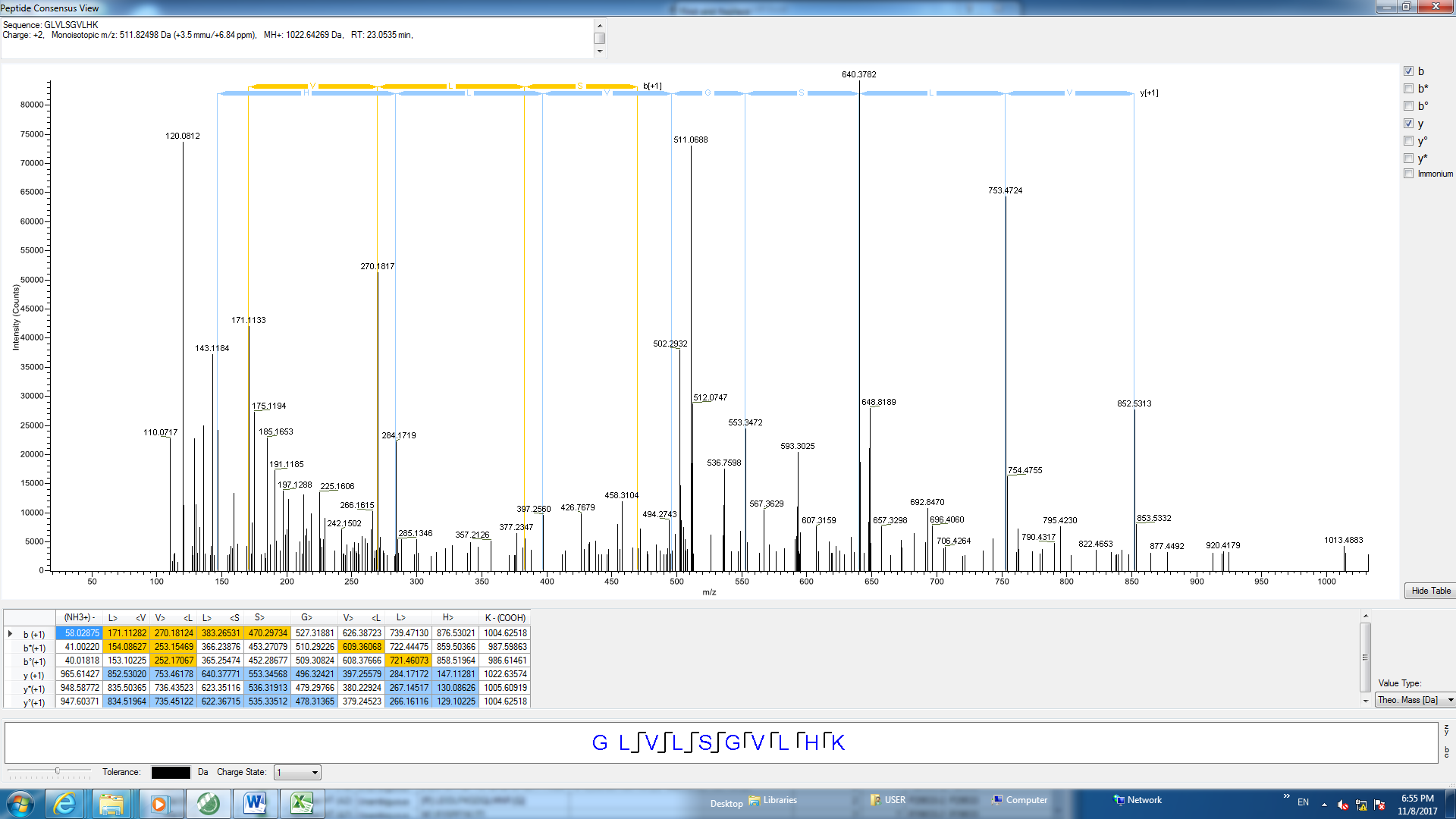


H9ZYJ2 Thioredoxin (1/2)


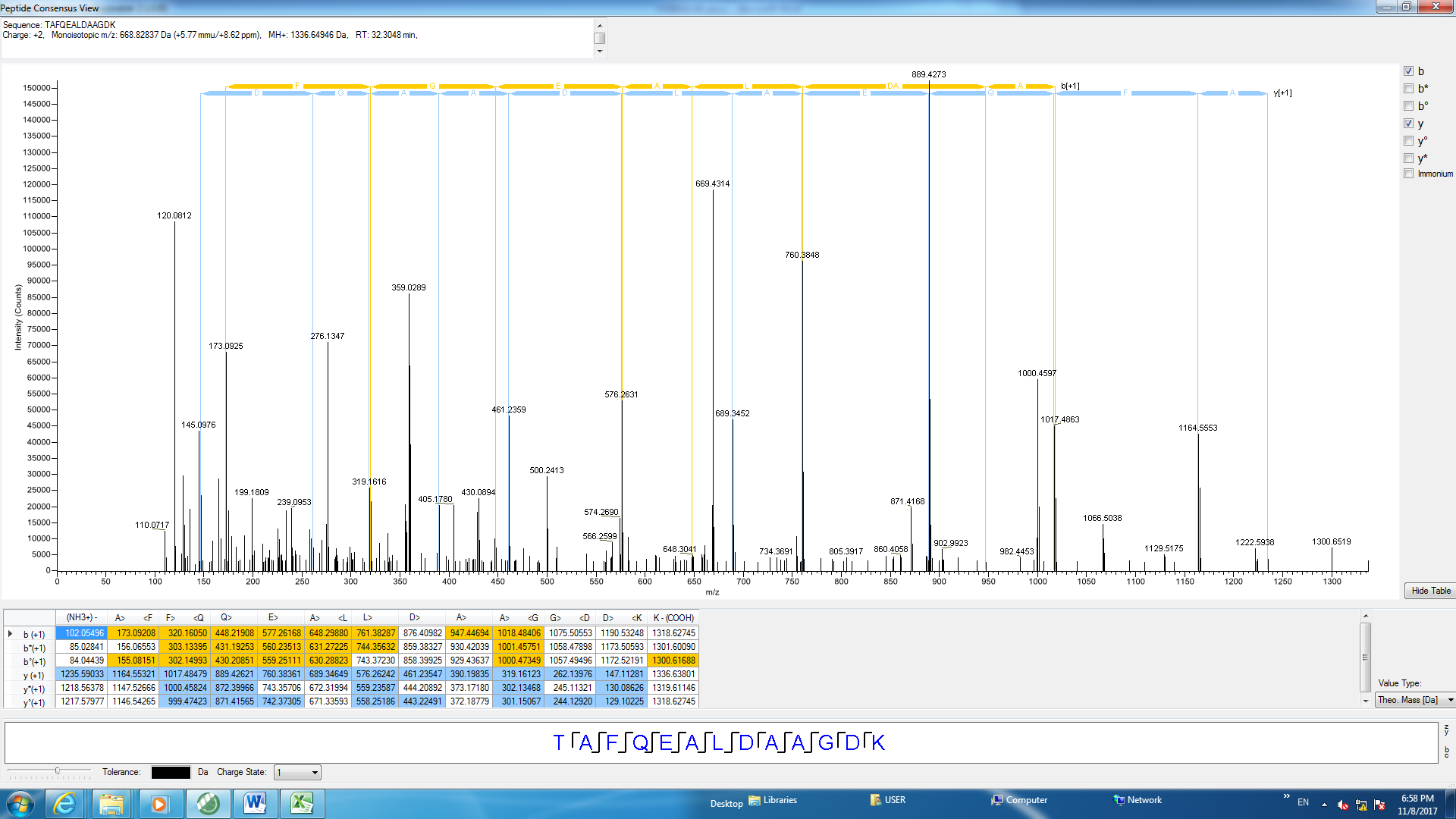


H9ZYJ2 Thioredoxin (2/2)


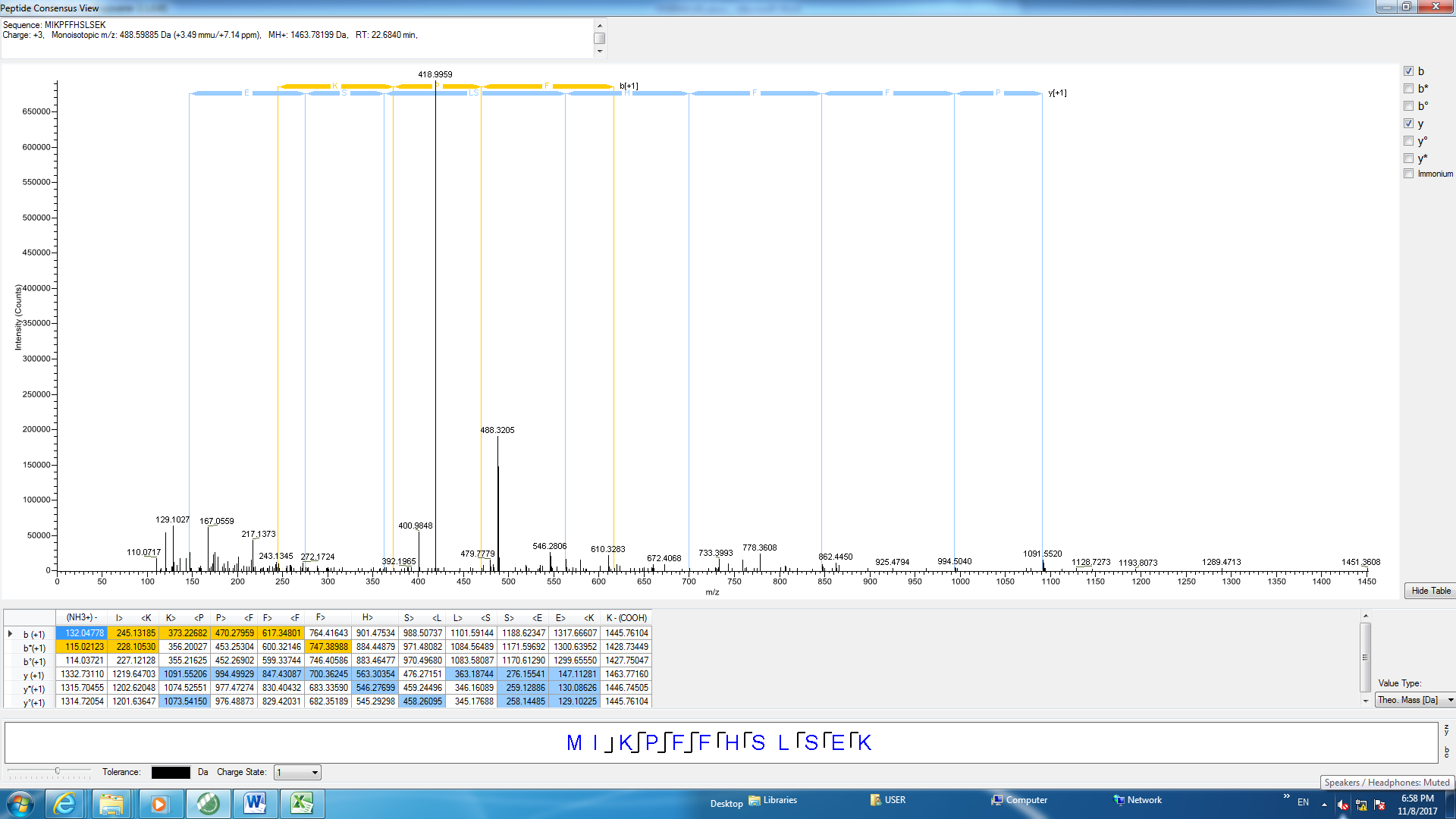


Q03403 Trefoil factor 2 (1/1)


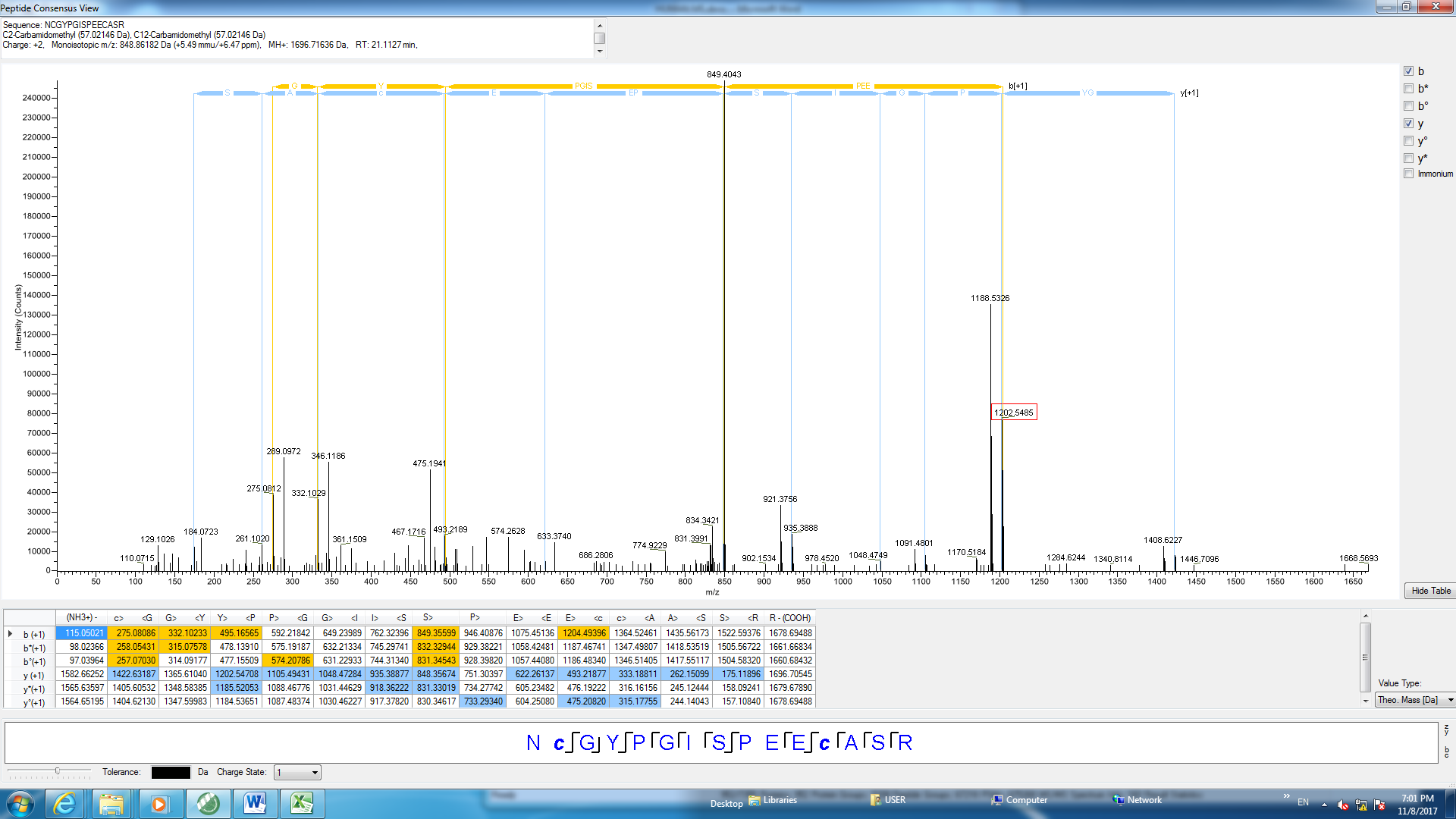


A0A0A6YYJ4 Trefoil factor 3 (1/4)


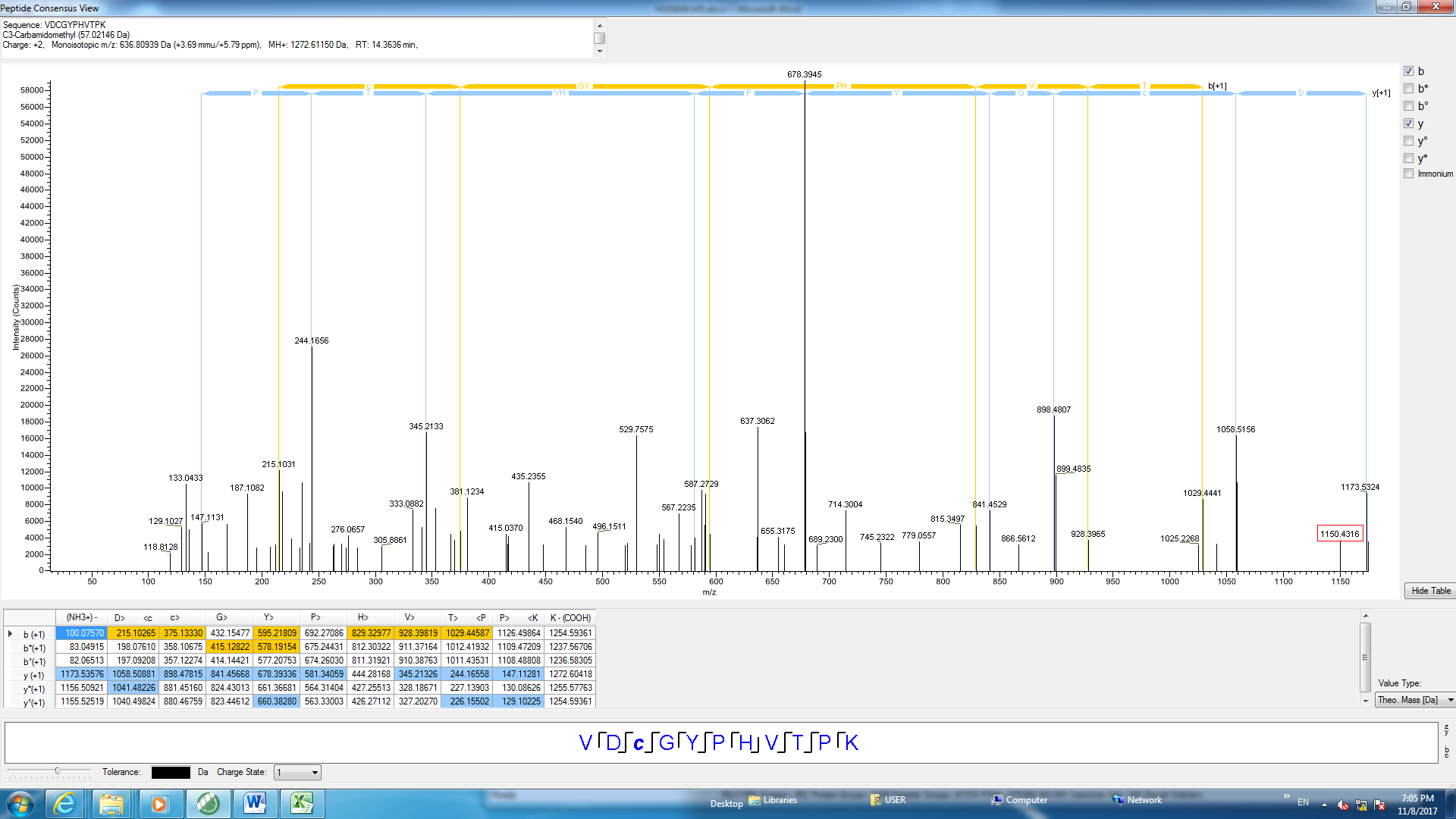


A0A0A6YYJ4 Trefoil factor 3 (2/4)


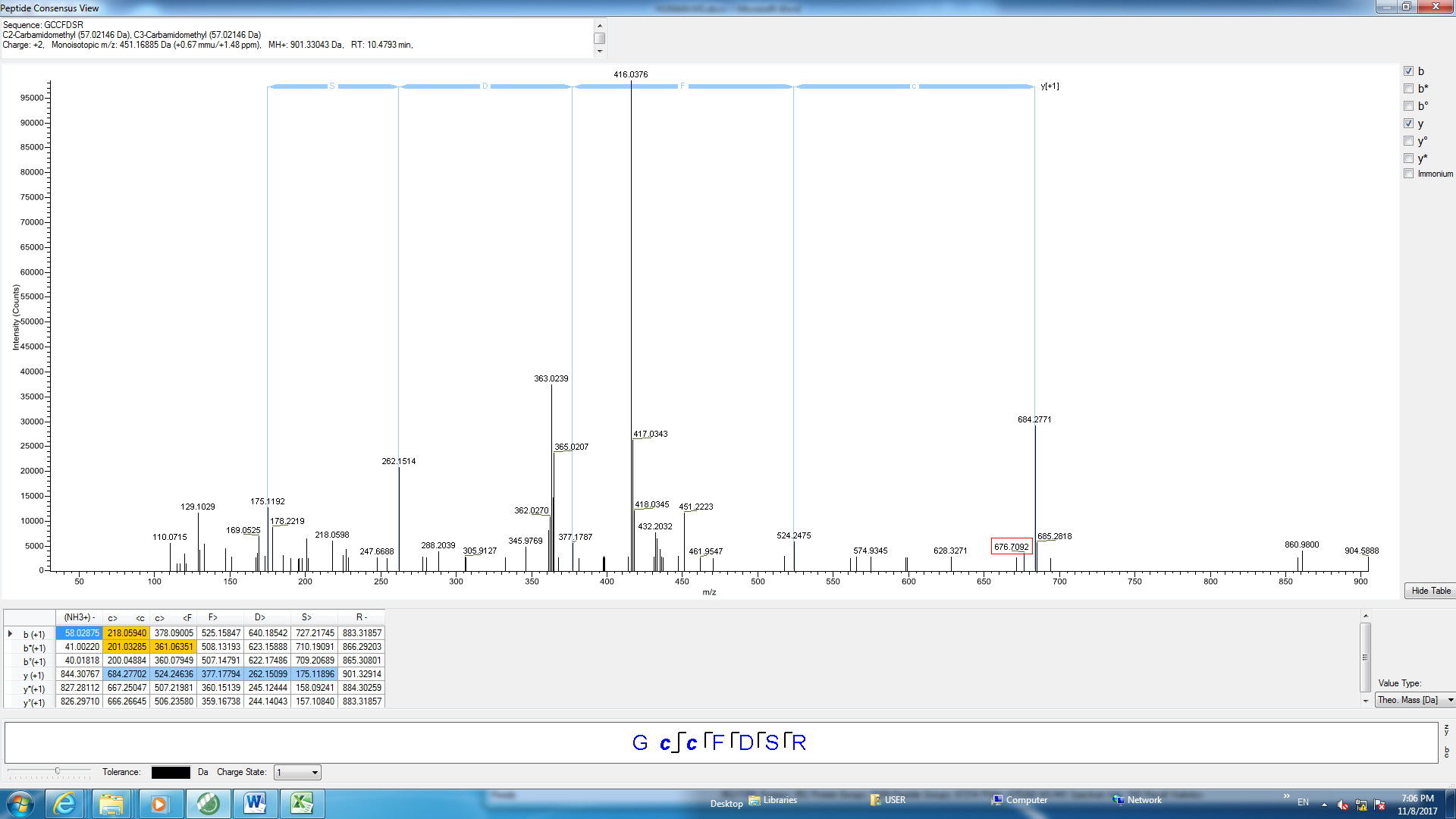


A0A0A6YYJ4 Trefoil factor 3 (3/4)


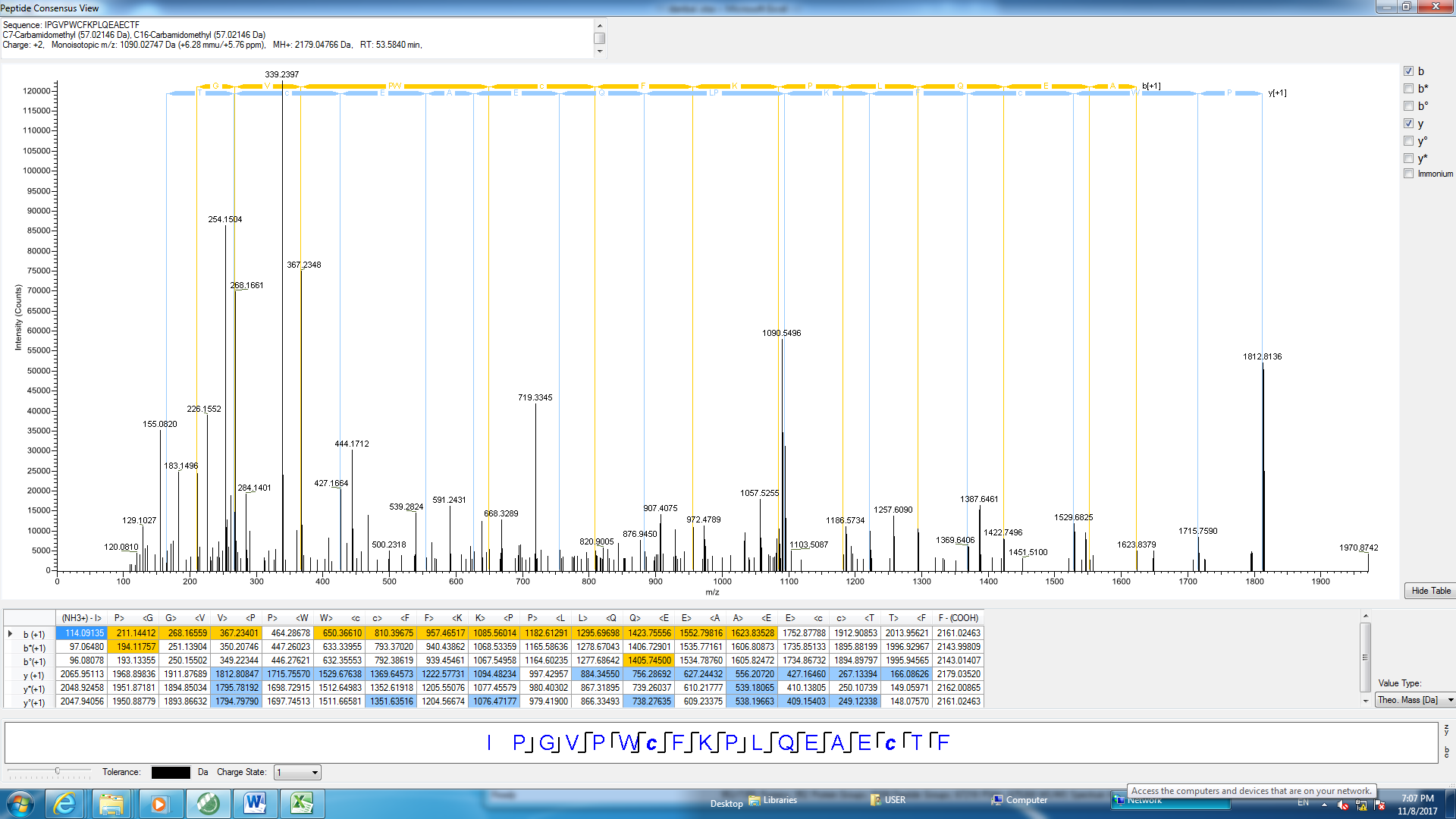


A0A0A6YYJ4 Trefoil factor 3 (4/4)


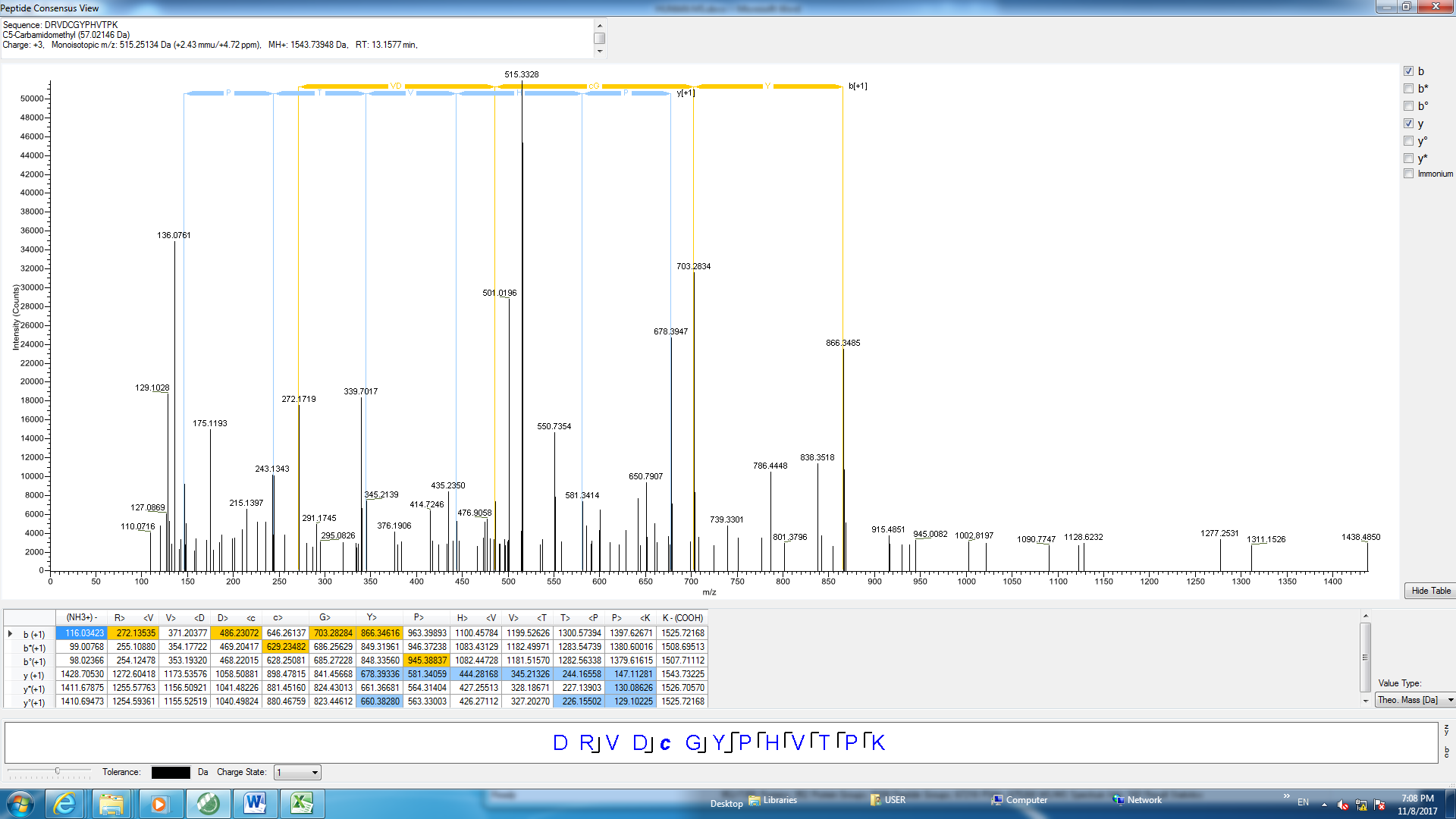


Q6N092 Uncharacterized protein DKFZp686K18196 (Fragment) (1/3)


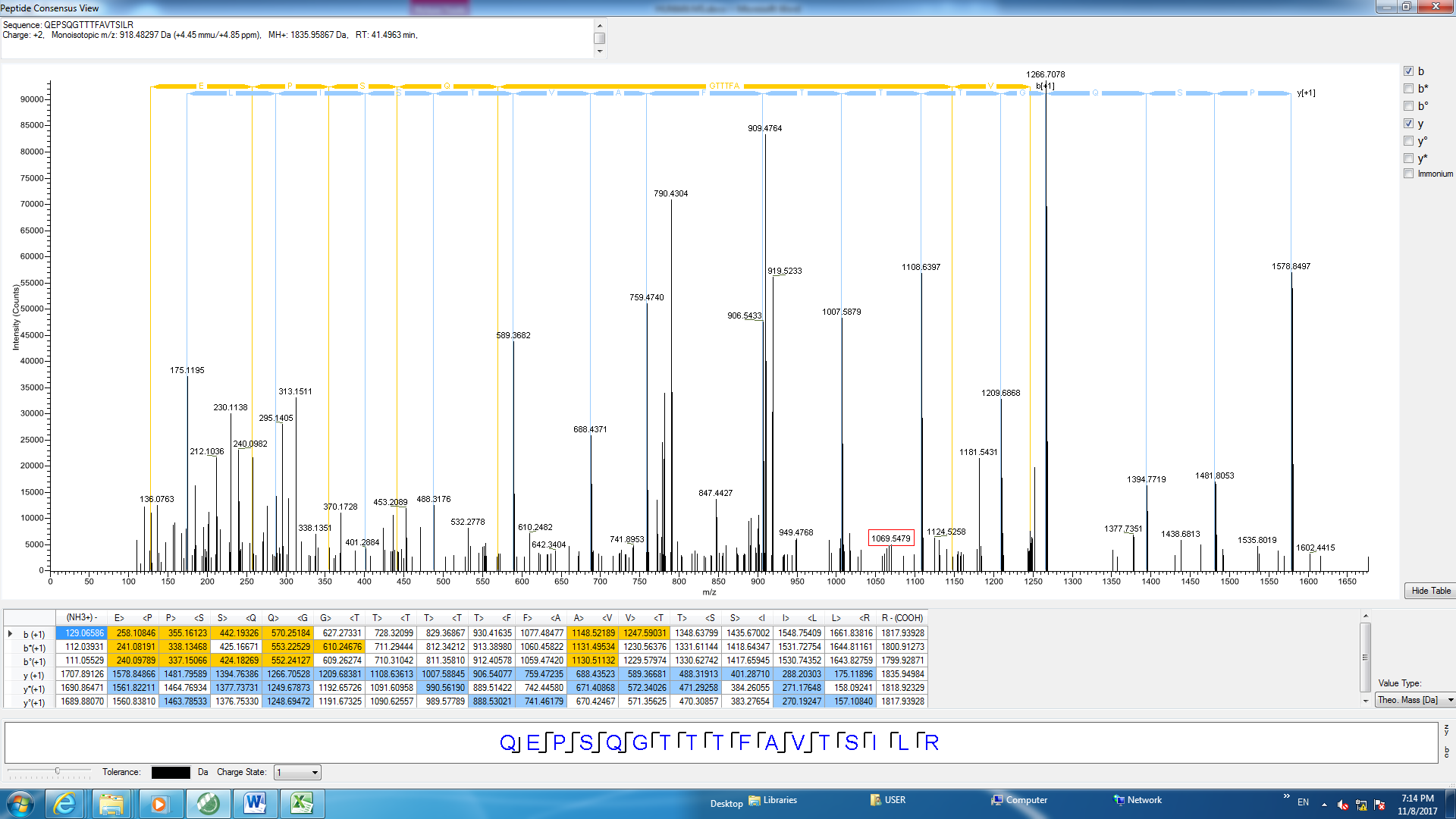


Q6N092 Uncharacterized protein DKFZp686K18196 (Fragment) (2/3)


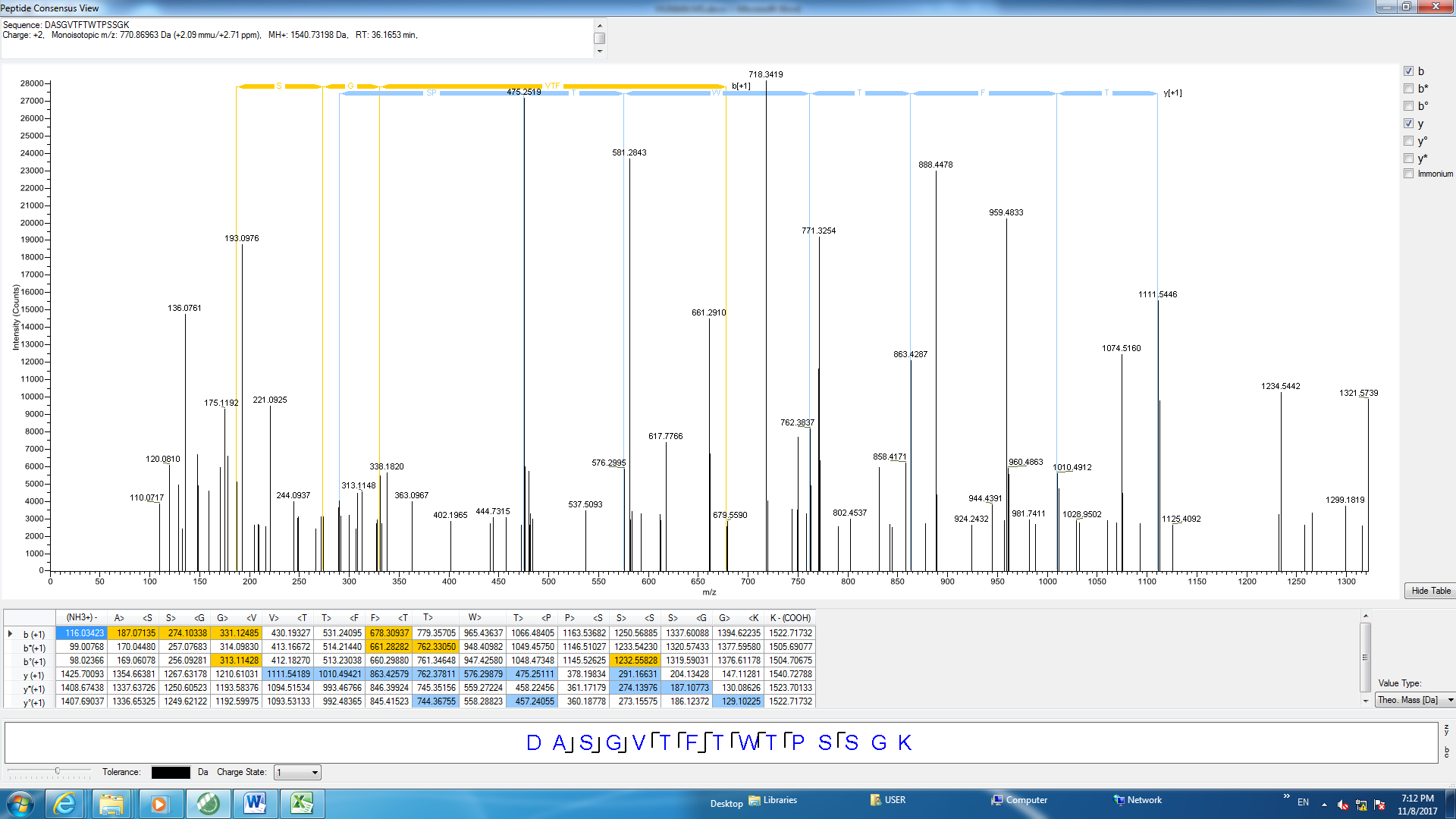


Q6N092 Uncharacterized protein DKFZp686K18196 (Fragment) (3/3)


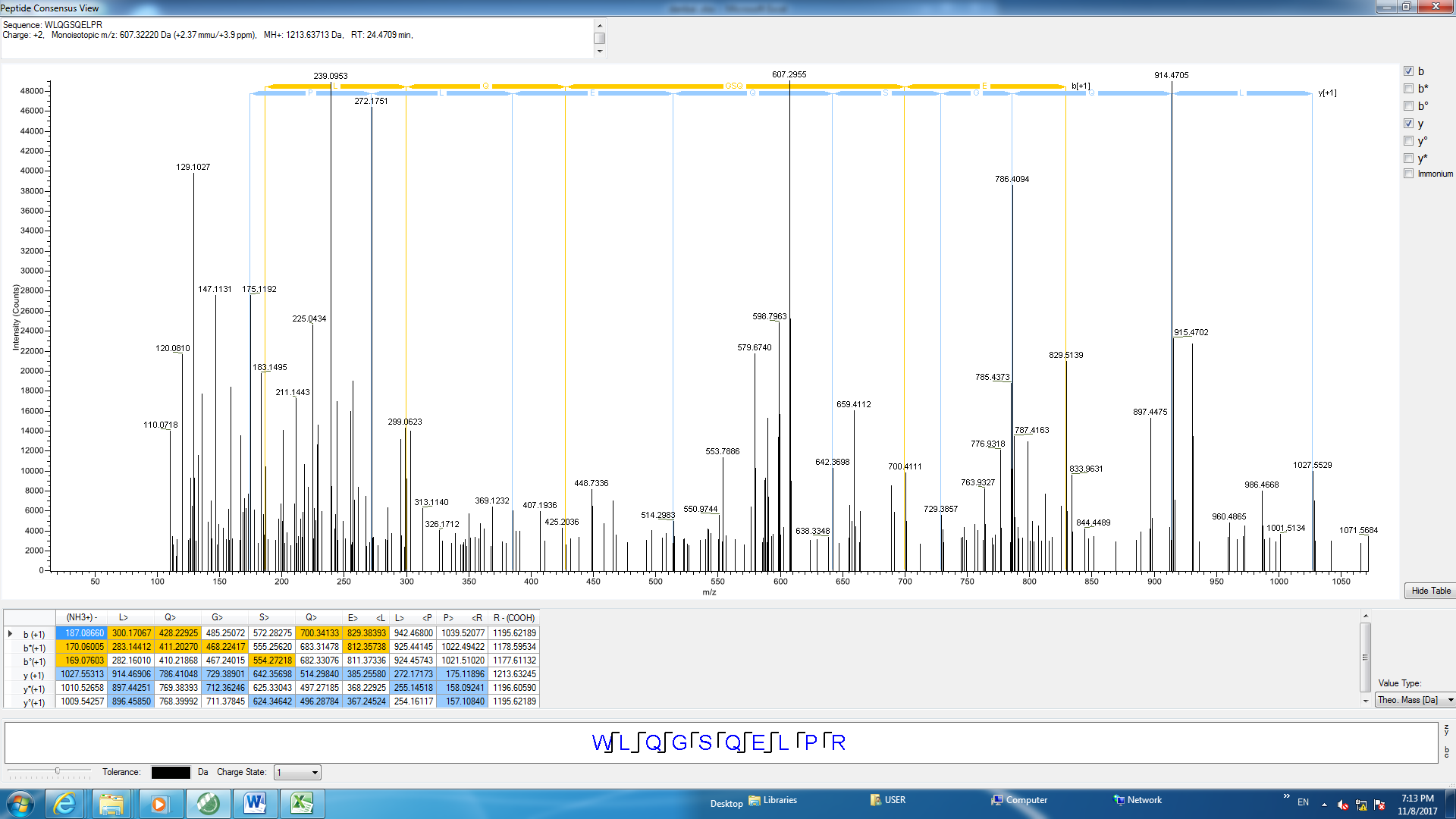


Q96DA0 Zymogen granule protein 16 homolog B (1/2)


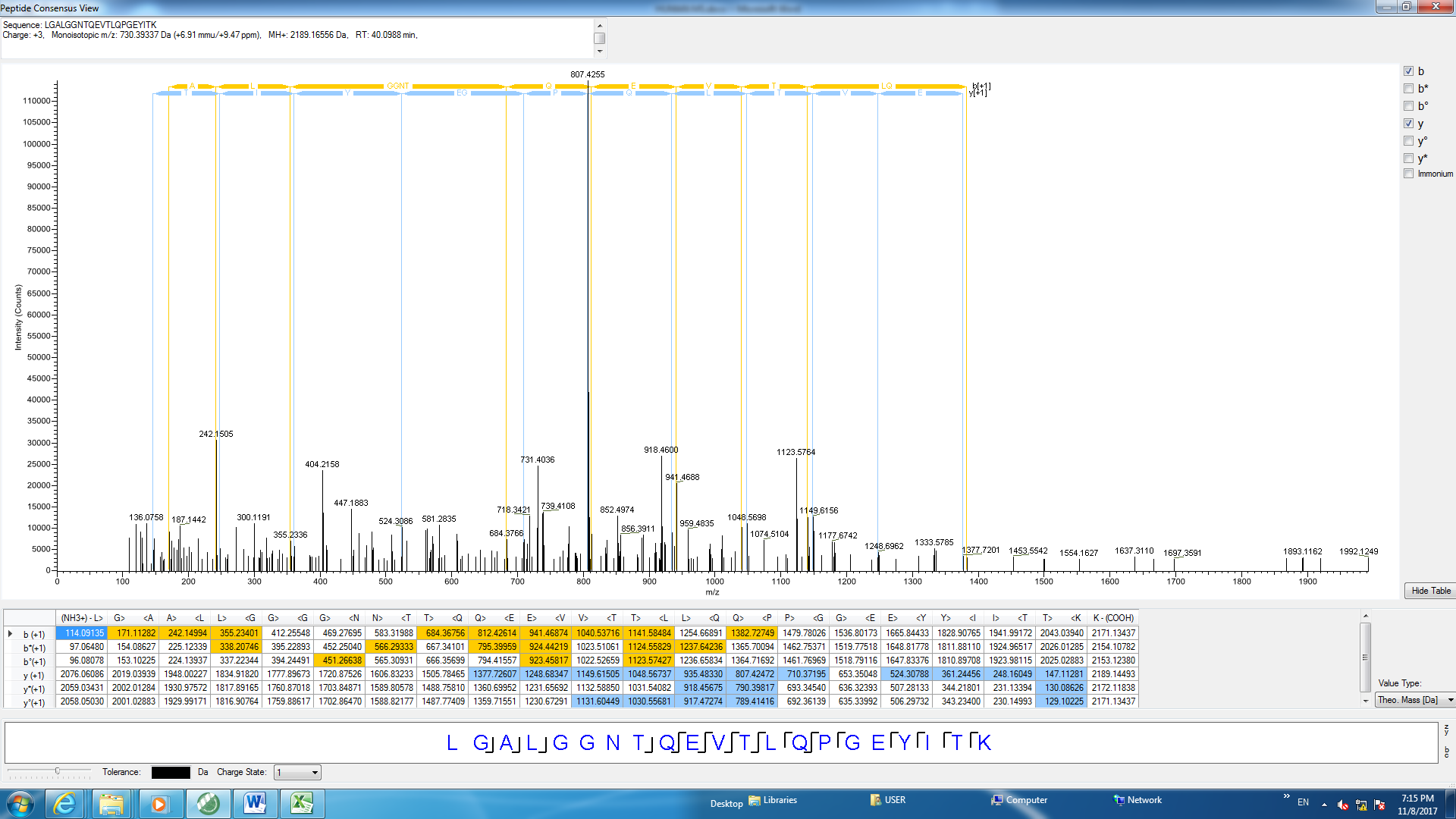


Q96DA0 Zymogen granule protein 16 homolog B (2/2)


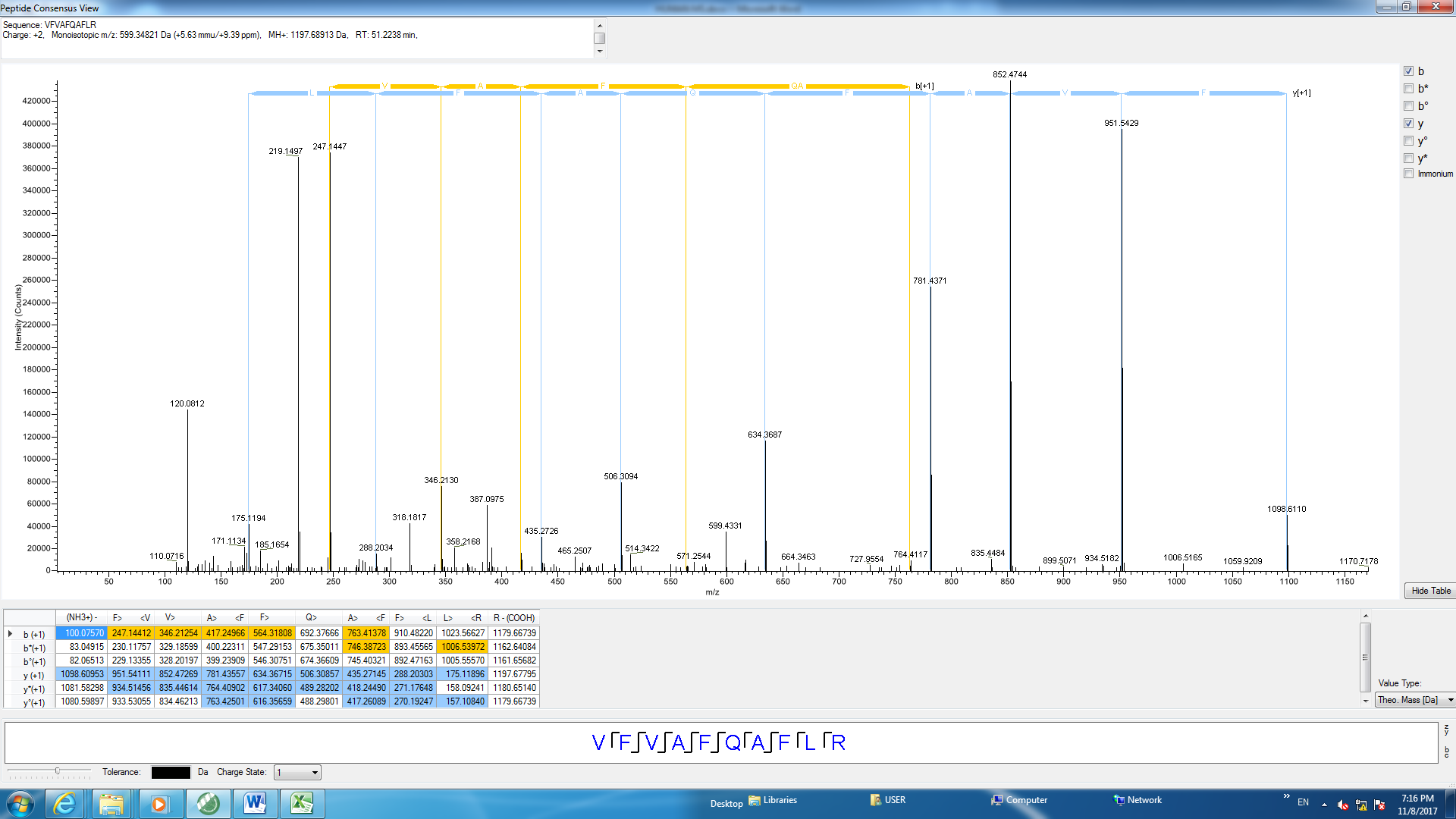

Supplement: Supplementary file 1 — Supplementary Information [file 41598_2019_41361_MOESM1_ESM.doc]
